# Haplotypic Associations and Differentiation of MHC Class II Polymorphic Alu Insertions at Five Loci With HLA-DRB1 Alleles in 12 Minority Ethnic Populations in China

**DOI:** 10.3389/fgene.2021.636236

**Published:** 2021-07-07

**Authors:** Yina Cun, Lei Shi, Jerzy K. Kulski, Shuyuan Liu, Jia Yang, Yufen Tao, Xinwen Zhang, Li Shi, Yufeng Yao

**Affiliations:** ^1^Department of Immunogenetics, Institute of Medical Biology, Chinese Academy of Medical Sciences and Peking Union Medical College, Kunming, China; ^2^Faculty of Health and Medical Sciences, University of Western Australia Medical School, Crawley, WA, Australia; ^3^Yunnan Key Laboratory of Vaccine Research and Development on Severe Infectious Disease, Institute of Medical Biology, Chinese Academy of Medical Sciences and Peking Union Medical College, Kunming, China

**Keywords:** HLA class II regions, POALIN, HLA-DRB1, polymorphism, haplotypes, Chinese ethnic populations

## Abstract

The analysis of polymorphic variations in the human major histocompatibility complex (MHC) class II genomic region on the short-arm of chromosome 6 is a scientific enquiry to better understand the diversity in population structure and the effects of evolutionary processes such as recombination, mutation, genetic drift, demographic history, and natural selection. In order to investigate associations between the polymorphisms of HLA-DRB1 gene and recent Alu insertions (POALINs) in the HLA class II region, we genotyped HLA-DRB1 and five Alu loci (AluDPB2, AluDQA2, AluDQA1, AluDRB1, AluORF10), and determined their allele frequencies and haplotypic associations in 12 minority ethnic populations in China. There were 42 different HLA-DRB1 alleles for ethnic Chinese ranging from 12 alleles in the Jinuo to 28 in the Yugur with only DRB1^∗^08:03, DRB1^∗^09:01, DRB1^∗^12:02, DRB1^∗^14:01, DRB1^∗^15:01, and DRB1^∗^15:02 present in all ethnic groups. The POALINs varied in frequency between 0.279 and 0.514 for AluDPB2, 0 and 0.127 for AluDQA2, 0.777 and 0.995 for AluDQA1, 0.1 and 0.455 for AluDRB1 and 0.084 and 0.368 for AluORF10. By comparing the data of the five-loci POALIN in 13 Chinese ethnic populations (including Han-Yunnan published data) against Japanese and Caucasian published data, marked differences were observed between the populations at the allelic or haplotypic levels. Five POALIN loci were in significant linkage disequilibrium with HLA-DRB1 in different populations and AluDQA1 had the highest percentage association with most of the HLA-DRB1 alleles, whereas the nearby AluDRB1 indel was strongly haplotypic for only DRB1^∗^01, DRB1^∗^10, DRB1^∗^15 and DRB1^∗^16. There were 30 five-locus POALIN haplotypes inferred in all populations with H5 (no Alu insertions except for AluDQA1) and H21 (only AluDPB2 and AluDQA1 insertions) as the two predominant haplotypes. Neighbor joining trees and principal component analyses of the Alu and HLA-DRB1 polymorphisms showed that genetic diversity of these genomic markers is associated strongly with the population characteristics of language family, migration and sociality. This comparative study of HLA-DRB1 alleles and multilocus, lineage POALIN frequencies of Chinese ethnic populations confirmed that POALINs whether investigated alone or together with the HLA class II alleles are informative genetic and evolutionary markers for the identification of allele and haplotype lineages and genetic variations within the same and/or different populations.

## Introduction

The human major histocompatibility complex (MHC) class II genomic region on the short-arm of chromosome 6 contains highly polymorphic classical and non-classical human leukocyte antigen (HLA) class II genes (HLA-DRB1, -DRA, -DQA1, -DQB1, -DQA2, -DQB2, -DPA1, and -DPB1) involved in the regulation of the innate and adaptive immune system, autoimmunity, and transplantation (Shiina et al., 2004, 2009; Vandiedonck and Knight, 2009; Trowsdale, 2011). The extensive polymorphism of the HLA class II genes is studied widely and used to provide a better understanding of the diversity in population structure and the effects of evolutionary processes such as recombination, mutation, genetic drift, demographic history, and natural selection (Meyer et al., 2006; Traherne, 2008; Pierini and Lenz, 2018; Manczinger et al., 2019). For example, there are at least 2,909 HLA-DRB1 alleles distributed world-wide with the official sequences and designations provided by the IMGT/HLA database (Robinson et al., 2020). Consequently, the HLA-DRB1 alleles are genetic markers that are utilized often for the assessment of population structure and differentiation as well as providing information on interpopulation genetic exchange (gene flow) and other demographic events (Di and Sanchez-Mazas, 2011; Sanchez-Mazas et al., 2013, 2017; Sanchez-Mazas and Meyer, 2014; Gonzalez-Galarza et al., 2020). Moreover, the HLA-DRB1 alleles present intracellular or exogenous antigen peptides to CD4^+^ T cells that trigger and regulate the downstream immune responses to defend against pathogen invasion (Chaplin, 2010). Therefore, this highly polymorphic genomic marker might reveal changes associated with pathogen-mediated pressure on highly heterogenous and diverse populations (Sun et al., 2015; Weiskopf et al., 2016).

In addition to polymorphic HLA class II genes, the MHC class II region has a number of polymorphic Alu insertions (POALINs) that are informative population ancestral lineage markers. They are insertion/deletions (either present or absent) at integration sites, which carry characteristic alleles or haplotypes inherited from different ancestral populations (Bennett et al., 2004; Kulski and Dunn, 2005; Ray et al., 2007). Alu retroelements (short interspersed nuclear elements) are among the class of genomic repetitive DNA elements that first appeared in primates about 65 million years ago and then amplified by retrotransposition to the present estimated one million copies per human genome (Lander et al., 2001; Batzer and Deininger, 2002). POALINs are useful lineage and evolutionary genetic markers for studying the origin and genomic diversity of human populations because (1) their allelic frequency distributions vary significantly among geographically different human populations (Deininger and Batzer, 1999; Jorde et al., 2000; Watkins et al., 2001), and (2) they have an inherited identity by descent arising from a known initial ancestral state (no Alu insertion), whereby their presence and/or absence define the ancestral lineages within a population (Antunez-de-Mayolo et al., 2002).

Some MHC Alu family members were used previously as evolutionary molecular markers to infer the ancestral duplication history of HLA class I and class II gene copies (Mnukova-Fajdelova et al., 1994; Svensson et al., 1996; Kulski et al., 1999, 2000). Also, several studies reported on the frequencies and distribution of human-specific POALIN loci within the HLA class I region and on their inferred haplotypic associations with HLA-A, -B and -C loci in different populations (Dunn et al., 2002, 2003, 2005a,b, 2007; Yao et al., 2009, 2010; Kulski et al., 2011, 2019; Mastana et al., 2017; Singh et al., 2019). These associations reflect in part the different haplotypic structures of the MHC class I and class II regions and the linkage of multiple polymorphic loci, especially when extended over long stretches (1–3 Mb) of conserved genomic sequences in human populations known as ancestral haplotypes (Dawkins et al., 1999) or conserved extended haplotypes (Alper et al., 2006; Larsen et al., 2014). Although comparative DNA sequence analysis of the entire MHC genome region between two homozygous HLA haplotypes has indicated the presence of POALIN within the MHC class II region (Stewart et al., 2004), five human-specific POALIN (AluDPB2, AluDQA2, AluDQA1, AluDRB1, and AluORF10) frequencies at five loci in the MHC class II genomic region were determined previously only for Japanese, Australian Caucasians (Kulski et al., 2010) and Chinese Han in Yunnan province (Shi et al., 2014) populations. By comparing the data of the MHC class II five-loci POALINs in Chinese Han with Japanese and Caucasian data, marked differences were observed between the three ethnic groups at the allelic or haplotypic levels. In addition, each POALIN was in significant linkage disequilibrium (LD) and/or haplotypically associated (Kulski et al., 2020, 2021) with a variety of HLA-DRB1 alleles in Chinese Han in Yunnan province (Shi et al., 2014). These results showed that POALINs whether investigated alone or together with the HLA class II alleles are informative genetic markers for the identification of allele and haplotype lineages and variations within the same and/or different populations.

Beside the Chinese speaking Han majority, there are 55 officially recognized minority ethnic populations of China, which contribute to about 8% of the overall Chinese population and provide abundant genetic resources for POALIN–HLA inferred haplotype studies (Yao et al., 2010). The minority ethnic groups living in the south and southwest of China can be traced back to three major ancient groups: Di-Qiang, Bai-Pu, and Bai-Yue that speak the Tibeto-Burman, Mon-Khmer and Daic language subfamilies, respectively (Table 1); whereas in the northwest of China, most ethnic groups speak the language of the Mongolian and Tujue Manchu-Tungusic subfamily, which is the Altaic language family (Guo, 2000). Although the anthropological, cultural and linguistic characteristics of some of these ethnic populations have been studied in detail (You, 1994; Guo, 2000; Chu et al., 2006), there are few published comparative investigations on the genetic diversity of these populations by genome-wide sequencing or genotyping methods (Di and Sanchez-Mazas, 2011). Therefore, the analyses of robust and reproducible genetic markers such as the POALINs and HLA-DRB1 alleles in small and isolated ethnic minority remains an important task to better understand the human genome and its genetic variability throughout the world.

**TABLE 1:** Geographic and language information for the 12 minority ethnic populations sampled in the current study.

Ethnic group	Sample size	Sample location	Ancient tribe	Language family	Language subfamily	Population size
Hani	149	Jingha village, Jinghong County, Yunnan Province, Southwest China	Di-Qiang (dq)	Sino-Tibetan	Tibeto-Burman (TB)	1,661,000
Jinuo	75	Jinuo Mountains Area, Jinghong City, Xishuangbanna Prefecture, Yunnan Province, Southwest China	Di-Qiang (dq)	Sino-Tibetan	Tibeto-Burman (TB)	23,143
Lisu	79	Fugong and Gongshan Counties, Nujiang Municipality, Yunnan Province, Southwest China	Di-Qiang (dq)	Sino-Tibetan	Tibeto-Burman (TB)	703,000
Nu	82	Fugong and Gongshan Counties, Nujiang Municipality, Yunnan Province, Southwest China	Di-Qiang (dq)	Sino-Tibetan	Tibeto-Burman (TB)	37,523
Jingpo	95	Dehong Dai-Jingpo Autonomous Prefectures, Yunnan Province, Southwest China	Di-Qiang (dq)	Sino-Tibetan	Tibeto-Burman (TB)	147,828
Bulang	109	Bulang Mountain Area, Menghai County, Yunnan Province, Southwest China	Baipu (bp)	Austo-Asiatic	Mon-Khmer (MK)	120,000
Wa	109	Canyuan and Ximeng Counties, Yunnan Province, Southwest China	Baipu (bp)	Austo-Asiatic	Mon-Khmer (MK)	430,000
Dai	121	Xishuangbanna Dai Autonomous Prefecture, Yunnan Province, Southwest China	Baiyue (by)	Sino-Tibetan	Daic (D)	1,261,000
Maonan	78	Xianan village of Huangjiang County, Guangxi Province, Southern China	Baiyue (by)	Sino-Tibetan	Daic (D)	101,000
Zhuang	101	Tiandeng County, Nanning City, Guangxi Zhuang Autonomous Province, Southern China	Baiyue (by)	Sino-Tibetan	Daic (D)	16,926,000
Tu	110	Huzu County, Qinghai Province, Northwest China	Mongolian (m)	Altaic	Mongolian (M)	290,000
Yugur	93	Sunan County, Gansu Province, Northwest China	Mongolian (m)	Altaic	Tujue (T)	14,378

The aim of present study was to elucidate the inferred haplotypic association between the MHC POALINs and classical HLA class II alleles by determining (1) genetic structures of the five MHC class II POALIN dimorphisms and HLA-DRB1 allele and haplotype frequencies in 12 minority ethnic populations in China, and (2) correlations between the genetic diversity and the four language families of these populations (Table 1). Among these 12 minority ethnic populations, 8 of them settled in Yunnan province together with the Han people (Han-Yunnan). The Han-Yunnan, speaking Chinese of the Sino-Tibetan language family, migrated from the northern region by various routes and at different times to Yunnan province and exhibited genetic characteristics of both northern and southern Chinese groups (Shi et al., 2006). Thus, we included the published data of Han-Yunnan, Japanese and Caucasians as reference populations in order to compare and correlate the genetic differentiation of the HLA-DRB1 alleles and the five POALINs between the populations and the language families by using the DA genetic distance measure in phylogeny and principal component analysis (PCA).

## Materials and Methods

### Ethics Statement

This study was approved by the Committee on the Ethics of Institute of Medical Biology, Chinese Academy of Medical Sciences, the batch number is YIKESHENGLUNZI [2012]12. Moreover, the protocol employed by this investigation was in accordance with the principles expressed in the Helsinki Declaration of 1975, which was revised in 2008. Written informed consents were obtained from each participant.

### Subjects and Samples

**FIGURE 1:**
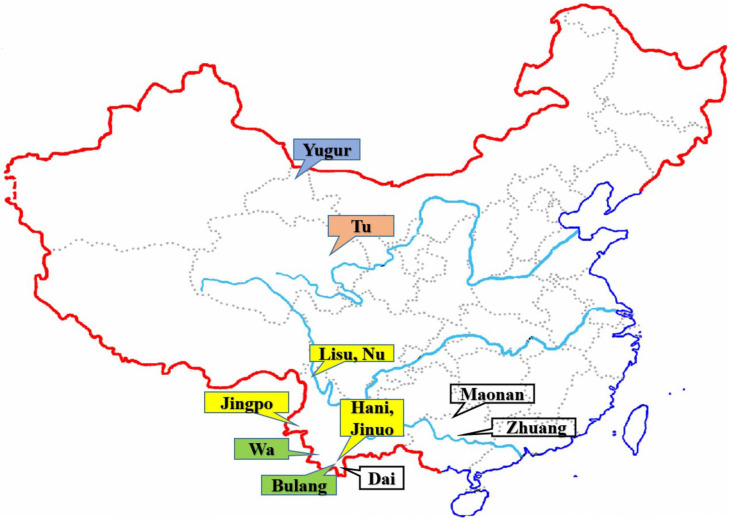
The geographic locations of the 12 Chinese ethnic populations in China. The colored labeled boxes represent the ancient tribe, language family and subfamily for each population listed in Table 1. Yellow represent Di-Qiang, Sino-Tibetan, Tibeto-Burman. Green represent Baipu, Austo-Asiatic, Mon-Khmer. White represent Baiyue, Sino-Tibetan, Daic. Orange represent Mongolian, Altaic, Mongolian. Blue represent Mongolian, Altaic, Tujue.

A total of 1,201 unrelated individuals were recruited from 12 Chinese minority ethnic populations in China (Figure 1). The geographic location, sample size of each population, the language family to which they belong, and the ancient groups from which they originated are listed in Table 1. These populations are descended from four ancient Chinese groups and belong to four different language subfamilies (Guo, 2000; Yao et al., 2010; Di and Sanchez-Mazas, 2011) as outlined in the introduction and Table 1. The geographic origin, nationalities, and pedigree (unrelated through at least three generations) of each individual were ascertained before sampling.

### Genomic DNA and HLA-DRB1 Typing

Genomic DNA was extracted from peripheral lymphocytes using a QIAamp Blood Kit (Qiagen, Hilden, Germany). DNA samples were quantified with a NanoDrop ND-1000 spectrophotometer (NanoDrop Technologies, Wilmington, WI, United States) and adjusted to a concentration of 20 ng/L. The HLA- DRB1 genes were genotyped using a WAKFlow HLA typing kit (Wakunaga, Hiroshima, Japan) as in previous studies (Ogata et al., 2007; Shi et al., 2008, 2010a,b, 2011; Yao et al., 2012; Tao et al., 2020), which is based on polymerase chain reaction-sequence specific oligonucleotide probes (PCR-SSOP) coupled with multiple analyte profiling (xMAP) technology (Luminex System).

### Alu and PCR Assay

The sense and antisense primers used for the PCR of the POALINs located in MHC II regions were previously reported (Kulski et al., 2010; Shi et al., 2014). As some of the previously published primers used for the PCR of the POALINs located in MHC II region have mutations in the Chinese Han in Yunnan province, new sense and antisense primer pairs were designed and used for the PCR of five POALINs located in the MHC II region (Supplementary Table 1). Supplementary Figure 1 shows a map of the locations of the five POALINs with the HLA class II regions of the MHC on chromosome 6p21.3.

The PCR products were analyzed according to the fragments of different sizes by the presence or absence of an electrophoretic specific band in 2% agarose gel stained with ethidium bromide and visualized by ultraviolet light. The Alu-PCR methods clearly differentiate between an insertion and absence of insertion in heterozygous individuals based on distinctly different sized PCR products as shown in Supplementary Figure 2. The POALIN alleles are dimorphic structures whereby the absence of the Alu insertion at the Alu locus is the Alu^∗^1 allele and the presence of the Alu insertion is the Alu^∗^2 allele. The overall frequencies of the Alu^∗^2 (insertion) allele at each of the five loci were estimated from the genotypes as described below in the statistical section.

### Allele Linkage Controls for Assessment of HLA-DRB1 Allele and POALIN Associations

To better assess the haplotypic associations between the POALINs and the HLA-DRB1 alleles, we examined their sequence linkages in 95 different MHC haplotype sequences (Kulski et al., 2021) that were sequenced, partially annotated and assembled from HLA-homozygous cell lines by Norman et al. (2017).

The FASTA files of the 95 MHC class I, II and III genomic sequences were downloaded from the archives at NCBI BioProject with the accession number PRJEB6763^1^ and submitted to the RepeatMasker webserver^2^ for output files of annotated members of the interspersed repetitive DNA families, their locations in the sequence and their relative similarity or identity in comparison to reference sequences of SINEs, LINEs, LTRs, ERVs, DNA elements, small RNA, and simple repeats (Kulski et al., 2021). The five MHC class II POALINs were easily identified within the RepeatMasker outputs on the basis of their location and flanking sequences and/or other repeats as previously described (Kulski et al., 2010). The HLA-DRB1 alleles for all of the 95 cell line sequences were determined and reported by Norman et al. (2017). Supplementary Table 2 is a summary of the sequence linkages between the 5 POALIN and the HLA-DRB1 alleles that were determined in 90 of the sequenced haplotypes (Kulski et al., 2021). These were used as a comparative reference control to assist with a better interpretation of our results obtained for our haplotypic association analyses in 15 different populations.

### Statistical Analysis

The frequencies of five POALINs were calculated from the genotyping data by the direct-counting method. For each locus, Hardy-Weinberg’s equilibrium was assessed using the Guo and Thompson method (Guo and Thompson, 1992). The haplotypes were estimated by the maximum-likelihood method using the Pypop software (Lancaster et al., 2003, 2007). Pairwise LD of POALINs and HLA allele were calculated using the SHEsis software^3^ (Shi and He, 2005). The percentage association between a POALIN insertion and an HLA allele was calculated as the percentage of the total HLA allele frequency that was associated with the presence of the POALIN insertion at an inferred HLA class II gene/POALIN haplotype using the haplotype frequency data generated by the Pypop software (Lancaster et al., 2003, 2007). Percentage associations between HLA allele and POALIN insertion frequencies were considered to be very strong if between 80 and 100%, strong if over 50% and less than 79%, moderate if between 20 and 50%, and low or absent if less than 20% (Kulski et al., 2010; Shi et al., 2014). The differences in significance between the POALIN and its haplotype frequencies were determined by a contingency test (Fisher’s exact test). Bonferroni correction was used for multiple testing. Statistical significance was defined at the 5% level.

### Phylogenetic Analysis

Based on the POALIN allele, HLA-DRB1 allele frequencies and DRB1/AluDRB1 haplotypes of the different population, the DA was calculated using the Dispan software (Nei, 1973, 1978). The Mega 7.0 software was used to reconstruct the neighbor-joining (NJ) trees according to the DA (Tamura et al., 2007). Principal component analysis (PCA) was also performed based either on POALIN allele, or HLA-DRB1 frequencies using SPSS 16.0 software. POALIN allele and HLA-DRB1 allele frequencies were obtained from additional Japanese, Caucasian and Han-Yunnan populations (Kulski et al., 2010; Shi et al., 2014) for comparative phylogenetic analysis with the frequencies obtained for the 12 Chinese ethnic populations in this study.

## Results

### HLA-DRB1 Allele Frequencies

We summarized the HLA-DRB1 allele frequencies in 15 populations in Table 2 according to previous studies (Shi et al., 2006, 2008, 2010a,b, 2011; Ogata et al., 2007; Kulski et al., 2010; Yao et al., 2012; Tao et al., 2020). There were 57 different HLA-DRB1 alleles for the 15 populations ranging from 12 alleles in the Jinuo to 39 alleles in the Han-Yunnan. Only six HLA-DRB1 alleles were present in all 15 populations and these were DRB1^∗^08:03, DRB1^∗^09:01, DRB1^∗^12:02, DRB1^∗^14:01, DRB1^∗^15:01, and DRB1^∗^15:02. There were sixteen low frequency, unique, solitary alleles for six populations; DRB1^∗^03:05 (0.003), DRB1^∗^08:27 (0.003), DRB1^∗^09:09 (0.003), DRB1^∗^11:31 (0.003), DRB1^∗^11:52 (0.003), DRB1^∗^12:19 (0.003), DRB1^∗^13:28 (0.003), DRB1^∗^14:32 (0.005) and DRB1^∗^14:35 (0.003) in Han-Yunnan, DRB1^∗^01:03 (0.011), DRB1^∗^08:10 (0.003) and DRB1^∗^11:03 (0.017) in Caucasians, DRB1^∗^14:06 (0.01) in Japanese, DRB1^∗^14:18 (0.015) in Zhuang, DRB1^∗^14:25 (0.014) in Bulang, and DRB1^∗^15:11 (0.011) in Jingo. The successive highest frequencies of DRB1 alleles were DRB1^∗^16:02 in Dai, and DRB1^∗^15:01 in Zhuang, and DRB1^∗^14:01 and DRB1^∗^16:02 in the Maonan. In addition, DRB1^∗^12:02 was the most frequent in the Hani, Jinuo, Lisu, Nu, Jingo, Bulang, Wa, Maonan and Han-Yunnan ranging from 16% in Maonan to 55% in Bulang. The highest allelic frequency in the two Mongolian groups, Tu and Yugur, was DRB1^∗^09:01 (12.7% and 13.4%, respectively) as same as Japanese (20%).

**TABLE 2:** HLA-DRB1 allele frequencies in 15 populations.

No.	DRB1 allele	Hani	Jinuo	Lisu	Nu	Jingpo	Bulang	Wa	Dai	Maonan	Zhuang	Tu	Yugur	Han-Yunnan	Japanese	Caucasians
		(*n* = 149)	(*n* = 75)	(*n* = 79)	(*n* = 82)	(*n* = 95)	(*n* = 109)	(*n* = 109)	(*n* = 121)	(*n* = 78)	(*n* = 101)	(*n* = 110)	(*n* = 93)	(*n* = 186)	(*n* = 100)	(*n* = 174)
1	DRB1*01:01	0.013		0.013	0.006	0.026						0.064	0.048	0.024	0.08	0.095
2	DRB1*01:02			0.006												0.014
3	DRB1*01:03															0.011
4	DRB1*03:01	0.007						0.018	0.058	0.045	0.129	0.023	0.065	0.035		0.126
5	DRB1*03:05													0.003		
6	DRB1*04:01			0.006			0.005					0.027	0.043	0.003	0.02	0.109
7	DRB1*04:02												0.005	0.003		0.006
8	DRB1*04:03	0.017	0.04		0.018	0.005	0.018	0.014	0.012	0.006		0.005	0.038	0.016	0.035	0.006
9	DRB1*04:04									0.013		0.005	0.016	0.003		0.043
10	DRB1*04:05			0.038	0.024	0.026	0.023	0.028	0.054	0.09	0.015	0.068	0.054	0.059	0.135	
11	DRB1*04:06			0.006	0.03	0.011	0.005	0.014	0.021	0.006	0.015	0.014	0.022	0.027	0.025	
12	DRB1*04:07			0.006											0.01	0.02
13	DRB1*04:08												0.011		0.01	0.003
14	DRB1*04:10			0.019	0.006			0.009				0.009		0.008	0.01	
15	DRB1*07:01	0.003	0.013	0.006	0.012	0.021	0.005	0.055	0.008			0.073	0.065	0.062		0.144
16	DRB1*08:01	0.003											0.016			0.014
17	DRB1*08:02			0.006	0.012							0.005	0.022	0.008	0.035	
18	DRB1*08:03	0.03	0.087	0.076	0.11	0.047	0.014	0.037	0.004	0.071	0.015	0.059	0.027	0.089	0.07	
19	DRB1*08:04												0.011			0.003
20	DRB1*08:09								0.004				0.005			
21	DRB1*08:10															0.003
22	DRB1*08:27													0.003		
23	DRB1*09:01	0.027	0.007	0.051	0.024	0.042	0.014	0.005	0.112	0.103	0.05	0.127	0.134	0.159	0.2	0.023
24	DRB1*09:09													0.003		
25	DRB1*10:01	0.003				0.016			0.008		0.02	0.045	0.054	0.003	0.005	0.006
26	DRB1*11:01	0.013	0.047	0.044	0.031	0.021		0.005	0.021	0.026	0.05	0.105	0.048	0.054	0.01	0.06
27	DRB1*11:03															0.017
28	DRB1*11:04											0.005	0.005	0.008		0.006
29	DRB1*11:06			0.006	0.037	0.016	0.05	0.018	0.004							
30	DRB1*11:31													0.003		
31	DRB1*11:52													0.003		
32	DRB1*12:01	0.003		0.006	0.018							0.077	0.054	0.011	0.02	0.026
33	DRB1*12:02	0.322	0.407	0.209	0.238	0.453	0.55	0.326	0.099	0.16	0.045	0.086	0.054	0.164	0.025	
34	DRB1*12:19													0.003		
35	DRB1*13:01			0.019	0.012			0.073			0.005	0.014	0.043	0.011		0.063
36	DRB1*13:02			0.013	0.006	0.011	0.005		0.025		0.015	0.018		0.038	0.03	0.049
37	DRB1*13:03		0.007		0.012		0.005		0.037	0.064	0.025					0.006
38	DRB1*13:28													0.003		
39	DRB1*14:01	0.208	0.153	0.108	0.14	0.026	0.032	0.014	0.112	0.122	0.139	0.045	0.043	0.027	0.04	0.017
40	DRB1*14:02											0.009		0.003		
41	DRB1*14:03				0.018							0.014	0.005	0.008	0.03	
42	DRB1*14:04	0.118		0.12	0.012	0.021	0.005	0.069	0.033	0.006	0.005			0.005		
43	DRB1*14:05	0.02	0.027						0.012	0.013	0.005	0.027	0.016	0.008	0.015	
44	DRB1*14:06														0.01	
45	DRB1*14:07			0.019										0.003		
46	DRB1*14:10	0.017		0.013												
47	DRB1*14:18										0.015					
48	DRB1*14:25						0.014									
49	DRB1*14:32													0.005		
50	DRB1*14:35													0.003		
51	DRB1*15:01	0.044	0.04	0.057	0.037	0.058	0.073	0.092	0.087	0.096	0.168	0.018	0.065	0.073	0.05	0.103
52	DRB1*15:02	0.07	0.167	0.108	0.085	0.1	0.124	0.087	0.136	0.058	0.139	0.041	0.022	0.035	0.125	0.011
53	DRB1*15:04	0.067	0.007	0.038	0.098	0.063	0.037	0.133						0.008		
54	DRB1*15:11					0.011										
55	DRB1*15:15				0.006	0.005										
56	DRB1*16:01			0.006								0.009				0.014
57	DRB1*16:02	0.013			0.006	0.021	0.023	0.005	0.153	0.122	0.149	0.009	0.011	0.022	0.01	

*References: (Shi et al., 2006; Han-Yunnan); (Ogata et al., 2007; Maonan); (Shi et al., 2008; Jinuo, Wa); (Kulski et al., 2010; Japanese, Caucasians); (Shi et al., 2010a; Hani, Bulang); (Shi et al., 2010b; Dai); (Shi et al., 2011; Zhuang); (Yao et al., 2012; Lisu, Nu); (Tao et al., 2020; Jingpo).*

### POALIN Allele Frequencies and Hardy-Weinberg’s Equilibrium (HWE)

The five POALIN allele frequencies and the genotype counts in 12 Chinese minority populations, shown in Table 3, were compared statistically to those reported previously for the Japanese, Australian Caucasians (Kulski et al., 2010) and Chinese Han in Yunnan (Shi et al., 2014). The frequencies of five POALINs in 12 Chinese minority populations ranged from 0.359 to 0.514 (AluDPB2), 0 to 0.127 (AluDQA2), 0.777 to 0.995 (AluDQA1), 0.1 to 0.455 (AluDRB1) and 0.084 to 0.368 (AluORF10). The differences in significance between two populations for each POALIN frequency are shown in Supplementary Table 3.

**TABLE 3:** The MHC POALIN allelic frequencies and genotype counts at five loci in 15 populations.

Alu allele or genotype	Alu allele frequency or genotype counts in 15 populations (n)														

	Hani (*n* = 149)	Jinuo (*n* = 75)	Lisu (*n* = 79)	Nu (*n* = 82)	Jingpo (*n* = 95)	Bulang (*n* = 109)	Wa (*n* = 109)	Dai (*n* = 121)	Maonan (*n* = 78)	Zhuang (*n* = 101)	Tu (*n* = 110)	Yugur (*n* = 93)	Han-Yunnan (*n* = 186)^a^	Japanese (*n* = 100)^b^	Caucasians (*n* = 174)^b^
Alu allele	Frequency														
AluDPB2*1	0.507	0.620	0.544	0.585	0.721	0.583	0.560	0.545	0.641	0.574	0.486	0.527	0.540	0.500	0.546
AluDPB2*2	0.493	0.380	0.456	0.415	0.279	0.417	0.440	0.455	0.359	0.426	0.514	0.473	0.460	0.500	0.454
AluDQA2*1	0.997	0.987	0.981	0.994	0.895	0.977	0.995	0.959	1.000	0.975	0.873	0.909	0.997	0.990	0.790
AluDQA2*2	0.003	0.013	0.019	0.006	0.105	0.023	0.005	0.041	0.000	0.025	0.127	0.091	0.003	0.010	0.210
AluDQA1*1	0.124	0.067	0.184	0.195	0.032	0.005	0.106	0.050	0.026	0.134	0.223	0.097	0.312	0.432	0.503
AluDQA1*2	0.876	0.933	0.816	0.805	0.968	0.995	0.894	0.950	0.974	0.866	0.777	0.903	0.688	0.568	0.497
AluDRB1*1	0.789	0.780	0.804	0.768	0.753	0.739	0.683	0.632	0.705	0.545	0.900	0.855	0.820	0.775	0.744
AluDRB1*2	0.211	0.220	0.196	0.232	0.247	0.261	0.317	0.368	0.295	0.455	0.100	0.145	0.180	0.225	0.256
AluORF10*1	0.916	0.900	0.873	0.896	0.826	0.830	0.862	0.632	0.641	0.634	0.859	0.882	0.825	0.855	0.764
AluORF10*2	0.084	0.100	0.127	0.104	0.174	0.170	0.138	0.368	0.359	0.366	0.141	0.118	0.175	0.145	0.236
**Alu genotype**	**Counts**														
AluDPB2 1, 1	38	29	24	29	50	44	35	37	34	30	28	23	57		
AluDPB2 1, 2	75	35	38	38	37	39	52	58	32	56	51	52	87		
AluDPB2 2, 2	36	11	17	15	8	26	22	26	12	15	31	18	42		
AluDQA2 1, 1	148	74	77	81	76	104	108	113	78	96	84	81	185		
AluDQA2 1, 2	1	0	1	1	18	5	1	6	0	5	24	7	1		
AluDQA2 2, 2	0	1	1	0	1	0	0	2	0	0	2	5	0		
AluDQA1 1, 1	14	3	13	15	3	0	5	4	1	13	21	5	28		
AluDQA1 1, 2	9	4	3	2	0	1	13	4	2	1	7	8	60		
AluDQA1 2, 2	126	68	63	65	92	108	91	113	75	87	82	80	98		
AluDRB1 1, 1	95	46	51	49	52	59	50	45	37	27	89	68	124		
AluDRB1 1, 2	45	25	25	28	39	43	49	63	36	56	20	23	57		
AluDRB1 2, 2	9	4	3	5	4	7	10	13	5	18	1	2	5		
AluORF10 1, 1	128	61	59	67	65	76	80	46	34	39	82	75	128		
AluORF10 1, 2	17	13	20	13	27	29	28	61	32	50	25	14	51		
AluORF10 2, 2	4	1	0	2	3	4	1	14	12	12	3	4	7		

*^*a*^The data of five POALINs in Han-Yunnan were published previously (Shi et al., 2014).*

*^*b*^The data of five POALINs in Japanese and Caucasians were published previously (Kulski et al., 2010, 2011).*

Of all the five POALIN loci, the AluDQA1 locus showed a significant departure (*P* < 0.01 after Bonferroni’s correction) from HWE in 10 minority populations, which were the Hani, Jinuo, Lisu, Nu, Jingpo, Wa, Dai, Zhuang, Tu and Yugur (Supplementary Table 4). The data were similar to the results of Han in Yunnan (Shi et al., 2014) and Japanese (Kulski et al., 2010) that also showed that the AluDQA1 locus was not consistent with the HWE. The AluDPB2 locus showed a significant departure (*P* < 0.01 after Bonferroni’s correction) from HWE in the Bulang; whereas the AluDQA2 locus showed a significant departure (*P* < 0.01 after Bonferroni’s correction) from HWE in the Jinuo and Yugur.

### POALIN Haplotype Frequencies

Table 4 shows the POALIN haplotypes for 12 Chinese minority populations, the Chinese Han in Yunnan (Shi et al., 2014), Japanese and Caucasians (Kulski et al., 2010). There were 30 five-locus POALIN haplotypes inferred in all 15 populations, with 11 in Hani, 11 in Jinuo, 15 in Lisu, 12 in Nu, 14 in Jingpo, 10 in Bulang, 12 in Wa, 16 in Dai, 11 in Maonan, 16 in Zhuang, 19 in Tu, 18 in Yugur, 14 in Han-Yunnan, 14 in Japanese and 23 in Caucasians. All haplotypes were named H1-H30 and only five haplotypes were found in all 15 populations. These were the ancestral null H1 with no Alu insertions (AluDPB2^∗^1: AluDQA2^∗^1: AluDQA1^∗^1: AluDRB1^∗^1: AluORF10^∗^1), and various haplotypes with one to three Alu insertions; H5 (AluDQA1^∗^2), H7 (AluDQA1^∗^2: AluDRB1^∗^2), H21 (AluDPB2^∗^2: AluDQA1^∗^2) and H23 (AluDPB2^∗^2: AluDQA1^∗^2: AluDRB1^∗^2). The H5 (AluDQA1^∗^2) and H21 (AluDPB2^∗^2: AluDQA1^∗^2) haplotypes were predominant in all 12 minority populations at frequency ranges of 0.144–0.433 and 0.158–0.352, respectively, which was the same as that for the Han-Yunnan, Japanese and Caucasians. There were seven haplotypes that were specific to only one particular population. These were three two-insertion haplotypes, three three-insertion haplotypes, and one five-insertion haplotype; H4 (AluDRB1^∗^2: AluORF10^∗^2) in the Japanese, H10 (AluDQA2^∗^2: AluORF10^∗^2), H12 (AluDQA2^∗^2: AluDRB1^∗^2: AluORF10^∗^2), H20 (AluDPB2^∗^2: AluDRB1^∗^2: AluORF10^∗^2), and H26 (AluDPB2^∗^2: AluDQA2^∗^2: AluDRB1^∗^2) in Caucasians, H11 (AluDQA2^∗^2: AluDRB1^∗^2) in Hani, and H30 (AluDPB2^∗^2: AluDQA2^∗^2: AluDQA1^∗^2: AluDRB1^∗^2: AluORF10^∗^2) in the Tu. The differences in significance between two populations for each haplotype frequency are shown in Supplementary Table 5.

**TABLE 4:** Haplotype frequencies of POALINs at five loci in 15 populations.

5-loci Alu haplotypes						Haplotype frequencies in 15 populations														
No.	Alu DPB2	Alu DQA2	Alu DQA1	Alu DRB1	Alu ORF10	Hani	Jinuo	Lisu	Nu	Jingpo	Bulang	Wa	Dai	Maonan	Zhuang	Tu	Yugur	Han-Yunnan^a^	Japanese^b^	Caucasians^b^
H1	1	1	1	1	1	0.016	0.040	0.059	0.063	0.005	0.005	0.022	0.009	0.007	0.043	0.075	0.038	0.102	0.176	0.140
H2	1	1	1	1	2			0.006	0.010	0.011		0.009	0.015		0.056	0.012	0.005	0.031		
H3	1	1	1	2	1	0.007		0.006	0.006							0.016	0.006	0.014	0.021	0.040
H4	1	1	1	2	2														0.006	
H5	1	1	2	1	1	0.332	0.386	0.335	0.283	0.433	0.311	0.343	0.229	0.293	0.144	0.221	0.305	0.264	0.126	0.153
H6	1	1	2	1	2			0.010	0.050	0.037	0.063	0.020	0.062	0.155	0.031	0.040	0.052	0.039	0.051	0.023
H7	1	1	2	2	1	0.073	0.107	0.071	0.167	0.081	0.156	0.117	0.083	0.090	0.141	0.043	0.072	0.047	0.106	0.021
H8	1	1	2	2	2	0.075	0.074	0.043		0.092	0.025	0.049	0.125	0.096	0.140	0.010	0.004	0.040		0.033
H9	1	2	1	1	1			0.006					0.011			0.016	0.018		0.010	0.080
H10	1	2	1	1	2								0.000							0.004
H11	1	2	1	2	1	0.003											0.002			
H12	1	2	1	2	2															0.005
H13	1	2	2	1	1			0.006	0.006	0.052			0.006		0.010	0.050	0.020	0.003		0.013
H14	1	2	2	1	2										0.004	0.003				0.034
H15	1	2	2	2	1		0.013						0.004			0.001				
H16	1	2	2	2	2					0.010	0.023				0.006		0.005			
H17	2	1	1	1	1	0.094	0.021	0.099	0.108	0.011		0.070		0.010	0.021	0.087	0.028	0.159	0.091	0.091
H18	2	1	1	1	2		0.006		0.008	0.005			0.015	0.009	0.008	0.008			0.061	0.015
H19	2	1	1	2	1	0.004												0.005	0.052	0.053
H20	2	1	1	2	2															0.004
H21	2	1	2	1	1	0.347	0.319	0.238	0.205	0.156	0.352	0.206	0.204	0.195	0.206	0.297	0.320	0.179	0.198	0.095
H22	2	1	2	1	2		0.008	0.036	0.035		0.008	0.014	0.061	0.036	0.017	0.051	0.034	0.042	0.028	0.036
H23	2	1	2	2	1	0.040	0.014	0.045	0.058	0.045	0.007	0.104	0.066	0.046	0.064	0.013	0.027	0.051	0.036	0.011
H24	2	1	2	2	2	0.009	0.012	0.030		0.019	0.051	0.042	0.089	0.063	0.104		0.018	0.023		0.075
H25	2	2	1	1	1											0.009			0.010	0.060
H26	2	2	1	2	1															0.005
H27	2	2	1	2	2							0.005								0.007
H28	2	2	2	1	1			0.006		0.043			0.020		0.005	0.032	0.035			
H29	2	2	2	2	1												0.011			0.003
H30	2	2	2	2	2											0.017				

*^*a*^The data of five POALINs in Han-Yunnan were published previously (Shi et al., 2014).*

*^*b*^The data of five POALINs in Japanese and Caucasians were published previously (Kulski et al., 2010, 2011).*

The two most predominant haplotypes in all 15 populations were H5 (AluDPB2^∗^1: AluDQA2^∗^1: AluDQA1^∗^2: AluDRB1^∗^1: AluORF10^∗^1) and H21 (AluDPB2^∗^2: AluDQA2^∗^1: AluDQA1^∗^2: AluDRB1^∗^1: AluORF10^∗^1), both with the AluDQA1 insertion. Haplotype H6 (AluDPB2^∗^1: AluDQA2^∗^1: AluDQA1^∗^2: AluDRB1^∗^1: AluORF10^∗^2) differentiated the Maonan from the other populations (*P* < 0.01 after Bonferroni’s correction), whereas haplotype H7 (AluDPB2^∗^1: AluDQA2^∗^1: AluDQA1^∗^2: AluDRB1^∗^2: AluORF10^∗^1) differentiated the Caucasians from the other populations except for the Tu and Han-Yunnan (*P* < 0.01 after Bonferroni’s correction). Also, haplotype H8 (AluDPB2^∗^1: AluDQA2^∗^1: AluDQA1^∗^2: AluDRB1^∗^2: AluORF10^∗^2) differentiated the Caucasians from the other populations except from the Tu (*P* < 0.01 after Bonferroni’s correction). The haplotype H18 (AluDPB2^∗^2: AluDQA2^∗^1: AluDQA1^∗^1: AluDRB1^∗^2: AluORF10^∗^2) frequency was different between the Japanese and other populations but not from the Jinuo, Dai and Maonan (*P* < 0.01 after Bonferroni’s correction). On the other hand, haplotype H19 (AluDPB2^∗^2: AluDQA2^∗^1: AluDQA1^∗^1: AluDRB1^∗^2: AluORF10^∗^1) was observed only in four populations, with a significant difference obtained between Hani/Han-Yunnan and Japanese/Caucasians (*P* < 0.01 after Bonferroni’s correction).

### LD Analysis and Percentage Haplotypic Association Between POALINs and HLA Alleles

**FIGURE 2:**
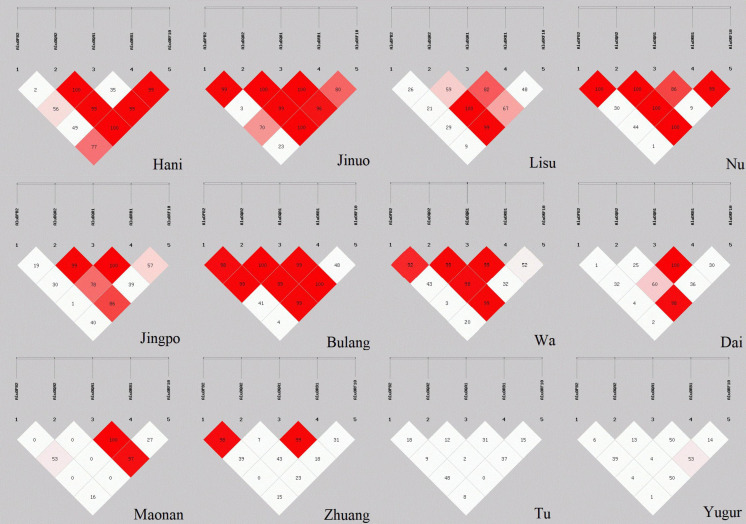
LD estimations (D′) among five POALINs within MHC II region for 12 Chinese ethnic populations.

D′ values for global LD between the five POALINs were calculated in twelve ethnic populations and are shown in Figure 2. LD values between the Alu loci were variable between the ethnic populations ranging from the absence of strong LD (D′ < 54%) between any of the Alu in the Yugur and Tu Mongolian populations to a strong LD (D′ > 0.8) between four or five Alu in the Jinuo, Nu, Bulang and Wa. The Hani, Lisu and Jingpo had strong LD (D′ > 0.8) between two or three Alu insertions, whereas the Dai, Maonan and Zhuang of the ancient Baiyue tribe and the Daic subfamily language had only two Alu in strong LD.

Supplementary Table 6 shows the frequency of HLA-DRB1 alleles and class II POALINS and that the percentage associations between these POALIN and particular HLA-DRB1 alleles were at very high (80–100%), high (>50–79%), moderate (20–50%) and low (<20%) percentages. For example, all of the 19 HLA-DRB1 alleles in the Hani were associated with four of five of the Alu insertions at high to very high percentages: 16 alleles (except for HLA-DRB1^∗^01:01, -DRB1^∗^08:03 and -DRB1^∗^10:01) associated at 67.3–100% with AluDQA1, 8 alleles (HLA-DRB1^∗^01:01, -DRB1^∗^04:03, -DRB1^∗^08:03, -DRB1^∗^09:01, -DRB1^∗^12:01, -DRB1^∗^14:01, -DRB1^∗^14:04 and -DRB1^∗^16:02) associated at 54.9–100% with AluDPB2, 4 alleles (HLA-DRB1^∗^01:01, -DRB1^∗^08:01, -DRB1^∗^15:02 and -DRB1^∗^15:04) associated at 57.1–100% with AluDRB1, and there was 100% association between AluDQA2 and HLA-DRB1^∗^10:01, but at very low frequency (0.00336). The AluORF10 was associated with the Hani HLA-DRB1 alleles only at low to moderate levels.

Supplementary Table 7 shows a summary of the comparative percentage association between HLA-DRB1 alleles and the Alu class II POALINs in 12 ethnic populations (this study), Chinese Han in Yunnan (Shi et al., 2014), Japanese and Caucasians (Kulski et al., 2010) from previous studies. Overall, there was a strong similarity of haplotypic associations between AluDQA1 and HLA-DRB1 alleles in all fifteen populations.

Table 5 shows a summary of the percentage association between HLA-DRB1 alleles and AluDRB1. Overall, all the populations except for the Hani and the Dai have 83 to 100% association between the AluDRB1 insertion and HLA-DRB1^∗^15 and HLA-DRB1^∗^16. In comparison, the AluDRB1 insertion was linked to six of six homozygous cell lines with HLA-DRB1^∗^01, seven of seven cell lines with -DRB1^∗^16, 10 of 11 cell lines with -DRB1^∗^15 and to none of the other 66 cell lines with nine other DRB1 lineage alleles (Supplementary Table 2). For the other Alu insertions, HLA-DRB1^∗^09 was not found in the Wa, but it had a moderate to very strong association (51–100%) with AluDPB2 in thirteen populations and a low association (31.7%) in the Lisu. For a comparison of the haplotypic associations with actual genomic sequence linkages, Supplementary Table 2 shows the percentage linkage between these five POALIN with HLA-DRB1 alleles detected in the MHC class II haplotype sequences of 90 homozygous cell-lines (Kulski et al., 2021). Because of ancestral recombination at sites between various Alu loci and the DRB1 allelic loci, the linkages detected in the cell lines were not present in all the different Chinese ethnic populations, although the general trends are maintained between and within populations.

**TABLE 5:** Percentage association between HLA-DRB1 alleles and AluDRB1.

DRB1 allele	Average of 10 ethnic groups^a^	Hani	Dai	Han-Yunnan^b^	Japanese^c^	Caucasian^c^
DRB1*01	64	68		100	75	100
DRB1*03	25		59	3		
DRB1*04	8		20	9	2	5
DRB1*07	11	50				
DRB1*08	10		50	6	5	
DRB1*09			13	3		
DRB1*10			100			50
DRB1*11			17			
DRB1*12	2		29	4		
DRB1*13	20		41	5		
DRB1*14	4	13	53	9		
DRB1*15	95	54	49	86	83	100
DRB1*16	94	50	26	87	100	100

*^*a*^10 ethnic groups include Jinuo, Lisu, Nu, Jingpo, Bulang, Wa, Maonan, Zhuang, Tu, and Yugur.*

*^*b*^The data in Han-Yunnan were published previously (Shi et al., 2014).*

*^*c*^The data in Japanese and Caucasian were published previously (Kulski et al., 2010, 2011).*

### Phylogenetic Trees and PCA Plots

**FIGURE 3:**
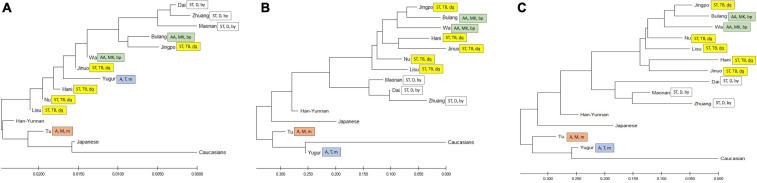
Neighbor-joining trees. **(A)** Neighbor-joining tree based on DA genetic distance from five POALIN allele frequencies. **(B)** Neighbor-joining tree based on HLA-DRB1 allele frequencies. **(C)** Neighbor-joining tree based on the DRB1/AluDRB1 haplotype frequencies. The colored labeled boxes represent the ancient tribe, language family and subfamily for each population listed in Table 1. Yellow represent Di-Qiang, Sino-Tibetan, Tibeto-Burman. Green represent Baipu, Austo-Asiatic, Mon-Khmer. White represent Baiyue, Sino-Tibetan, Daic. Orange represent Mongolian, Altaic, Mongolian. Blue represent Mongolian, Altaic, Tujue.

**FIGURE 4:**
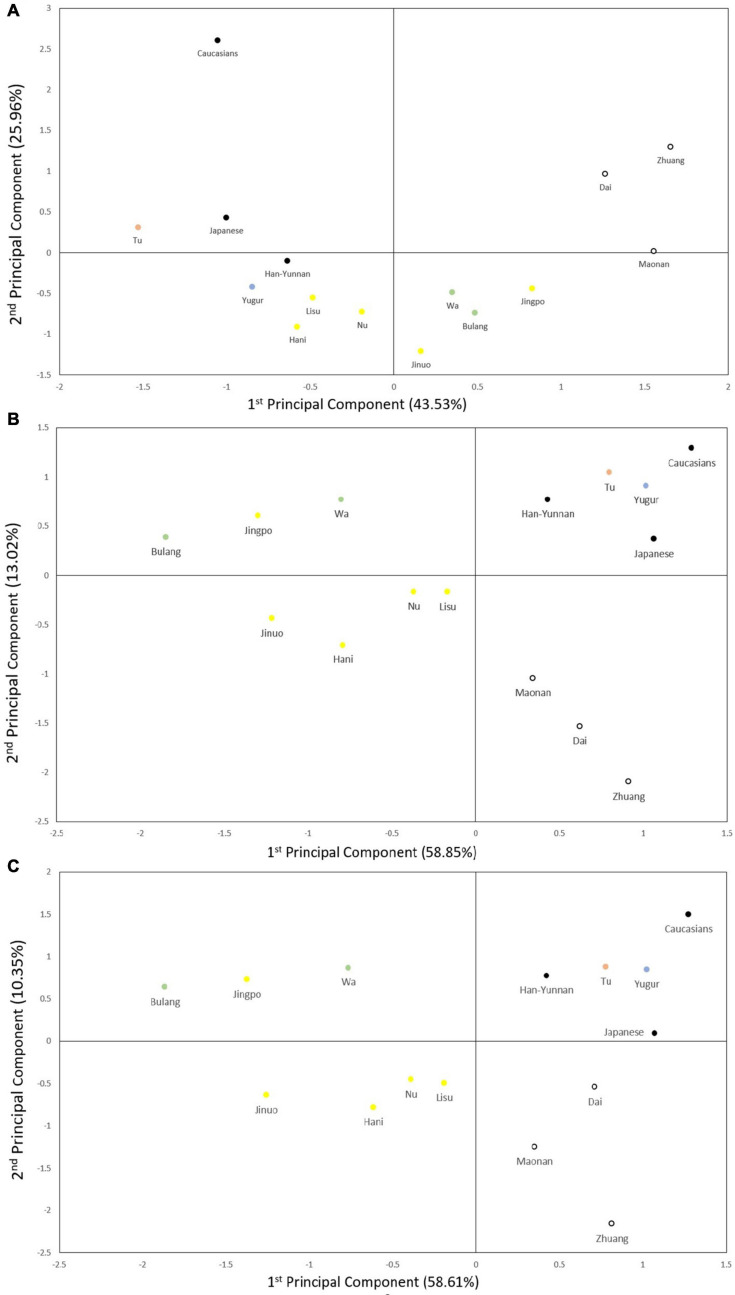
Principal component analysis (PCA). **(A)** PCA based on five POALIN allele frequencies. Contributions of the first and second components were 43.53% and 25.96%, respectively. **(B)** PCA based on HLA-DRB1 allele frequencies. Contributions of first and second components were 58.85% and 13.02%. **(C)** PCA based on the DRB1/AluDRB1 haplotype frequencies. Contributions of first and second components were 58.61% and 10.35%. The colored dots represent the ancient tribe, language family and subfamily for each population listed in Table 1. Yellow represent Di-Qiang, Sino-Tibetan, Tibeto-Burman. Green represent Baipu, Austo-Asiatic, Mon-Khmer. White represent Baiyue, Sino-Tibetan, Daic. Orange represent Mongolian, Altaic, Mongolian. Blue represent Mongolian, Altaic, Tujue.

To compare the diversity of these ethnic populations, we constructed phylogenetic trees (Figure 3) and PCA plots (Figure 4) based on POALIN alleles, HLA-DRB1 alleles and DRB1-AluDRB1 haplotype frequencies. The topology for the NJ tree constructed using the DA of POALIN alleles (Figure 3A), revealed two distinct clusters: (1) the Dai, Zhuang and Maonan of the Daic subfamily in the Sino-Tibetan language family, and (2) the Jingpo of the Tibeto-Burman subfamily in the Sino-Tibetan language family with the Bulang stemming from the Wa, which are both part of the Mon-Khmer subfamily in the Austo-Asiatic language family. A third cluster was the stepwise grouping of Lisu, Nu, Hani and Jinuo of the Tibeto-Burman with the Mongolian Yugur of the Tujue subfamily in the Altaic language family inserted between the Hani and the Jinuo. The Han from Yunnan province grouped at the lower extremity of the 12 Chinese minority ethnic groups and away from the Japanese and the Caucasians that had grouped at the opposite end of the tree to that of the Daic cluster.

The topology of the NJ trees based on HLA-DRB1 allele frequencies and DRB1/AluDRB1 haplotypes were similar to each other (Figures 3B,C) and both revealed two distinct clusters: (1) the Dai, Zhuang and Maonan of the Daic subfamily in the Sino-Tibetan language family, and (2) the Bulang and Wa of the Mon-Khmer subfamily in the Austo-Asiatic language family separated from the Jingpo, Hani, Jinuo, Nu and Lisu group of the Tibeto-Burman subfamily in the Sino-Tibetan language family. The Han-Yunnan population grouped between the Chinese minority populations and the Japanese and at a genetic distance away from the Mongolian Tu and Yugur and the Caucasians. In this regard, the POALIN and HLA-DRB1 allele frequencies both grouped the 13 Chinese ethnic populations into their respective subfamilies and language families. The main exception was that the POALIN frequencies separated the Tu and Yugur at a greater distance from each other (Figure 3A), whereas the HLA-DRB1 allele frequencies placed them more closely together between the Japanese and the Caucasians (Figures 3B,C).

The PCA plots for the POALIN alleles (Figure 4A), HLA-DRB1 alleles (Figure 4B) and DRB1-AluDRB1 haplotypes (Figure 4C) showed that the distinct linguistic clusters of the 15 populations in each of four quadrants are similar to those revealed by the NJ trees (Figure 3). These plots have placed the Jingpo closer to the Mon-Khmer subfamily than to the Tibeto-Burman subfamily from which the Jingpo are believed to have originated, and the genetic distance between the Mongolian Tu and Yugur is greater for the POALIN alleles than the HLA-DRB1 alleles and DRB1-AluDRB1 haplotypes. Also, the Caucasians are the genetic outgroup in relation to the 13 Chinese ethnic populations and the Japanese.

## Discussion

In this study, we examined the genetic variations of the five POALIN and HLA-DRB1 allele and haplotype frequencies to further elucidate the association between the MHC class II POALIN and the classical HLA-DRB1 allele frequencies in 12 Chinese minority populations. The HLA-DRB1 alleles are used widely and commonly for assessing the genetic structure and differences within and between different populations (Di and Sanchez-Mazas, 2011; Sun et al., 2015; Weiskopf et al., 2016; Gonzalez-Galarza et al., 2020). The frequency of the HLA-DRB1 alleles within the 12 Chinese minority populations were similar to previous reports (Ogata et al., 2007; Shi et al., 2008, 2010b, 2011; Sun et al., 2015; Tao et al., 2020). On the other hand, the previous studies on the distribution and frequency of the MHC class II POALIN dimorphisms were limited to only three populations, the Caucasian, Japanese (Kulski et al., 2010), and Chinese Han in Yunnan (Shi et al., 2014), and this published data provided the three outlying comparative populations for the present study. Therefore, we have provided new data on the POALIN frequencies for 12 Chinese minority populations that were selected for genetic analysis because of their culture, known ancient history and connection to five distinct language subfamilies, the Tibeto-Burman, Mon-Khmer, Daic, Mongolian and Tujue (Table 1).

Phylogenetic trees and PCA (Figures 3, 4) show that the Alu insertion dimorphism, HLA-DRB1 alleles and the DRB1-AluDRB1 haplotype diversity are associated strongly with the population characteristics of language family, migration and sociality. The Daic family, including the Dai, Zhuang and Maonan, always clustered closely together based on the POALIN dimorphisms, HLA-DRB1 alleles and HLA DRB1-AluDRB1 haplotypes. The Tibeto-Burman subfamily of the Jinuo, Hani, Lisu and Nu have certain shared population characteristics due to their migration from the north, and therefore are genetically closer to the Yugur and Tu northern populations, which belong to Mongolian tribal family. Surprisingly, the Jingpo from Tibeto-Burman subfamily are genetically closer to the Mon-Khmer family (Bulang and Wa) than to other populations from Tibeto-Burman subfamily probably because these three populations have long lived closely together in the mountains of the western part of Yunnan and have been infected by similar pathogens from the infectious environment. For example, malaria is a serious infectious disease prevalent in China since 2700 BC, and Yunnan Province is a high incidence area of malaria, especially in the border area between China and Myanmar (Cox, 2010; Bi et al., 2013; Diouf et al., 2014). Similarly, the Jinuo and Bulang who live closely together within this same area, also may have undergone high selective pressure from malaria.

The five different POALIN dimorphic frequencies provide unique evolutionary and genetic information on the relationships between the 12 Chinese minority populations. The frequencies of AluDPB2, AluDQA2 and AluDQA1 in the Jingpo had significant differences with the other four populations (*P* < 0.01 after Bonferroni’s correction) of the Tibeto-Burman subfamily. This suggests an expansion of these Alu insertions in the Jingpo people as a consequence of their different population histories or environmental effects. In comparison, the Bulang, a member of the Mon-Khmer family, had the highest POALIN frequency (0.995) for AluDQA1 in all 15 populations. This is the highest and closest to subpopulation genetic fixation for any of the MHC POALIN frequencies in world populations suggesting substantial long term population isolation. The frequencies of AluORF10 were higher in Dai, Maonan, and Zhuang (Daic subfamily in the Sino-Tibetan language family) than in the other nine Chinese minority populations. AluDQA1 was the highest POALIN frequency (0.777 and 0.903, respectively) in the Tu and the Yugur with a significant difference between these two populations (*P* < 0.01 after Bonferroni’s correction). HLA-DRB1^∗^09:01 had the strongest association (100%) with AluDQA1, and was the highest frequency (0.127 and 0.134) in the Tu and Yugur, respectively. According to historical records, all the Altaic language speaking groups such as the Tu and the Yugur who speak the Mongolian, Tujue, or Manchu-Tungusic sub-languages originated from the people and places overrun by the Mongol Empire and from the border adjacent to Northeastern China in the 13th century (Guo, 2000; Chu et al., 2006). HLA-DRB1^∗^12:02 also had strong associations (88.7–100%) with AluDQA1 with a high frequency (0.160–0.550) in eight populations (Hani, Jinuo, Lisu, Nu, Jingpo, Bulang, Wa, and Maonan).

It is reported that the distribution of DRB1 allele frequencies for a Mongolian subpopulation in Yunnan was different to a Mongolian population of inner Mongolia and much closer to the Hani population of Yunnan (Sun et al., 2015). They hypothesized that the difference between the two Mongolian populations was due partly to gene flow and pathogen driven selection. We found a large differentiation between two Mongolian populations for the Alu alleles, but not for the HLA-DRB1 alleles. The Alu analysis placed the Mongolian Yugur within a cluster of the Di-Qiang subfamilies and at a substantial distance away from the Mongolian Tu, whereas the DRB1 allele frequencies for the two Mongolian populations placed them closer together at a genetic distance between the Japanese and Caucasians (Figures 3, 4). We attribute this difference between the two Mongolian populations for the Alu analysis mainly to a twofold difference in the AluDQA1^∗^1 frequencies (Table 3). However, it is possible that the frequencies of particular DRB1 alleles of the two distinct Mongolian populations may have placed them closer together because of pathogen driven selection at that particular individual gene in contrast to the more independent and possibly less effective Alu loci. In this regard, the inheritance of identical by descent or identical by state genomic loci and/or haplotypes may in part be driven by selection, gene flow and various social and geographic factors, but has yet to be defined and investigated using a greater variety of different genomic markers for comparative analyses.

Overall, the branching patterns of the interrelationships between the populations and population clusters were similar for the Alu and DRB1 allelic frequencies, although the genetic distances between particular populations were substantially different. Most of these similarities are likely due to the haplotypic characteristics between the Alu dimorphism and the DRB1 alleles (Kulski et al., 2010, 2021), as exemplified in this study with a comparison between the NJ trees of the HLA-DRB1 alleles and HLA-DRB1-AluDRB1 haplotypes (Figure 3). It is clear from this and previous studies that the closer the dimorphic Alu is to the HLA-DRB1 locus the stronger the haplotypic linkage/association and recombination resistance (Kulski et al., 2010, 2011, 2021). This seems to be the case for AluDRB1 that is most strongly associated with HLA-DRB1^∗^15 and -DRB1^∗^16 (Table 5) and is located within 14 kb of the HLA-DRB1 locus. In contrast, AluORF6 and AluDP2, which are 233 kb and 536 kb, respectively, from the HLA-DRB1 locus (Supplementary Figure 1), are associated with many different DRB1 alleles possibly because many more recombination events had occurred between their loci. The five genotyped and haplotyped ‘lineage by descent,’ dimorphic Alu described in this study provide clues to the diversity of the MHC class II region of the 12 Chinese minority populations. However, further studies using fully phased genomic sequences of the MHC class II region within these historically small ethnic communities that are still strongly linked together by ancestry, culture and language might provide a better understanding of these POALIN haplotypic associations within the context of human MHC class II diversity, identity by descent (and/or by chance or state), haplotype shuffling and ancestral recombinations (Dawkins et al., 1999; Alper et al., 2006; Larsen et al., 2014).

The POALINs in the current study are all members of the young Alu subfamily, with AluDQA1 and AluDRB1 belonging to the AluY subgroup and AluDQA2, AluDPB2 and AluORF10 belonging to the youngest AluYa5 or AluYb8 subgroup (Kulski et al., 2010). AluDQA1 appears to be the oldest of the five POALINs on the basis of having the highest POALIN frequency in the 15 populations (Table 3) and its association with most of the HLA-DRB1 supertypes (Supplementary Table 7). Thus, the AluDQA1 insertion was distributed widely in the Chinese ethnic populations and associated strongly as a haplotype with all or most of the HLA-DRB1 alleles. The frequency of AluDQA2 was higher in the Caucasians than in the Chinese populations or Japanese. The hypothesis that AluDQA2 may have originated in Caucasians (Kulski et al., 2010; Shi et al., 2014) is confirmed by the present study.

The frequencies of AluDRB1 were the highest in the Dai, Maonan, Zhuang, which belong to Tibeto-Burman language subfamily. The AluDRB1 with the frequency range from 0.10–0.455 had a strong association with HLA-DRB1^∗^15 and -DRB1^∗^16 in most populations. However, there was a significantly lower % association of <55% between the AluDRB1 insertion and HLA-DRB1^∗^15 or HLA-DRB1^∗^16 in Hani and Dai compared to the other 11 Chinese ethnic groups including the Han-Yunnan, and the Japanese and Caucasians (Table 5). This could be due to primer mutation with allelic dropout, an AluDRB1 deletion, recombination events, or a high level of interbreeding among members of the population with the HLA-DRB1^∗^15 or HLA-DRB1^∗^16 haplotype that was missing the AluDRB1 insertion in the founding group. By comparison, the AluDRB1 insertion is very much limited by linkage (or association) to the HLA-DRB1^∗^01, -DRB1^∗^10 (DR1 supertypes), -DRB1^∗^15, and the -DRB1^∗^16 (DR51 supertypes) allelic lineages, which occurred after their separation from the DR8, DR52 and DR53 supertypes (Kulski et al., 2010). On this basis, the AluDQA1 insertion must have happened much earlier than the AluDRB1 insertion during human evolution and population expansions. These results confirm that the AluDRB1 insertion probably originated in an ancestral HLA-DRB1 allele as a progenitor of the DR51 supertypes (Kulski et al., 2010), which contained HLA-DRB1^∗^15 and -DRB1^∗^16 (Andersson, 1998; Gibbons et al., 2004).

AluDPB2 has a frequency range from 0.278 to 0.574 in fifteen populations, with low- to high-level percentage associations with many different HLA-DRB1 alleles (Supplementary Table 7). This greater number of associations between AluDPB2 and HLA-DRB1 than between AluDRB1 and HLA-DRB1 is probably because the AluDPB2 locus is 536 kb from the HLA-DRB1 locus with the likelihood of numerous ancient recombination events occurring in between the two loci (Supplementary Figure 1; Kulski et al., 2021). The AluORF10 had a strong association with HLA-DRB1^∗^15 only in Caucasians (89.1%). In contrast, the AluORF10 was associated strongly with HLA-DRB1^∗^16 in eight East Asian populations (Jingpo, Wa, Maonan, Zhuang, Tu, Yugur, Han-Yunnan, and Japanese); whereas HLA-DRB1^∗^16 was absent in the Caucasian population. This suggests at least one or more recombination events at an unidentified junction between the AluORF10 and HLA-DRB1 locations in the ancestral progenitors of the DR51 supertypes.

Although this study focused on Alu and HLA-DRB1 evolutionary genetic markers and population structure and was not related directly to medical or health issues, it is noteworthy that the Alu indels could have enhancer and other regulatory roles that affect the expression of HLA class II genes and/or other genes in the MHC and elsewhere in the human genome (Hasler and Strub, 2006; Moolhuijzen et al., 2010; Spirito et al., 2019; Goubert et al., 2020; Kulski et al., 2021). Many Alu elements of the AluJ, AluS, and AluY subfamilies are transcriptionally active with highly expressed self-cleaving ribozyme activity during T-cell activation and thermal and endoplasmic reticulum stress (Hernandez et al., 2020). Furthermore, Wang et al. (2017) identified two Alu indels Alu-5072 and Alu-5075 in the class II region as potential enhancers for HLA-DRB5, and HLA-DQB1-AS1 associated with phenotypes of lymphoma, Hodgkin lymphoma and chronic hepatitis B infection, respectively (Wang et al., 2017). In this regard, Alu-5057 is probably the AluDRB1 indel at the 5′ end of HLA-DRB1 (Supplementary Figure 1). Thus, the question remains whether the other four Alu indels described in this study also have enhancer functions as Wang et al. (2017) reported for Alu-5072 and Alu-5075 (Wang et al., 2017). On the basis of these published findings, the transcriptional activity and role of Alu in the human MHC during epigenetic regulation needs to be investigated and better defined. Also, the Alu indels both as genotypes and haplotypes within the MHC could have important functions in cancer, autoimmunity and immunity to infections that have yet to be addressed and investigated.

We used a set of five-locus POALINs from the MHC class I region as lineage markers in a previous study to determine the haplotypic association and differentiation of MHC class I polymorphic Alu insertions and HLA-B/Cw alleles in seven Chinese ethnic populations (Yao et al., 2010). The POALIN markers that we used in this study were limited to five loci in the MHC class II region, but were a sufficient number to effectively micro-differentiate between 15 populations. The advantages of these POALIN lineage markers within the MHC class I and class II regions are their applicability – they are well defined, cheap to prepare and administer in the laboratory, and they produce results that are reasonably easy to interpret. In future work, the more widely studied MHC class I 5-loci POALINs (Yao et al., 2010; Abeid et al., 2019; Kulski et al., 2019), other autosomal Alu loci (Antunez-de-Mayolo et al., 2002), and STR loci (Garcia-Obregon et al., 2011) could be included in the haplotype analyses to broaden the genetic distances and diversity between and within the various populations.

In conclusion, the unique finding in this study, not previously reported, is that the MHC class II POALIN and HLA-DRB1 allele frequencies both grouped the 12 Chinese minority ethnic populations into their respective subfamilies and language families. When compared with the previously reported data of the Chinese Han in Yunnan, Japanese and Caucasians, it is evident that the POALINs in MHC class II, like the polymorphic class I and class II HLA genes, are informative genetic and haplotype markers, which can be used cheaply and simply in studies of population diversity, forensic medicine and disease research.

## Data Availability Statement

The raw data supporting the conclusions of this article will be made available by the authors, without undue reservation.

## Author Contributions

LiS and YY conceived and designed the research. YC, SL, JY, YT, and XZ performed the experiments. YC and JK analyzed the data. SL and YT collected the samples. YC, YY, LiS, and JK wrote and revised the manuscript. All authors read and approved the final version of the manuscript. All authors contributed to the article and approved the submitted version.

## Conflict of Interest

The authors declare that the research was conducted in the absence of any commercial or financial relationships that could be construed as a potential conflict of interest.

## Footnotes

1 https://www.ncbi.nlm.nih.gov/bioproject/

2 http://www.repeatmasker.org/cgi-bin/WEBRepeatMasker

3 http://analysis.bio-x.cn/myAnalysis.php

## Supplementary Material

Supplementary Figure S1 Map of the location of the five POALIN within the MHC class II region. **(a)** Positions of the Alu indels AluORF10, AluDRB1, AluDQB1, AluDQA2, and AluDPB2 on the top horizontal line relative to the positions of the HLA class II genes. The horizontal arrows indicate 5′ to 3′ coding direction. **(b)** Magnification of the genomic location between the HLA-DRB1 and HLA-DQA1 genes to indicate the relative positions of the AluDRB1 and AluDQA1 insertions that are located ∼13.7 kb and 36.1 kb from the 5′ end of HLA-DRB1, respectively. It is a computer image taken from the online UCSC browser at https://genome.ucsc.edu/cgi-bin/hgGateway for chr6:32,565,856–32,632,194 covering 66,339 bp and including representations of Curated RefSeq, gene expression profile from GTEx RNA-seq, ENCODE cCREs, and Repeat Elements by RepeatMasker and labeled to show the positions of the AluDRB1 and AluDQA1 insertions along the SINE row.

Supplementary Figure S2 The Electrophoresis results for different sized PCR products of Five MHC POALINs. The POALIN alleles are dimorphic structures whereby the absence of the Alu insertion at the Alu locus is the Alu^∗^1 allele and the presence of the Alu insertion is the Alu^∗^2 allele. 1: the PCR products of AluORF10 1, 1; 2: the PCR products of AluORF10 1, 2; 3: the PCR products of AluORF10 2, 2; 4: the PCR products of AluDQA1 2, 2; 5: the PCR products of AluDQA1 1, 2; 6: the PCR products of AluDQA1 1, 1; 7: the PCR products of AluDPB2 2, 2; 8: the PCR products of AluDPB2 1, 2; 9: the PCR products of AluDPB2 1, 1; 10: the PCR products of AluDRB1 1, 1; 11: the PCR products of AluDRB1 1, 2; 12: the PCR products of AluDRB1 2, 2; 13: the PCR products of AluDQA2 1, 1; 14: the PCR products of AluDQA2 1, 2; 15: the PCR products of AluDQA2 2, 2; M1: 1000bp Marker; M2: 2000bp Marker.
