# Supplementary figures and images for: Haplotypic Associations and Differentiation of MHC Class II Polymorphic Alu Insertions at Five Loci With HLA-DRB1 Alleles in 12 Minority Ethnic Populations in China
Source: Front Genet. 2021 Jul 7;12:636236. doi: 10.3389/fgene.2021.636236 (PMC8292818; doi:10.3389/fgene.2021.636236)

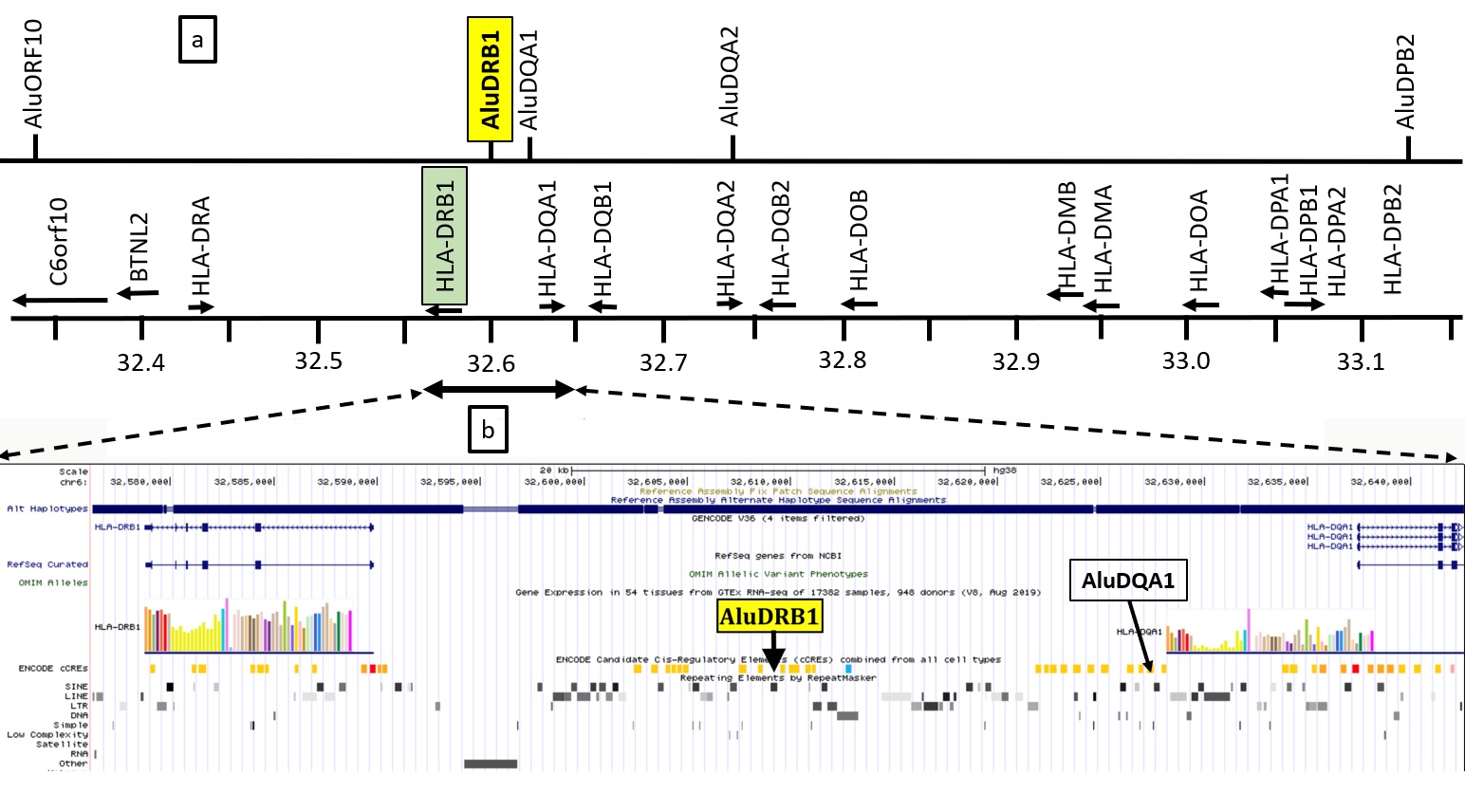

Supplement: Supplementary Figure S1 — Map of the location of the five POALIN within the MHC class II region. (a) Positions of the Alu indels AluORF10, AluDRB1, AluDQB1, AluDQA2, and AluDPB2 on the top horizontal line relative to the positions of the HLA class II genes. The horizontal arrows indicate 5′ to 3′ coding direction. (b) Magnification of the genomic location between the HLA-DRB1 and HLA-DQA1 genes to indicate the relative positions of the AluDRB1 and AluDQA1 insertions that are located ∼13.7 kb and 36.1 kb from the 5′ end of HLA-DRB1, respectively. It is a computer image taken from the online UCSC browser at https://genome.ucsc.edu/cgi-bin/hgGateway for chr6:32,565,856–32,632,194 covering 66,339 bp and including representations of Curated RefSeq, gene expression profile from GTEx RNA-seq, ENCODE cCREs, and Repeat Elements by RepeatMasker and labeled to show the positions of the AluDRB1 and AluDQA1 insertions along the SINE row. [file Image_1.JPEG]

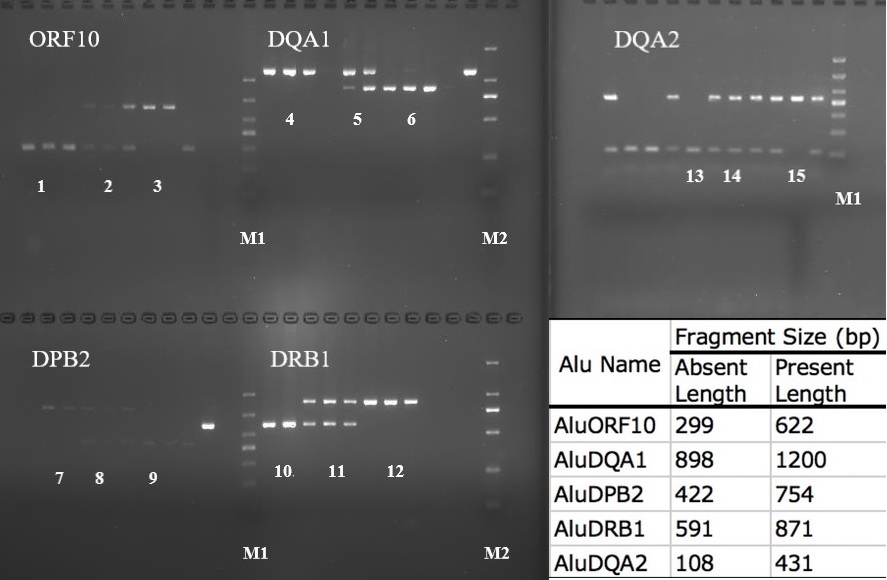

Supplement: Supplementary Figure S2 — The Electrophoresis results for different sized PCR products of Five MHC POALINs. The POALIN alleles are dimorphic structures whereby the absence of the Alu insertion at the Alu locus is the Alu∗1 allele and the presence of the Alu insertion is the Alu∗2 allele. 1: the PCR products of AluORF10 1, 1; 2: the PCR products of AluORF10 1, 2; 3: the PCR products of AluORF10 2, 2; 4: the PCR products of AluDQA1 2, 2; 5: the PCR products of AluDQA1 1, 2; 6: the PCR products of AluDQA1 1, 1; 7: the PCR products of AluDPB2 2, 2; 8: the PCR products of AluDPB2 1, 2; 9: the PCR products of AluDPB2 1, 1; 10: the PCR products of AluDRB1 1, 1; 11: the PCR products of AluDRB1 1, 2; 12: the PCR products of AluDRB1 2, 2; 13: the PCR products of AluDQA2 1, 1; 14: the PCR products of AluDQA2 1, 2; 15: the PCR products of AluDQA2 2, 2; M1: 1000bp Marker; M2: 2000bp Marker. [file Image_2.JPEG]
