# Supplementary material for: Haplotypic Associations and Differentiation of MHC Class II Polymorphic Alu Insertions at Five Loci With HLA-DRB1 Alleles in 12 Minority Ethnic Populations in China
Source: Front Genet. 2021 Jul 7;12:636236. doi: 10.3389/fgene.2021.636236 (PMC8292818; doi:10.3389/fgene.2021.636236)
Supplement: Supplementary file 3 [file Data_Sheet_1.docx]

**Supplementary Table 1. Primer sequences, amplicon product sizes and PCR conditions for the DNA ampliﬁcation of ﬁve MHC class II POALIN loci**

| Alu Name |  | Primer Sequence (5'-3') | Fragment Size (bp) | | Annealing Tm(◦C) | References |
| --- | --- | --- | --- | --- | --- | --- |
|  |  |  | Absent Length | Present Length |  |  |
| AluDPB2 | Forward | AGACTAAGGAGTGTGGATTTCTC | 422 | 754 | 60 | J. K. Kulski *et al* (2010) |
| AluDPB2 | Reverse | ACTTCTATCCTCCTCTTTCCTC | 422 | 754 | 60 | J. K. Kulski *et al* (2010) |
| AluDQA2 | Forward | TAGTCCCTTGCAATATCTGAAATC | 108 | 431 | 61 | This study |
| AluDQA2 | Reverse | AAGGAGAATTGCTTTAACTTAGAA | 108 | 431 | 61 | This study |
| AluDQA1 | Forward | AAGAGCACTACGCAGGTTCACACT | 898 | 1200 | 61 | This study |
| AluDQA1 | Reverse | GAATTCAAGCCCATGCAGTCTAAC | 898 | 1200 | 61 | This study |
| AluDRB1 | Forward | CACTAGTCAGTTCATCCTCTTGT | 591 | 871 | 60 | J. K. Kulski *et al* (2010) |
| AluDRB1 | Reverse | AAATGCTTCCTTCTTGTTGTAAAT | 591 | 871 | 60 | Lei Shi *et al* (2014) |
| AluORF10 | Forward | ACAGTCCAGTGAATTGCCTATGAGAA | 299 | 622 | 55 | Lei Shi *et al* (2014) |
| AluORF10 | Reverse | TAGGGAGTAAGGAGAGGTGGAAGGA | 299 | 622 | 55 | Lei Shi *et al* (2014) |

**Supplementary Table 2. Linkage between five POALIN and HLA-DRB1 allelic lineages in 90 homozygous cell lines (Norman et al 2017)***

| MHC Class II POALIN | DRB1 Allele Linkage | No. Haplotypes | Distance between Loci |
| --- | --- | --- | --- |
|  | 90 Haplotypes | (% of gene allele) | kb |
| AluORF10 (n, 13) | DRB1*15 | 6 of 11 (54.6%) | 232.5 kb |
| AluORF10 (n, 13) | DRB1*01 | 2 of 6 (33.3%) |  |
| AluORF10 (n, 13) | DRB1*07 | 3 of 9 (33.3%) |  |
| AluORF10 (n, 13) | DRB1*16 | 1 of 7 (14.3%) |  |
| AluORF10 (n, 13) | DRB1*04 | 1 of 13 (7.7%) |  |
| AluORF10 (n, 13) | DRB1*03 | 0 of 11 (0%) |  |
| AluORF10 (n, 13) | DRB1*13 | 0 of 8 (0%) |  |
| AluORF10 (n, 13) | DRB1*11 | 0 of 9 (0%) |  |
| AluORF10 (n, 13) | DRB1*14 | 0 of 6 (0%) |  |
| AluORF10 (n, 13) | DRB1*08 | 0 of 7 (0%) |  |
| AluORF10 (n, 13) | DRB1*09 | 0 of 2 (0%) |  |
| AluORF10 (n, 13) | DRB1*12 | 0 of 1 (0%) |  |
|  |  |  |  |
| AluDRB1 (n, 23) | DRB1*01 | 6 of 6 (100%) | 13.7 kb |
| AluDRB1 (n, 23) | DRB1*16 | 7 of 7 (100%) |  |
| AluDRB1 (n, 23) | DRB1*15 | 10 of 11 (90.9%) |  |
| AluDRB1 (n, 23) | DRB1*03 | 0 of 11 (0%) |  |
| AluDRB1 (n, 23) | DRB1*13 | 0 of 8 (0%) |  |
| AluDRB1 (n, 23) | DRB1*11 | 0 of 9 (0%) |  |
| AluDRB1 (n, 23) | DRB1*14 | 0 of 6 (0%) |  |
| AluDRB1 (n, 23) | DRB1*07 | 0 of 9 (0%) |  |
| AluDRB1 (n, 23) | DRB1*08 | 0 of 7 (0%) |  |
| AluDRB1 (n, 23) | DRB1*04 | 0 of 13 (0%) |  |
| AluDRB1 (n, 23) | DRB1*09 | 0 of 2 (0%) |  |
| AluDRB1 (n, 23) | DRB1*12 | 0 of 1 (0%) |  |
|  |  |  |  |
| AluDQA1 (n, 47) | DRB1*07 | 9 of 9 (100%) | 36.1 kb |
| AluDQA1 (n, 47) | DRB1*04 | 13 of 13 (100%) |  |
| AluDQA1 (n, 47) | DRB1*09 | 2 of 2 (100%) |  |
| AluDQA1 (n, 47) | DRB1*15 | 10 of 11 (90.9%) |  |
| AluDQA1 (n, 47) | DRB1*16 | 6 of 7 (85.7%) |  |
| AluDQA1 (n, 47) | DRB1*08 | 6 of 7 (85.7%) |  |
| AluDQA1 (n, 47) | DRB1*11 | 1 of 9 (11.1%) |  |
| AluDQA1 (n, 47) | DRB1*01 | 0 of 5 (0%) |  |
| AluDQA1 (n, 47) | DRB1*03 | 0 of 11 (0%) |  |
| AluDQA1 (n, 47) | DRB1*13 | 0 of 8 (0%) |  |
| AluDQA1 (n, 47) | DRB1*14 | 0 of 6 (0%) |  |
| AluDQA1 (n, 47) | DRB1*12 | 0 of 1 (0%) |  |
|  |  |  |  |
| AluDQA2 (n, 11) | DRB1*03 | 5 of 11 (45.5%) | 150.5 kb |
| AluDQA2 (n, 11) | DRB1*13 | 2 of 7 (28.6%) |  |
| AluDQA2 (n, 11) | DRB1*14 | 1 of 6 (16.7%) |  |
| AluDQA2 (n, 11) | DRB1*01 | 1 of 6 (16.7%) |  |
| AluDQA2 (n, 11) | DRB1*07 | 1 of 9 (11.1%) |  |
| AluDQA2 (n, 11) | DRB1*11 | 1 of 11 (9.1%) |  |
| AluDQA2 (n, 11) | DRB1*08 | 0 of 7 (0%) |  |
| AluDQA2 (n, 11) | DRB1*15 | 0 of 11 (0%) |  |
| AluDQA2 (n, 11) | DRB1*16 | 0 of 7 (0%) |  |
| AluDQA2 (n, 11) | DRB1*04 | 0 of 13 (0%) |  |
| AluDQA2 (n, 11) | DRB1*09 | 0 of 2 (0%) |  |
| AluDQA2 (n, 11) | DRB1*12 | 0 of 1 (0%) |  |
|  |  |  |  |
| AluDPB2 (n, 30) | DRB1*01 | 3 of 4 (75%) | 535.8 kb |
| AluDPB2 (n, 30) | DRB1*08 | 4 of 6 (66.7%) |  |
| AluDPB2 (n, 30) | DRB1*14 | 4 of 6 (66.7%) |  |
| AluDPB2 (n, 30) | DRB1*15 | 4 of 7 (57.1%) |  |
| AluDPB2 (n, 30) | DRB1*07 | 4 of 7 (57.1%) |  |
| AluDPB2 (n, 30) | DRB1*13 | 2 of 6 (33.3%) |  |
| AluDPB2 (n, 30) | DRB1*16 | 2 of 6 (33.3%) |  |
| AluDPB2 (n, 30) | DRB1*11 | 2 of 7 (28.6%) |  |
| AluDPB2 (n, 30) | DRB1*04 | 3 of 11 (27.3%) |  |
| AluDPB2 (n, 30) | DRB1*03 | 2 of 8 (25%) |  |
| AluDPB2 (n, 30) | DRB1*09 | 0 of 2 (0%) |  |
| AluDPB2 (n, 30) | DRB1*12 | 0 of 1 (0%) |  |

* Ref: Norman et al (2017). Sequences of 95 human MHC haplotypes reveal extreme variation in genes other than highly polymorphic HLA class I and II. Genome Research 27 813-823.

**Supplementary Table 3. The *P* values of five MHC POALINs allelic frequencies at five loci in 15 populations**

| *P* Value (Bonferroni correction) | | | | | | | | | | | | | | |  |
| --- | --- | --- | --- | --- | --- | --- | --- | --- | --- | --- | --- | --- | --- | --- | --- |
| DPB2 | | | | | | | | | | | | | | |  |
|  | Hani (n=149) | Jinuo (n=75) | Lisu (n=79) | Nu (n=82) | Jingpo (n=95) | Bulang (n=109) | Wa (n=109) | Dai (n=121) | Maonan (n=78) | Zhuang (n=101) | Tu (n=110) | Yugur (n=93) | Han-Yunnan (n=186) | Japanese (n=100) | |
| Jinuo (n=75) | 0.023 |  |  |  |  |  |  |  |  |  |  |  |  |  | |
| Lisu (n=79) | 0.445 | 0.178 |  |  |  |  |  |  |  |  |  |  |  |  | |
| Nu (n=82) | 0.105 | 0.531 | 0.457 |  |  |  |  |  |  |  |  |  |  |  | |
| Jingpo (n=95) | 0.000 | 0.048 | 0.001 | 0.007 |  |  |  |  |  |  |  |  |  |  | |
| Bulang (n=109) | 0.088 | 0.472 | 0.460 | 0.956 | 0.004 |  |  |  |  |  |  |  |  |  | |
| Wa (n=109) | 0.234 | 0.248 | 0.768 | 0.615 | 0.001 | 0.629 |  |  |  |  |  |  |  |  | |
| Dai (n=121) | 0.370 | 0.147 | 0.982 | 0.426 | 0.000 | 0.423 | 0.760 |  |  |  |  |  |  |  | |
| Maonan (n=78) | 0.006 | 0.703 | 0.081 | 0.307 | 0.111 | 0.254 | 0.114 | 0.059 |  |  |  |  |  |  | |
| Zhuang (n=101) | 0.137 | 0.388 | 0.570 | 0.830 | 0.002 | 0.863 | 0.763 | 0.543 | 0.200 |  |  |  |  |  | |
| Tu (n=110) | 0.647 | 0.011 | 0.266 | 0.055 | 0.000 | 0.044 | 0.125 | 0.204 | 0.003 | 0.071 |  |  |  |  | |
| Yugur (n=93) | 0.666 | 0.087 | 0.747 | 0.272 | 0.000 | 0.261 | 0.510 | 0.702 | 0.033 | 0.349 | 0.416 |  |  |  | |
| Han-Yunnan (n=186) | 0.387 | 0.097 | 0.933 | 0.334 | 0.000 | 0.319 | 0.649 | 0.901 | 0.033 | 0.435 | 0.204 | 0.764 |  |  | |
| Japanese (n=100) | 0.883 | 0.025 | 0.405 | 0.104 | 0.000 | 0.090 | 0.222 | 0.341 | 0.008 | 0.135 | 0.780 | 0.598 | 0.357 |  | |
| Caucasians (n=174) | 0.319 | 0.126 | 0.972 | 0.402 | 0.000 | 0.393 | 0.751 | 0.990 | 0.046 | 0.520 | 0.166 | 0.673 | 0.879 | 0.299 | |
| DQA2 | | | | | | | | | | | | | | |  |
|  | Hani (n=149) | Jinuo (n=75) | Lisu (n=79) | Nu (n=82) | Jingpo (n=95) | Bulang (n=109) | Wa (n=109) | Dai (n=121) | Maonan (n=78) | Zhuang (n=101) | Tu (n=110) | Yugur (n=93) | Han-Yunnan (n=186) | Japanese (n=100) | |
| Jinuo (n=75) | 0.222 |  |  |  |  |  |  |  |  |  |  |  |  |  | |
| Lisu (n=79) | 0.088 | 0.695 |  |  |  |  |  |  |  |  |  |  |  |  | |
| Nu (n=82) | 0.668 | 0.51 | 0.297 |  |  |  |  |  |  |  |  |  |  |  | |
| Jingpo (n=95) | 0 | 0.001 | 0.001 | 0 |  |  |  |  |  |  |  |  |  |  | |
| Bulang (n=109) | 0.04 | 0.508 | 0.793 | 0.19 | 0.001 |  |  |  |  |  |  |  |  |  | |
| Wa (n=109) | 0.824 | 0.359 | 0.179 | 0.84 | 0 | 0.1 |  |  |  |  |  |  |  |  | |
| Dai (n=121) | 0.002 | 0.118 | 0.218 | 0.032 | 0.009 | 0.268 | 0.01 |  |  |  |  |  |  |  | |
| Maonan (n=78) | 0.469 | 0.148 | 0.084 | 0.329 | 0 | 0.057 | 0.397 | 0.01 |  |  |  |  |  |  | |
| Zhuang (n=101) | 0.031 | 0.448 | 0.713 | 0.162 | 0.001 | 0.903 | 0.082 | 0.336 | 0.048 |  |  |  |  |  | |
| Tu (n=110) | 0 | 0 | 0 | 0 | 0.489 | 0 | 0 | 0.001 | 0 | 0 |  |  |  |  | |
| Yugur (n=93) | 0 | 0.002 | 0.004 | 0 | 0.652 | 0.003 | 0 | 0.035 | 0 | 0.005 | 0.251 |  |  |  | |
| Han-Yunnan (n=186) | 0.875 | 0.145 | 0.047 | 0.551 | 0 | 0.018 | 0.702 | 0 | 0.517 | 0.013 | 0 | 0 |  |  | |
| Japanese (n=100) | 0.882 | 0.772 | 0.472 | 0.682 | 0 | 0.303 | 0.513 | 0.044 | 0.21 | 0.258 | 0 | 0 | 0.248 |  | |
| Caucasians (n=174) | 0 | 0 | 0 | 0 | 0.002 | 0 | 0 | 0 | 0 | 0 | 0.012 | 0 | 0 | 0 | |
| DQA1 | | | | | | | | | | | | | | |  |
|  | Hani (n=149) | Jinuo (n=75) | Lisu (n=79) | Nu (n=82) | Jingpo (n=95) | Bulang (n=109) | Wa (n=109) | Dai (n=121) | Maonan (n=78) | Zhuang (n=101) | Tu (n=110) | Yugur (n=93) | Han-Yunnan (n=186) | Japanese (n=100) | |
| Jinuo (n=75) | 0 |  |  |  |  |  |  |  |  |  |  |  |  |  | |
| Lisu (n=79) | 0.086 | 0.002 |  |  |  |  |  |  |  |  |  |  |  |  | |
| Nu (n=82) | 0.041 | 0.001 | 0.791 |  |  |  |  |  |  |  |  |  |  |  | |
| Jingpo (n=95) | 0 | 0.129 | 0 | 0 |  |  |  |  |  |  |  |  |  |  | |
| Bulang (n=109) | 0 | 0.001 | 0 | 0 | 0.036 |  |  |  |  |  |  |  |  |  | |
| Wa (n=109) | 0.514 | 0.2 | 0.03 | 0.014 | 0.004 | 0 |  |  |  |  |  |  |  |  | |
| Dai (n=121) | 0.003 | 0.475 | 0 | 0 | 0.353 | 0.004 | 0.024 |  |  |  |  |  |  |  | |
| Maonan (n=78) | 0.001 | 0.086 | 0 | 0 | 0.743 | 0.08 | 0.003 | 0.235 |  |  |  |  |  |  | |
| Zhuang (n=101) | 0.755 | 0.043 | 0.195 | 0.112 | 0 | 0 | 0.373 | 0.002 | 0 |  |  |  |  |  | |
| Tu (n=110) | 0.003 | 0 | 0.353 | 0.512 | 0 | 0 | 0.001 | 0 | 0 | 0.017 |  |  |  |  | |
| Yugur (n=93) | 0.356 | 0.321 | 0.02 | 0.009 | 0.01 | 0 | 0.772 | 0.058 | 0.008 | 0.257 | 0.001 |  |  |  | |
| Han-Yunnan (n=186) | 0 | 0 | 0.002 | 0.005 | 0 | 0 | 0 | 0 | 0 | 0 | 0.019 | 0 |  |  | |
| Japanese (n=100) | 0 | 0 | 0 | 0 | 0 | 0 | 0 | 0 | 0 | 0 | 0 | 0 | 0.005 |  | |
| Caucasians (n=174) | 0 | 0 | 0 | 0 | 0 | 0 | 0 | 0 | 0 | 0 | 0 | 0 | 0 | 0.1 | |
| DRB1 | | | | | | | | | | | | | | |  |
|  | Hani (n=149) | Jinuo (n=75) | Lisu (n=79) | Nu (n=82) | Jingpo (n=95) | Bulang (n=109) | Wa (n=109) | Dai (n=121) | Maonan (n=78) | Zhuang (n=101) | Tu (n=110) | Yugur (n=93) | Han-Yunnan (n=186) | Japanese (n=100) | |
| Jinuo (n=75) | 0.834 |  |  |  |  |  |  |  |  |  |  |  |  |  | |
| Lisu (n=79) | 0.702 | 0.607 |  |  |  |  |  |  |  |  |  |  |  |  | |
| Nu (n=82) | 0.613 | 0.804 | 0.438 |  |  |  |  |  |  |  |  |  |  |  | |
| Jingpo (n=95) | 0.354 | 0.555 | 0.254 | 0.731 |  |  |  |  |  |  |  |  |  |  | |
| Bulang (n=109) | 0.184 | 0.363 | 0.14 | 0.505 | 0.744 |  |  |  |  |  |  |  |  |  | |
| Wa (n=109) | 0.048 | 0.042 | 0.009 | 0.068 | 0.122 | 0.205 |  |  |  |  |  |  |  |  | |
| Dai (n=121) | 0 | 0.002 | 0 | 0.004 | 0.007 | 0.014 | 0.248 |  |  |  |  |  |  |  | |
| Maonan (n=78) | 0.048 | 0.135 | 0.042 | 0.199 | 0.321 | 0.476 | 0.655 | 0.134 |  |  |  |  |  |  | |
| Zhuang (n=101) | 0 | 0 | 0 | 0 | 0 | 0 | 0.003 | 0.061 | 0.002 |  |  |  |  |  | |
| Tu (n=110) | 0.001 | 0.001 | 0.008 | 0 | 0 | 0 | 0 | 0 | 0 | 0 |  |  |  |  | |
| Yugur (n=93) | 0.068 | 0.075 | 0.208 | 0.038 | 0.013 | 0.004 | 0 | 0 | 0.001 | 0 | 0.164 |  |  |  | |
| Han-Yunnan (n=186) | 0.309 | 0.295 | 0.662 | 0.165 | 0.061 | 0.019 | 0 | 0 | 0.003 | 0 | 0.008 | 0.298 |  |  | |
| Japanese (n=100) | 0.718 | 0.911 | 0.508 | 0.879 | 0.603 | 0.386 | 0.036 | 0.001 | 0.134 | 0 | 0 | 0.044 | 0.197 |  | |
| Caucasians (n=174) | 0.185 | 0.395 | 0.144 | 0.557 | 0.831 | 0.88 | 0.117 | 0.004 | 0.359 | 0 | 0 | 0.003 | 0.014 | 0.42 | |
| ORF10 | | | | | | | | | | | | | | |  |
|  | Hani (n=149) | Jinuo (n=75) | Lisu (n=79) | Nu (n=82) | Jingpo (n=95) | Bulang (n=109) | Wa (n=109) | Dai (n=121) | Maonan (n=78) | Zhuang (n=101) | Tu (n=110) | Yugur (n=93) | Han-Yunnan (n=186) | Japanese (n=100) | |
| Jinuo (n=75) | 0.573 |  |  |  |  |  |  |  |  |  |  |  |  |  | |
| Lisu (n=79) | 0.146 | 0.463 |  |  |  |  |  |  |  |  |  |  |  |  | |
| Nu (n=82) | 0.479 | 0.915 | 0.519 |  |  |  |  |  |  |  |  |  |  |  | |
| Jingpo (n=95) | 0.003 | 0.053 | 0.223 | 0.059 |  |  |  |  |  |  |  |  |  |  | |
| Bulang (n=109) | 0.003 | 0.059 | 0.25 | 0.067 | 0.916 |  |  |  |  |  |  |  |  |  | |
| Wa (n=109) | 0.051 | 0.279 | 0.756 | 0.317 | 0.315 | 0.353 |  |  |  |  |  |  |  |  | |
| Dai (n=121) | 0 | 0 | 0 | 0 | 0 | 0 | 0 |  |  |  |  |  |  |  | |
| Maonan (n=78) | 0 | 0 | 0 | 0 | 0 | 0 | 0 | 0.859 |  |  |  |  |  |  | |
| Zhuang (n=101) | 0 | 0 | 0 | 0 | 0 | 0 | 0 | 0.975 | 0.886 |  |  |  |  |  | |
| Tu (n=110) | 0.039 | 0.242 | 0.688 | 0.275 | 0.362 | 0.405 | 0.921 | 0 | 0 | 0 |  |  |  |  | |
| Yugur (n=93) | 0.214 | 0.595 | 0.815 | 0.664 | 0.129 | 0.144 | 0.563 | 0 | 0 | 0 | 0.5 |  |  |  | |
| Han-Yunnan (n=186) | 0.001 | 0.032 | 0.167 | 0.035 | 0.975 | 0.877 | 0.236 | 0 | 0 | 0 | 0.281 | 0.083 |  |  | |
| Japanese (n=100) | 0.032 | 0.209 | 0.615 | 0.238 | 0.439 | 0.489 | 0.828 | 0 | 0 | 0 | 0.905 | 0.439 | 0.36 |  | |
| Caucasians (n=174) | 0 | 0 | 0.005 | 0 | 0.094 | 0.061 | 0.004 | 0.001 | 0.004 | 0.001 | 0.006 | 0.001 | 0.043 | 0.011 | |

**Supplementary Table 4. Hardy-Weinberg of the Five POALIN loci (P value) for 12 Chinese ethnic populations**

| Population | Alu No. | genotypes | | | Hardy-Weinberg |  | Population | Alu No. | genotypes | | | Hardy-Weinberg |
| --- | --- | --- | --- | --- | --- | --- | --- | --- | --- | --- | --- | --- |
|  |  |  | observed | expected | P value |  |  |  |  | observed | expected | P value |
| Hani | 1 | DPB2*1:DPB2*1 | 38 | 38.3 | 1 |  | Jinuo | 1 | DPB2*1:DPB2*1 | 29 | 28.8 | 1 |
|  |  | DPB2*2:DPB2*1 | 75 | 74.5 |  |  |  |  | DPB2*2:DPB2*1 | 35 | 35.3 |  |
|  |  | DPB2*2:DPB2*2 | 36 | 36.3 |  |  |  |  | DPB2*2:DPB2*2 | 11 | 10.8 |  |
|  | 2 | DQA2*1:DQA2*1 | 148 | 148 | 1 |  |  | 2 | DQA2*1:DQA2*1 | 74 | 73 | 0.0067 |
|  |  | DQA2*2:DQA2*1 | 1 | 1 |  |  |  |  | DQA2*2:DQA2*1 | 0 | 2 |  |
|  |  | DQA2*2:DQA2*2 | 0 | 0 |  |  |  |  | DQA2*2:DQA2*2 | 1 | 0 |  |
|  | 3 | DQA1*1:DQA1*1 | 14 | 2.3 | 0 |  |  | 3 | DQA1*1:DQA1*1 | 3 | 0.3 | 0.001 |
|  |  | DQA1*2:DQA1*1 | 9 | 32.4 |  |  |  |  | DQA1*2:DQA1*1 | 4 | 9.3 |  |
|  |  | DQA1*2:DQA1*2 | 126 | 114.3 |  |  |  |  | DQA1*2:DQA1*2 | 68 | 65.3 |  |
|  | 4 | DRB1*1:DRB1*1 | 95 | 92.7 | 0.2263 |  |  | 4 | DRB1*1:DRB1*1 | 46 | 45.6 | 0.7426 |
|  |  | DRB1*2:DRB1*1 | 45 | 49.7 |  |  |  |  | DRB1*2:DRB1*1 | 25 | 25.7 |  |
|  |  | DRB1*2:DRB1*2 | 9 | 6.7 |  |  |  |  | DRB1*2:DRB1*2 | 4 | 3.6 |  |
|  | 5 | ORF10*1:DRB10*1 | 128 | 125 | 0.0103 |  |  | 5 | ORF10*1:DRB10*1 | 61 | 60.8 | 0.5398 |
|  |  | ORF10*2:ORF10*1 | 17 | 22.9 |  |  |  |  | ORF10*2:ORF10*1 | 13 | 13.5 |  |
|  |  | ORF10*2:ORF10*2 | 4 | 1 |  |  |  |  | ORF10*2:ORF10*2 | 1 | 0.8 |  |
| Lisu | 1 | DPB2*1:DPB2*1 | 24 | 23.4 | 0.8221 |  | Nu | 1 | DPB2*1:DPB2*1 | 29 | 28.1 | 0.6554 |
|  |  | DPB2*2:DPB2*1 | 38 | 39.2 |  |  |  |  | DPB2*2:DPB2*1 | 38 | 39.8 |  |
|  |  | DPB2*2:DPB2*2 | 17 | 16.4 |  |  |  |  | DPB2*2:DPB2*2 | 15 | 14.1 |  |
|  | 2 | DQA2*1:DQA2*1 | 77 | 76 | 0.0192 |  |  | 2 | DQA2*1:DQA2*1 | 81 | 81 | 1 |
|  |  | DQA2*2:DQA2*1 | 1 | 2.9 |  |  |  |  | DQA2*2:DQA2*1 | 1 | 1 |  |
|  |  | DQA2*2:DQA2*2 | 1 | 0 |  |  |  |  | DQA2*2:DQA2*2 | 0 | 0 |  |
|  | 3 | DQA1*1:DQA1*1 | 13 | 2.7 | 0 |  |  | 3 | DQA1*1:DQA1*1 | 15 | 3.1 | 0 |
|  |  | DQA1*2:DQA1*1 | 3 | 23.7 |  |  |  |  | DQA1*2:DQA1*1 | 2 | 25.8 |  |
|  |  | DQA1*2:DQA1*2 | 63 | 52.7 |  |  |  |  | DQA1*2:DQA1*2 | 65 | 53.1 |  |
|  | 4 | DRB1*1:DRB1*1 | 51 | 51 | 1 |  |  | 4 | DRB1*1:DRB1*1 | 49 | 48.4 | 0.7557 |
|  |  | DRB1*2:DRB1*1 | 25 | 24.9 |  |  |  |  | DRB1*2:DRB1*1 | 28 | 29.2 |  |
|  |  | DRB1*2:DRB1*2 | 3 | 3 |  |  |  |  | DRB1*2:DRB1*2 | 5 | 4.4 |  |
|  | 5 | ORF10*1:DRB10*1 | 59 | 60.3 | 0.5992 |  |  | 5 | ORF10*1:DRB10*1 | 67 | 65.9 | 0.1928 |
|  |  | ORF10*2:ORF10*1 | 20 | 17.5 |  |  |  |  | ORF10*2:ORF10*1 | 13 | 15.2 |  |
|  |  | ORF10*2:ORF10*2 | 0 | 1.3 |  |  |  |  | ORF10*2:ORF10*2 | 2 | 0.9 |  |
| Jingpo | 1 | DPB2*1:DPB2*1 | 50 | 49.4 | 0.7987 |  | Bulang | 1 | DPB2*1:DPB2*1 | 44 | 37 | 0.006 |
|  |  | DPB2*2:DPB2*1 | 37 | 38.2 |  |  |  |  | DPB2*2:DPB2*1 | 39 | 53 |  |
|  |  | DPB2*2:DPB2*2 | 8 | 7.4 |  |  |  |  | DPB2*2:DPB2*2 | 26 | 19 |  |
|  | 2 | DQA2*1:DQA2*1 | 76 | 76.1 | 1 |  |  | 2 | DQA2*1:DQA2*1 | 104 | 104.1 | 1 |
|  |  | DQA2*2:DQA2*1 | 18 | 17.9 |  |  |  |  | DQA2*2:DQA2*1 | 5 | 4.9 |  |
|  |  | DQA2*2:DQA2*2 | 1 | 1.1 |  |  |  |  | DQA2*2:DQA2*2 | 0 | 0 |  |
|  | 3 | DQA1*1:DQA1*1 | 3 | 0.1 | 0 |  |  | 3 | DQA1*1:DQA1*1 | 0 | 0 | 1 |
|  |  | DQA1*2:DQA1*1 | 0 | 5.8 |  |  |  |  | DQA1*2:DQA1*1 | 1 | 1 |  |
|  |  | DQA1*2:DQA1*2 | 92 | 89.1 |  |  |  |  | DQA1*2:DQA1*2 | 108 | 108 |  |
|  | 4 | DRB1*1:DRB1*1 | 52 | 53.8 | 0.4169 |  |  | 4 | DRB1*1:DRB1*1 | 59 | 59.5 | 1 |
|  |  | DRB1*2:DRB1*1 | 39 | 35.4 |  |  |  |  | DRB1*2:DRB1*1 | 43 | 42.1 |  |
|  |  | DRB1*2:DRB1*2 | 4 | 5.8 |  |  |  |  | DRB1*2:DRB1*2 | 7 | 7.5 |  |
|  | 5 | ORF10*1:DRB10*1 | 65 | 64.9 | 1 |  |  | 5 | ORF10*1:DRB10*1 | 76 | 75.1 | 0.5057 |
|  |  | ORF10*2:ORF10*1 | 27 | 27.3 |  |  |  |  | ORF10*2:ORF10*1 | 29 | 30.7 |  |
|  |  | ORF10*2:ORF10*2 | 3 | 2.9 |  |  |  |  | ORF10*2:ORF10*2 | 4 | 3.1 |  |
| Wa | 1 | DPB2*1:DPB2*1 | 35 | 34.1 | 0.8462 |  | Dai | 1 | DPB2*1:DPB2*1 | 37 | 36 | 0.717 |
|  |  | DPB2*2:DPB2*1 | 52 | 53.7 |  |  |  |  | DPB2*2:DPB2*1 | 58 | 60 |  |
|  |  | DPB2*2:DPB2*2 | 22 | 21.1 |  |  |  |  | DPB2*2:DPB2*2 | 26 | 25 |  |
|  | 2 | DQA2*1:DQA2*1 | 108 | 108 | 1 |  |  | 2 | DQA2*1:DQA2*1 | 113 | 111.2 | 0.0103 |
|  |  | DQA2*2:DQA2*1 | 1 | 1 |  |  |  |  | DQA2*2:DQA2*1 | 6 | 9.6 |  |
|  |  | DQA2*2:DQA2*2 | 0 | 0 |  |  |  |  | DQA2*2:DQA2*2 | 2 | 0.2 |  |
|  | 3 | DQA1*1:DQA1*1 | 5 | 1.2 | 0.0018 |  |  | 3 | DQA1*1:DQA1*1 | 4 | 0.3 | 0 |
|  |  | DQA1*2:DQA1*1 | 13 | 20.6 |  |  |  |  | DQA1*2:DQA1*1 | 4 | 11.4 |  |
|  |  | DQA1*2:DQA1*2 | 91 | 87.2 |  |  |  |  | DQA1*2:DQA1*2 | 113 | 109.3 |  |
|  | 4 | DRB1*1:DRB1*1 | 50 | 50.9 | 0.8259 |  |  | 4 | DRB1*1:DRB1*1 | 45 | 48.4 | 0.243 |
|  |  | DRB1*2:DRB1*1 | 49 | 47.2 |  |  |  |  | DRB1*2:DRB1*1 | 63 | 56.3 |  |
|  |  | DRB1*2:DRB1*2 | 10 | 10.9 |  |  |  |  | DRB1*2:DRB1*2 | 13 | 16.4 |  |
|  | 5 | ORF10*1:DRB10*1 | 80 | 81.1 | 0.6873 |  |  | 5 | ORF10*1:DRB10*1 | 46 | 48.4 | 0.4352 |
|  |  | ORF10*2:ORF10*1 | 28 | 25.9 |  |  |  |  | ORF10*2:ORF10*1 | 61 | 56.3 |  |
|  |  | ORF10*2:ORF10*2 | 1 | 2.1 |  |  |  |  | ORF10*2:ORF10*2 | 14 | 16.4 |  |
| Maonan | 1 | DPB2*1:DPB2*1 | 34 | 32.1 | 0.3329 |  | Zhuang | 1 | DPB2*1:DPB2*1 | 30 | 33.3 | 0.2223 |
|  |  | DPB2*2:DPB2*1 | 32 | 35.9 |  |  |  |  | DPB2*2:DPB2*1 | 56 | 49.4 |  |
|  |  | DPB2*2:DPB2*2 | 12 | 10.1 |  |  |  |  | DPB2*2:DPB2*2 | 15 | 18.3 |  |
|  | 2 | DQA2*1:DQA2*1 | 78 | 0 | - |  |  | 2 | DQA2*1:DQA2*1 | 96 | 96.1 | 1 |
|  |  | DQA2*2:DQA2*1 | 0 | 0 |  |  |  |  | DQA2*2:DQA2*1 | 5 | 4.9 |  |
|  |  | DQA2*2:DQA2*2 | 0 | 0 |  |  |  |  | DQA2*2:DQA2*2 | 0 | 0.1 |  |
|  | 3 | DQA1*1:DQA1*1 | 1 | 0.1 | 0.0382 |  |  | 3 | DQA1*1:DQA1*1 | 13 | 1.8 | 0 |
|  |  | DQA1*2:DQA1*1 | 2 | 3.9 |  |  |  |  | DQA1*2:DQA1*1 | 1 | 23.4 |  |
|  |  | DQA1*2:DQA1*2 | 75 | 74.1 |  |  |  |  | DQA1*2:DQA1*2 | 87 | 75.8 |  |
|  | 4 | DRB1*1:DRB1*1 | 37 | 38.8 | 0.4228 |  |  | 4 | DRB1*1:DRB1*1 | 27 | 30 | 0.315 |
|  |  | DRB1*2:DRB1*1 | 36 | 32.4 |  |  |  |  | DRB1*2:DRB1*1 | 56 | 50.1 |  |
|  |  | DRB1*2:DRB1*2 | 5 | 6.8 |  |  |  |  | DRB1*2:DRB1*2 | 18 | 21 |  |
|  | 5 | ORF10*1:DRB10*1 | 34 | 32.1 | 0.3333 |  |  | 5 | ORF10*1:DRB10*1 | 39 | 40.6 | 0.6668 |
|  |  | ORF10*2:ORF10*1 | 32 | 35.9 |  |  |  |  | ORF10*2:ORF10*1 | 50 | 46.9 |  |
|  |  | ORF10*2:ORF10*2 | 12 | 10.1 |  |  |  |  | ORF10*2:ORF10*2 | 12 | 13.6 |  |
| Tu | 1 | DPB2*1:DPB2*1 | 28 | 26 | 0.4498 |  | Yugur | 1 | DPB2*1:DPB2*1 | 23 | 25.8 | 0.3015 |
|  |  | DPB2*2:DPB2*1 | 51 | 55 |  |  |  |  | DPB2*2:DPB2*1 | 52 | 46.4 |  |
|  |  | DPB2*2:DPB2*2 | 31 | 29 |  |  |  |  | DPB2*2:DPB2*2 | 18 | 20.8 |  |
|  | 2 | DQA2*1:DQA2*1 | 84 | 83.8 | 0.6826 |  |  | 2 | DQA2*1:DQA2*1 | 81 | 76.8 | 0.0001 |
|  |  | DQA2*2:DQA2*1 | 24 | 24.4 |  |  |  |  | DQA2*2:DQA2*1 | 7 | 15.4 |  |
|  |  | DQA2*2:DQA2*2 | 2 | 1.8 |  |  |  |  | DQA2*2:DQA2*2 | 5 | 0.8 |  |
|  | 3 | DQA1*1:DQA1*1 | 21 | 5.5 | 0 |  |  | 3 | DQA1*1:DQA1*1 | 5 | 0.9 | 0.0002 |
|  |  | DQA1*2:DQA1*1 | 7 | 38.1 |  |  |  |  | DQA1*2:DQA1*1 | 8 | 16.3 |  |
|  |  | DQA1*2:DQA1*2 | 82 | 66.5 |  |  |  |  | DQA1*2:DQA1*2 | 80 | 75.9 |  |
|  | 4 | DRB1*1:DRB1*1 | 89 | 89.1 | 1 |  |  | 4 | DRB1*1:DRB1*1 | 68 | 68 | 1 |
|  |  | DRB1*2:DRB1*1 | 20 | 19.8 |  |  |  |  | DRB1*2:DRB1*1 | 23 | 23.1 |  |
|  |  | DRB1*2:DRB1*2 | 1 | 1.1 |  |  |  |  | DRB1*2:DRB1*2 | 2 | 2 |  |
|  | 5 | ORF10*1:DRB10*1 | 82 | 81.2 | 0.4426 |  |  | 5 | ORF10*1:DRB10*1 | 75 | 72.3 | 0.0197 |
|  |  | ORF10*2:ORF10*1 | 25 | 26.6 |  |  |  |  | ORF10*2:ORF10*1 | 14 | 19.4 |  |
|  |  | ORF10*2:ORF10*2 | 3 | 2.2 |  |  |  |  | ORF10*2:ORF10*2 | 4 | 1.3 |  |

**Supplementary Table 5. The *P* values of 30 haplotypes at five loci in 15 populations**

| Haplotype No. | *P* Value (Bonferroni correction) | | | | | | | | | | | | | |
| --- | --- | --- | --- | --- | --- | --- | --- | --- | --- | --- | --- | --- | --- | --- |
| 1 | AluDPB2:AluDQA2:AluDQA1:AluDRB1:AluORF10 11111 | | | | | | | | | | | | | |
|  | Hani (n=149) | Jinuo (n=75) | Lisu (n=79) | Nu (n=82) | Jingpo (n=95) | Bulang (n=109) | Wa (n=109) | Dai (n=121) | Maonan (n=78) | Zhuang (n=101) | Tu (n=110) | Yugur (n=93) | Han-Yunnan (n=186) | Japanese (n=100) |
| Jinuo (n=75) | 0.134 |  |  |  |  |  |  |  |  |  |  |  |  |  |
| Lisu (n=79) | 0.018 | 0.489 |  |  |  |  |  |  |  |  |  |  |  |  |
| Nu (n=82) | 0.010 | 0.399 | 0.879 |  |  |  |  |  |  |  |  |  |  |  |
| Jingpo (n=95) | 0.260 | 0.025 | 0.004 | 0.003 |  |  |  |  |  |  |  |  |  |  |
| Bulang (n=109) | 0.202 | 0.015 | 0.002 | 0.001 | 0.922 |  |  |  |  |  |  |  |  |  |
| Wa (n=109) | 0.616 | 0.345 | 0.085 | 0.058 | 0.139 | 0.100 |  |  |  |  |  |  |  |  |
| Dai (n=121) | 0.384 | 0.031 | 0.004 | 0.002 | 0.709 | 0.625 | 0.199 |  |  |  |  |  |  |  |
| Maonan (n=78) | 0.358 | 0.049 | 0.011 | 0.007 | 0.889 | 0.812 | 0.210 | 0.835 |  |  |  |  |  |  |
| Zhuang (n=101) | 0.065 | 0.834 | 0.592 | 0.481 | 0.014 | 0.007 | 0.218 | 0.014 | 0.030 |  |  |  |  |  |
| Tu (n=110) | 0.001 | 0.145 | 0.442 | 0.537 | 0.000 | 0.000 | 0.009 | 0.000 | 0.002 | 0.163 |  |  |  |  |
| Yugur (n=93) | 0.157 | 0.895 | 0.384 | 0.300 | 0.031 | 0.018 | 0.396 | 0.037 | 0.059 | 0.716 | 0.092 |  |  |  |
| Han-Yunnan (n=186) | 0.000 | 0.021 | 0.094 | 0.124 | 0.000 | 0.000 | 0.000 | 0.000 | 0.000 | 0.016 | 0.314 | 0.008 |  |  |
| Japanese (n=100) | 0.000 | 0.000 | 0.001 | 0.001 | 0.000 | 0.000 | 0.000 | 0.000 | 0.000 | 0.000 | 0.002 | 0.000 | 0.013 |  |
| Caucasians (n=174) | 0.000 | 0.001 | 0.006 | 0.008 | 0.000 | 0.000 | 0.000 | 0.000 | 0.000 | 0.000 | 0.021 | 0.000 | 0.112 | 0.285 |
| 2 | AluDPB2:AluDQA2:AluDQA1:AluDRB1:AluORF10 11112 | | | | | | | | | | | | | |
|  | Hani (n=149) | Jinuo (n=75) | Lisu (n=79) | Nu (n=82) | Jingpo (n=95) | Bulang (n=109) | Wa (n=109) | Dai (n=121) | Maonan (n=78) | Zhuang (n=101) | Tu (n=110) | Yugur (n=93) | Han-Yunnan (n=186) | Japanese (n=100) |
| Jinuo (n=75) | - |  |  |  |  |  |  |  |  |  |  |  |  |  |
| Lisu (n=79) | 0.169 | 0.329 |  |  |  |  |  |  |  |  |  |  |  |  |
| Nu (n=82) | 0.056 | 0.175 | 0.584 |  |  |  |  |  |  |  |  |  |  |  |
| Jingpo (n=95) | 0.076 | 0.208 | 0.673 | 0.882 |  |  |  |  |  |  |  |  |  |  |
| Bulang (n=109) | - | - | 0.24 | 0.102 | 0.129 |  |  |  |  |  |  |  |  |  |
| Wa (n=109) | 0.098 | 0.239 | 0.76 | 0.774 | 0.89 | 0.156 |  |  |  |  |  |  |  |  |
| Dai (n=121) | 0.026 | 0.113 | 0.369 | 0.723 | 0.597 | 0.057 | 0.488 |  |  |  |  |  |  |  |
| Maonan (n=78) | - | - | 0.32 | 0.166 | 0.199 | - | 0.23 | 0.107 |  |  |  |  |  |  |
| Zhuang (n=101) | 0 | 0.004 | 0.012 | 0.03 | 0.015 | 0 | 0.007 | 0.028 | 0.003 |  |  |  |  |  |
| Tu (n=110) | 0.043 | 0.151 | 0.493 | 0.902 | 0.755 | 0.084 | 0.66 | 0.799 | 0.143 | 0.019 |  |  |  |  |
| Yugur (n=93) | 0.205 | 0.368 | 0.908 | 0.49 | 0.575 | 0.278 | 0.658 | 0.287 | 0.359 | 0.005 | 0.401 |  |  |  |
| Han-Yunnan (n=186) | 0.002 | 0.026 | 0.078 | 0.18 | 0.118 | 0.007 | 0.075 | 0.232 | 0.023 | 0.195 | 0.164 | 0.047 |  |  |
| Japanese (n=100) | - | - | 0.26 | 0.117 | 0.146 | - | 0.175 | 0.068 | - | 0.001 | 0.097 | 0.299 | 0.01 |  |
| Caucasians (n=174) | - | - | 0.137 | 0.039 | 0.055 | - | 0.073 | 0.016 | - | 0 | 0.029 | 0.171 | 0.001 | - |
| 3 | AluDPB2:AluDQA2:AluDQA1:AluDRB1:AluORF10 11121 | | | | | | | | | | | | | |
|  | Hani (n=149) | Jinuo (n=75) | Lisu (n=79) | Nu (n=82) | Jingpo (n=95) | Bulang (n=109) | Wa (n=109) | Dai (n=121) | Maonan (n=78) | Zhuang (n=101) | Tu (n=110) | Yugur (n=93) | Han-Yunnan (n=186) | Japanese (n=100) |
| Jinuo (n=75) | 0.315 |  |  |  |  |  |  |  |  |  |  |  |  |  |
| Lisu (n=79) | 0.962 | 0.329 |  |  |  |  |  |  |  |  |  |  |  |  |
| Nu (n=82) | 0.937 | 0.338 | 0.979 |  |  |  |  |  |  |  |  |  |  |  |
| Jingpo (n=95) | 0.258 | - | 0.272 | 0.281 |  |  |  |  |  |  |  |  |  |  |
| Bulang (n=109) | 0.226 | - | 0.24 | 0.248 | - |  |  |  |  |  |  |  |  |  |
| Wa (n=109) | 0.226 | - | 0.24 | 0.248 | - | - |  |  |  |  |  |  |  |  |
| Dai (n=121) | 0.202 | - | 0.215 | 0.224 | - | - | - |  |  |  |  |  |  |  |
| Maonan (n=78) | 0.305 | - | 0.32 | 0.329 | - | - | - | - |  |  |  |  |  |  |
| Zhuang (n=101) | 0.243 | - | 0.258 | 0.266 | - | - | - | - | - |  |  |  |  |  |
| Tu (n=110) | 0.228 | 0.097 | 0.32 | 0.301 | 0.062 | 0.045 | 0.045 | 0.035 | 0.09 | 0.054 |  |  |  |  |
| Yugur (n=93) | 0.856 | 0.368 | 0.908 | 0.929 | 0.312 | 0.278 | 0.278 | 0.253 | 0.359 | 0.297 | 0.244 |  |  |  |
| Han-Yunnan (n=186) | 0.395 | 0.154 | 0.479 | 0.456 | 0.108 | 0.086 | 0.086 | 0.07 | 0.146 | 0.098 | 0.649 | 0.384 |  |  |
| Japanese (n=100) | 0.183 | 0.082 | 0.274 | 0.257 | 0.05 | 0.036 | 0.036 | 0.027 | 0.076 | 0.043 | 0.892 | 0.204 | 0.548 |  |
| Caucasians (n=174) | 0.006 | 0.013 | 0.037 | 0.033 | 0.005 | 0.003 | 0.003 | 0.002 | 0.011 | 0.004 | 0.144 | 0.02 | 0.025 | 0.201 |
| 4 | AluDPB2:AluDQA2:AluDQA1:AluDRB1:AluORF10 11122 | | | | | | | | | | | | | |
|  | Hani (n=149) | Jinuo (n=75) | Lisu (n=79) | Nu (n=82) | Jingpo (n=95) | Bulang (n=109) | Wa (n=109) | Dai (n=121) | Maonan (n=78) | Zhuang (n=101) | Tu (n=110) | Yugur (n=93) | Han-Yunnan (n=186) | Caucasians (n=174) |
| Japanese (n=100) | 0.222 | 0.386 | 0.373 | 0.365 | 0.329 | 0.296 | 0.296 | 0.271 | 0.376 | 0.314 | 0.294 | 0.334 | 0.172 | 0.187 |
| 5 | AluDPB2:AluDQA2:AluDQA1:AluDRB1:AluORF10 11211 | | | | | | | | | | | | | |
|  | Hani (n=149) | Jinuo (n=75) | Lisu (n=79) | Nu (n=82) | Jingpo (n=95) | Bulang (n=109) | Wa (n=109) | Dai (n=121) | Maonan (n=78) | Zhuang (n=101) | Tu (n=110) | Yugur (n=93) | Han-Yunnan (n=186) | Japanese (n=100) |
| Jinuo (n=75) | 0.254 |  |  |  |  |  |  |  |  |  |  |  |  |  |
| Lisu (n=79) | 0.945 | 0.349 |  |  |  |  |  |  |  |  |  |  |  |  |
| Nu (n=82) | 0.252 | 0.046 | 0.285 |  |  |  |  |  |  |  |  |  |  |  |
| Jingpo (n=95) | 0.027 | 0.403 | 0.067 | 0.003 |  |  |  |  |  |  |  |  |  |  |
| Bulang (n=109) | 0.627 | 0.183 | 0.63 | 0.506 | 0.012 |  |  |  |  |  |  |  |  |  |
| Wa (n=109) | 0.779 | 0.403 | 0.862 | 0.186 | 0.07 | 0.475 |  |  |  |  |  |  |  |  |
| Dai (n=121) | 0.007 | 0.001 | 0.017 | 0.224 | 0 | 0.041 | 0.005 |  |  |  |  |  |  |  |
| Maonan (n=78) | 0.418 | 0.09 | 0.439 | 0.776 | 0.009 | 0.724 | 0.316 | 0.13 |  |  |  |  |  |  |
| Zhuang (n=101) | 0 | 0 | 0 | 0.001 | 0 | 0 | 0 | 0.025 | 0 |  |  |  |  |  |
| Tu (n=110) | 0.006 | 0.001 | 0.015 | 0.194 | 0 | 0.035 | 0.005 | 0.907 | 0.113 | 0.036 |  |  |  |  |
| Yugur (n=93) | 0.555 | 0.123 | 0.566 | 0.595 | 0.012 | 0.906 | 0.422 | 0.065 | 0.816 | 0 | 0.056 |  |  |  |
| Han-Yunnan (n=186) | 0.052 | 0.005 | 0.093 | 0.682 | 0 | 0.206 | 0.038 | 0.311 | 0.459 | 0.001 | 0.268 | 0.285 |  |  |
| Japanese (n=100) | 0 | 0 | 0 | 0 | 0 | 0 | 0 | 0.005 | 0 | 0.585 | 0.009 | 0 | 0 |  |
| Caucasians (n=174) | 0 | 0 | 0 | 0.001 | 0 | 0 | 0 | 0.021 | 0 | 0.782 | 0.033 | 0 | 0 | 0.379 |
| 6 | AluDPB2:AluDQA2:AluDQA1:AluDRB1:AluORF10 11212 | | | | | | | | | | | | | |
|  | Hani (n=149) | Jinuo (n=75) | Lisu (n=79) | Nu (n=82) | Jingpo (n=95) | Bulang (n=109) | Wa (n=109) | Dai (n=121) | Maonan (n=78) | Zhuang (n=101) | Tu (n=110) | Yugur (n=93) | Han-Yunnan (n=186) | Japanese (n=100) |
| Jinuo (n=75) | - |  |  |  |  |  |  |  |  |  |  |  |  |  |
| Lisu (n=79) | 0.052 | 0.167 |  |  |  |  |  |  |  |  |  |  |  |  |
| Nu (n=82) | 0 | 0.006 | 0.062 |  |  |  |  |  |  |  |  |  |  |  |
| Jingpo (n=95) | 0.001 | 0.018 | 0.157 | 0.578 |  |  |  |  |  |  |  |  |  |  |
| Bulang (n=109) | 0 | 0.002 | 0.014 | 0.521 | 0.212 |  |  |  |  |  |  |  |  |  |
| Wa (n=109) | 0.019 | 0.095 | 0.664 | 0.091 | 0.25 | 0.016 |  |  |  |  |  |  |  |  |
| Dai (n=121) | 0 | 0.002 | 0.017 | 0.572 | 0.238 | 0.921 | 0.019 |  |  |  |  |  |  |  |
| Maonan (n=78) | 0 | 0 | 0 | 0.002 | 0 | 0.005 | 0 | 0.003 |  |  |  |  |  |  |
| Zhuang (n=101) | 0.003 | 0.033 | 0.276 | 0.344 | 0.693 | 0.097 | 0.446 | 0.111 | 0 |  |  |  |  |  |
| Tu (n=110) | 0 | 0.012 | 0.107 | 0.711 | 0.832 | 0.274 | 0.164 | 0.308 | 0 | 0.535 |  |  |  |  |
| Yugur (n=93) | 0 | 0.004 | 0.038 | 0.833 | 0.43 | 0.658 | 0.052 | 0.719 | 0.002 | 0.234 | 0.541 |  |  |  |
| Han-Yunnan (n=186) | 0 | 0.013 | 0.098 | 0.656 | 0.84 | 0.195 | 0.144 | 0.224 | 0 | 0.419 | 0.972 | 0.469 |  |  |
| Japanese (n=100) | 0 | 0.005 | 0.051 | 0.957 | 0.525 | 0.532 | 0.072 | 0.587 | 0.001 | 0.298 | 0.654 | 0.868 | 0.589 |  |
| Caucasians (n=174) | 0.008 | 0.061 | 0.439 | 0.118 | 0.351 | 0.014 | 0.709 | 0.016 | 0 | 0.63 | 0.222 | 0.06 | 0.186 | 0.088 |
| 7 | AluDPB2:AluDQA2:AluDQA1:AluDRB1:AluORF10 11221 | | | | | | | | | | | | | |
|  | Hani (n=149) | Jinuo (n=75) | Lisu (n=79) | Nu (n=82) | Jingpo (n=95) | Bulang (n=109) | Wa (n=109) | Dai (n=121) | Maonan (n=78) | Zhuang (n=101) | Tu (n=110) | Yugur (n=93) | Han-Yunnan (n=186) | Japanese (n=100) |
| Jinuo (n=75) | 0.239 |  |  |  |  |  |  |  |  |  |  |  |  |  |
| Lisu (n=79) | 0.869 | 0.251 |  |  |  |  |  |  |  |  |  |  |  |  |
| Nu (n=82) | 0.001 | 0.102 | 0.005 |  |  |  |  |  |  |  |  |  |  |  |
| Jingpo (n=95) | 0.676 | 0.481 | 0.612 | 0.014 |  |  |  |  |  |  |  |  |  |  |
| Bulang (n=109) | 0.003 | 0.175 | 0.011 | 0.698 | 0.027 |  |  |  |  |  |  |  |  |  |
| Wa (n=109) | 0.079 | 0.709 | 0.111 | 0.153 | 0.245 | 0.266 |  |  |  |  |  |  |  |  |
| Dai (n=121) | 0.704 | 0.423 | 0.634 | 0.007 | 0.935 | 0.015 | 0.191 |  |  |  |  |  |  |  |
| Maonan (n=78) | 0.551 | 0.619 | 0.51 | 0.032 | 0.856 | 0.059 | 0.362 | 0.805 |  |  |  |  |  |  |
| Zhuang (n=101) | 0.018 | 0.37 | 0.037 | 0.396 | 0.088 | 0.617 | 0.554 | 0.059 | 0.154 |  |  |  |  |  |
| Tu (n=110) | 0.118 | 0.013 | 0.219 | 0 | 0.068 | 0 | 0.002 | 0.065 | 0.052 | 0 |  |  |  |  |
| Yugur (n=93) | 0.871 | 0.233 | 0.992 | 0.003 | 0.603 | 0.007 | 0.094 | 0.624 | 0.498 | 0.028 | 0.199 |  |  |  |
| Han-Yunnan (n=186) | 0.167 | 0.015 | 0.326 | 0 | 0.092 | 0 | 0.002 | 0.085 | 0.069 | 0 | 0.673 | 0.296 |  |  |
| Japanese (n=100) | 0.225 | 0.96 | 0.244 | 0.068 | 0.484 | 0.124 | 0.645 | 0.42 | 0.631 | 0.303 | 0.011 | 0.224 | 0.01 |  |
| Caucasians (n=174) | 0.001 | 0 | 0.005 | 0 | 0 | 0 | 0 | 0 | 0 | 0 | 0.145 | 0.004 | 0.038 | 0 |
| 8 | AluDPB2:AluDQA2:AluDQA1:AluDRB1:AluORF10 11222 | | | | | | | | | | | | | |
|  | Hani (n=149) | Jinuo (n=75) | Lisu (n=79) | Nu (n=82) | Jingpo (n=95) | Bulang (n=109) | Wa (n=109) | Dai (n=121) | Maonan (n=78) | Zhuang (n=101) | Tu (n=110) | Yugur (n=93) | Han-Yunnan (n=186) | Japanese (n=100) |
| Jinuo (n=75) | 0.985 |  |  |  |  |  |  |  |  |  |  |  |  |  |
| Lisu (n=79) | 0.219 | 0.278 |  |  |  |  |  |  |  |  |  |  |  |  |
| Nu (n=82) | 0 | 0 | 0.006 |  |  |  |  |  |  |  |  |  |  |  |
| Jingpo (n=95) | 0.534 | 0.591 | 0.098 | 0 |  |  |  |  |  |  |  |  |  |  |
| Bulang (n=109) | 0.01 | 0.02 | 0.245 | 0.051 | 0.003 |  |  |  |  |  |  |  |  |  |
| Wa (n=109) | 0.284 | 0.363 | 0.783 | 0.004 | 0.12 | 0.126 |  |  |  |  |  |  |  |  |
| Dai (n=121) | 0.05 | 0.111 | 0.007 | 0 | 0.253 | 0 | 0.006 |  |  |  |  |  |  |  |
| Maonan (n=78) | 0.409 | 0.474 | 0.072 | 0 | 0.831 | 0.002 | 0.087 | 0.392 |  |  |  |  |  |  |
| Zhuang (n=101) | 0.018 | 0.49 | 0.003 | 0 | 0.127 | 0 | 0.002 | 0.648 | 0.22 |  |  |  |  |  |
| Tu (n=110) | 0.001 | 0.001 | 0.027 | 0.221 | 0 | 0.248 | 0.011 | 0 | 0 | 0 |  |  |  |  |
| Yugur (n=93) | 0.001 | 0.001 | 0.017 | 0.347 | 0 | 0.146 | 0.008 | 0 | 0 | 0 | 0.663 |  |  |  |
| Han-Yunnan (n=186) | 0.059 | 0.117 | 0.834 | 0.009 | 0.017 | 0.26 | 0.563 | 0 | 0.011 | 0 | 0.028 | 0.02 |  |  |
| Japanese (n=100) | 0 | 0 | 0.003 | - | 0 | 0.031 | 0.001 | 0 | 0 | 0 | 0.176 | 0.299 | 0.004 |  |
| Caucasians (n=174) | 0.026 | 0.058 | 0.59 | 0.016 | 0.007 | 0.433 | 0.349 | 0 | 0.004 | 0 | 0.057 | 0.038 | 0.68 | 0.008 |
| 9 | AluDPB2:AluDQA2:AluDQA1:AluDRB1:AluORF10 12111 | | | | | | | | | | | | | |
|  | Hani (n=149) | Jinuo (n=75) | Lisu (n=79) | Nu (n=82) | Jingpo (n=95) | Bulang (n=109) | Wa (n=109) | Dai (n=121) | Maonan (n=78) | Zhuang (n=101) | Tu (n=110) | Yugur (n=93) | Han-Yunnan (n=186) | Japanese (n=100) |
| Jinuo (n=75) | - |  |  |  |  |  |  |  |  |  |  |  |  |  |
| Lisu (n=79) | 0.169 | 0.329 |  |  |  |  |  |  |  |  |  |  |  |  |
| Nu (n=82) | - | - | 0.308 |  |  |  |  |  |  |  |  |  |  |  |
| Jingpo (n=95) | - | - | 0.272 | - |  |  |  |  |  |  |  |  |  |  |
| Bulang (n=109) | - | - | 0.24 | - | - |  |  |  |  |  |  |  |  |  |
| Wa (n=109) | - | - | 0.24 | - | - | - |  |  |  |  |  |  |  |  |
| Dai (n=121) | 0.054 | 0.171 | 0.551 | 0.152 | 0.124 | 0.099 | 0.099 |  |  |  |  |  |  |  |
| Maonan (n=78) | - | - | 0.32 | - | - | - | - | 0.163 |  |  |  |  |  |  |
| Zhuang (n=101) | - | - | 0.258 | - | - | - | - | 0.112 | - |  |  |  |  |  |
| Tu (n=110) | 0.019 | 0.097 | 0.032 | 0.083 | 0.062 | 0.045 | 0.045 | 0.611 | 0.09 | 0.054 |  |  |  |  |
| Yugur (n=93) | 0.028 | 0.118 | 0.398 | 0.102 | 0.079 | 0.06 | 0.06 | 0.745 | 0.111 | 0.07 | 0.874 |  |  |  |
| Han-Yunnan (n=186) | - | - | 0.125 | - | - | - | - | 0.031 | - | - | 0.009 | 0.014 |  |  |
| Japanese (n=100) | 0.084 | 0.219 | 0.705 | 0.199 | 0.167 | 0.139 | 0.139 | 0.813 | 0.21 | 0.154 | 0.055 | 0.595 | 0.053 |  |
| Caucasians (n=174) | 0 | 0 | 0.001 | 0 | 0 | 0 | 0 | 0 | 0 | 0 | 0.012 | 0.002 | 0 | 0 |
| 10 | AluDPB2:AluDQA2:AluDQA1:AluDRB1:AluORF10 12112 | | | | | | | | | | | | | |
|  | Hani (n=149) | Jinuo (n=75) | Lisu (n=79) | Nu (n=82) | Jingpo (n=95) | Bulang (n=109) | Wa (n=109) | Dai (n=121) | Maonan (n=78) | Zhuang (n=101) | Tu (n=110) | Yugur (n=93) | Han-Yunnan (n=186) | Japanese (n=100) |
| Caucasians (n=174) | 0.354 | 0.511 | 0.5 | 0.492 | 0.46 | 0.428 | 0.428 | 0.404 | 0.503 | 0.446 | 0.426 | 0.464 | 0.301 | 0.448 |
| 11 | AluDPB2:AluDQA2:AluDQA1:AluDRB1:AluORF10 12121 | | | | | | | | | | | | | |
|  | Jinuo (n=75) | Lisu (n=79) | Nu (n=82) | Jingpo (n=95) | Bulang (n=109) | Wa (n=109) | Dai (n=121) | Maonan (n=78) | Zhuang (n=101) | Tu (n=110) | Yugur (n=93) | Han-Yunnan (n=186) | Japanese (n=100) | Caucasians (n=174) |
| Hani (n=149) | 0.478 | 0.466 | 0.458 | 0.424 | 0.392 | 0.392 | 0.367 | 0.469 | 0.41 | 0.39 | 0.429 | 0.264 | 0.412 | 0.279 |
| 12 | AluDPB2:AluDQA2:AluDQA1:AluDRB1:AluORF10 12122 | | | | | | | | | | | | | |
|  | Hani (n=149) | Jinuo (n=75) | Lisu (n=79) | Nu (n=82) | Jingpo (n=95) | Bulang (n=109) | Wa (n=109) | Dai (n=121) | Maonan (n=78) | Zhuang (n=101) | Tu (n=110) | Yugur (n=93) | Han-Yunnan (n=186) | Japanese (n=100) |
| Caucasians (n=174) | 0.19 | 0.352 | 0.34 | 0.331 | 0.295 | 0.262 | 0.262 | 0.237 | 0.343 | 0.28 | 0.26 | 0.3 | 0.143 | 0.283 |
| 13 | AluDPB2:AluDQA2:AluDQA1:AluDRB1:AluORF10 12211 | | | | | | | | | | | | | |
|  | Hani (n=149) | Jinuo (n=75) | Lisu (n=79) | Nu (n=82) | Jingpo (n=95) | Bulang (n=109) | Wa (n=109) | Dai (n=121) | Maonan (n=78) | Zhuang (n=101) | Tu (n=110) | Yugur (n=93) | Han-Yunnan (n=186) | Japanese (n=100) |
| Jinuo (n=75) | - |  |  |  |  |  |  |  |  |  |  |  |  |  |
| Lisu (n=79) | 0.169 | 0.329 |  |  |  |  |  |  |  |  |  |  |  |  |
| Nu (n=82) | 0.177 | 0.338 | 0.979 |  |  |  |  |  |  |  |  |  |  |  |
| Jingpo (n=95) | 0 | 0.004 | 0.014 | 0.012 |  |  |  |  |  |  |  |  |  |  |
| Bulang (n=109) | - | - | 0.24 | 0.248 | 0.001 |  |  |  |  |  |  |  |  |  |
| Wa (n=109) | - | - | 0.24 | 0.248 | 0.001 | - |  |  |  |  |  |  |  |  |
| Dai (n=121) | 0.116 | 0.264 | 0.826 | 0.802 | 0.005 | 0.179 | 0.179 |  |  |  |  |  |  |  |
| Maonan (n=78) | - | - | 0.32 | 0.329 | 0.004 | - | - | 0.255 |  |  |  |  |  |  |
| Zhuang (n=101) | 0.085 | 0.222 | 0.711 | 0.688 | 0.014 | 0.141 | 0.141 | 0.856 | 0.213 |  |  |  |  |  |
| Tu (n=110) | 0 | 0.005 | 0.017 | 0.014 | 0.904 | 0.001 | 0.001 | 0.007 | 0.005 | 0.017 |  |  |  |  |
| Yugur (n=93) | 0.011 | 0.071 | 0.241 | 0.225 | 0.111 | 0.03 | 0.03 | 0.248 | 0.65 | 0.355 | 0.129 |  |  |  |
| Han-Yunnan (n=186) | 0.37 | 0.525 | 0.532 | 0.551 | 0 | 0.444 | 0.444 | 0.333 | 0.517 | 0.252 | 0 | 0.026 |  |  |
| Japanese (n=100) | - | - | 0.26 | 0.269 | 0.001 | - | - | 0.198 | - | 0.158 | 0.001 | 0.037 | 0.463 |  |
| Caucasians (n=174) | 0.038 | 0.14 | 0.439 | 0.417 | 0.01 | 0.075 | 0.075 | 0.501 | 0.132 | 0.652 | 0.012 | 0.542 | 0.085 | 0.089 |
| 14 | AluDPB2:AluDQA2:AluDQA1:AluDRB1:AluORF10 12212 | | | | | | | | | | | | | |
|  | Hani (n=149) | Jinuo (n=75) | Lisu (n=79) | Nu (n=82) | Jingpo (n=95) | Bulang (n=109) | Wa (n=109) | Dai (n=121) | Maonan (n=78) | Yugur (n=93) | Han-Yunnan (n=186) | Japanese (n=100) | Zhuang (n=101) | Tu (n=110) |
| Zhuang (n=101) | 0.224 | 0.388 | 0.376 | 0.367 | 0.332 | 0.298 | 0.298 | 0.273 | 0.379 | 0.337 | 0.174 | 0.319 |  |  |
| Tu (n=110) | 0.244 | 0.408 | 0.396 | 0.387 | 0.352 | 0.319 | 0.319 | 0.294 | 0.399 | 0.357 | 0.193 | 0.34 | 0.952 |  |
| Caucasians (n=174) | 0.001 | 0.021 | 0.018 | 0.016 | 0.01 | 0.006 | 0.006 | 0.004 | 0.019 | 0.01 | 0 | 0.008 | 0.028 | 0.02 |
| 15 | AluDPB2:AluDQA2:AluDQA1:AluDRB1:AluORF10 12221 | | | | | | | | | | | | | |
|  | Hani (n=149) | Lisu (n=79) | Nu (n=82) | Jingpo (n=95) | Bulang (n=109) | Wa (n=109) | Maonan (n=78) | Zhuang (n=101) | Tu (n=110) | Yugur (n=93) | Han-Yunnan (n=186) | Japanese (n=100) | Caucasians (n=174) | Jinuo (n=75) |
| Jinuo (n=75) | 0.046 | 0.145 | 0.138 | 0.11 | 0.087 | 0.087 | 0.148 | 0.1 | 0.086 | 0.114 | 0.026 | 0.101 | 0.031 |  |
| Dai (n=121) | 0.267 | 0.418 | 0.41 | 0.375 | 0.342 | 0.342 | 0.421 | 0.36 | 0.34 | 0.38 | 0.251 | 0.363 | 0.23 | 0.31 |
| 16 | AluDPB2:AluDQA2:AluDQA1:AluDRB1:AluORF10 12222 | | | | | | | | | | | | | |
|  | Hani (n=149) | Jinuo (n=75) | Lisu (n=79) | Nu (n=82) | Wa (n=109) | Dai (n=121) | Maonan (n=78) | Tu (n=110) | Han-Yunnan (n=186) | Japanese (n=100) | Caucasians (n=174) | Jingpo (n=95) | Bulang (n=109) | Zhuang (n=101) |
| Jingpo (n=95) | 0.076 | 0.208 | 0.196 | 0.188 | 0.129 | 0.11 | 0.199 | 0.127 | 0.047 | 0.146 | 0.055 |  |  |  |
| Bulang (n=109) | 0.009 | 0.062 | 0.055 | 0.051 | 0.025 | 0.018 | 0.057 | 0.024 | 0.003 | 0.031 | 0.005 | 0.336 |  |  |
| Zhuang (n=101) | 0.224 | 0.388 | 0.376 | 0.367 | 0.298 | 0.273 | 0.379 | 0.296 | 0.174 | 0.319 | 0.189 | 0.527 | 0.121 |  |
| Yugur (n=93) | 0.205 | 0.368 | 0.356 | 0.347 | 0.278 | 0.253 | 0.359 | 0.276 | 0.157 | 0.299 | 0.171 | 0.575 | 0.146 | 0.953 |
| 17 | AluDPB2:AluDQA2:AluDQA1:AluDRB1:AluORF10 21111 | | | | | | | | | | | | | |
|  | Hani (n=149) | Jinuo (n=75) | Lisu (n=79) | Nu (n=82) | Jingpo (n=95) | Bulang (n=109) | Wa (n=109) | Dai (n=121) | Maonan (n=78) | Zhuang (n=101) | Tu (n=110) | Yugur (n=93) | Han-Yunnan (n=186) | Japanese (n=100) |
| Jinuo (n=75) | 0.004 |  |  |  |  |  |  |  |  |  |  |  |  |  |
| Lisu (n=79) | 0.793 | 0.003 |  |  |  |  |  |  |  |  |  |  |  |  |
| Nu (n=82) | 0.579 | 0.001 | 0.804 |  |  |  |  |  |  |  |  |  |  |  |
| Jingpo (n=95) | 0 | 0.471 | 0 | 0 |  |  |  |  |  |  |  |  |  |  |
| Bulang (n=109) | 0 | 0.036 | 0 | 0 | 0.129 |  |  |  |  |  |  |  |  |  |
| Wa (n=109) | 0.313 | 0.033 | 0.259 | 0.159 | 0.003 | 0 |  |  |  |  |  |  |  |  |
| Dai (n=121) | 0 | 0.027 | 0 | 0 | 0.11 | - | 0 |  |  |  |  |  |  |  |
| Maonan (n=78) | 0.001 | 0.62 | 0.001 | 0 | 0.843 | 0.094 | 0.01 | 0.077 |  |  |  |  |  |  |
| Zhuang (n=101) | 0.001 | 0.99 | 0.001 | 0 | 0.455 | 0.037 | 0.016 | 0.028 | 0.61 |  |  |  |  |  |
| Tu (n=110) | 0.775 | 0.008 | 0.622 | 0.442 | 0.001 | 0 | 0.492 | 0 | 0.002 | 0.003 |  |  |  |  |
| Yugur (n=93) | 0.005 | 0.681 | 0.004 | 0.002 | 0.241 | 0.015 | 0.053 | 0.01 | 0.36 | 0.643 | 0.011 |  |  |  |
| Han-Yunnan (n=186) | 0.013 | 0 | 0.083 | 0.137 | 0 | 0 | 0.001 | 0 | 0 | 0 | 0.012 | 0 |  |  |
| Japanese (n=100) | 0.89 | 0.006 | 0.718 | 0.53 | 0 | 0 | 0.422 | 0 | 0.002 | 0.002 | 0.896 | 0.009 | 0.022 |  |
| Caucasians (n=174) | 0.941 | 0.004 | 0.74 | 0.527 | 0 | 0 | 0.331 | 0 | 0.001 | 0.001 | 0.82 | 0.005 | 0.007 | 0.939 |
| 18 | AluDPB2:AluDQA2:AluDQA1:AluDRB1:AluORF10 21112 | | | | | | | | | | | | | |
|  | Hani (n=149) | Jinuo (n=75) | Lisu (n=79) | Nu (n=82) | Jingpo (n=95) | Bulang (n=109) | Wa (n=109) | Dai (n=121) | Maonan (n=78) | Zhuang (n=101) | Tu (n=110) | Yugur (n=93) | Han-Yunnan (n=186) | Japanese (n=100) |
| Jinuo (n=75) | 0.158 |  |  |  |  |  |  |  |  |  |  |  |  |  |
| Lisu (n=79) | - | 0.304 |  |  |  |  |  |  |  |  |  |  |  |  |
| Nu (n=82) | 0.177 | 0.95 | 0.326 |  |  |  |  |  |  |  |  |  |  |  |
| Jingpo (n=95) | 0.21 | 0.867 | 0.361 | 0.917 |  |  |  |  |  |  |  |  |  |  |
| Bulang (n=109) | - | 0.227 | - | 0.248 | 0.284 |  |  |  |  |  |  |  |  |  |
| Wa (n=109) | - | 0.227 | - | 0.248 | 0.284 | - |  |  |  |  |  |  |  |  |
| Dai (n=121) | 0.026 | 0.398 | 0.104 | 0.35 | 0.277 | 0.057 | 0.057 |  |  |  |  |  |  |  |
| Maonan (n=78) | 0.05 | 0.585 | 0.153 | 0.533 | 0.451 | 0.094 | 0.094 | 0.767 |  |  |  |  |  |  |
| Zhuang (n=101) | 0.085 | 0.744 | 0.21 | 0.688 | 0.598 | 0.141 | 0.141 | 0.547 | 0.794 |  |  |  |  |  |
| Tu (n=110) | 0.099 | 0.798 | 0.229 | 0.742 | 0.65 | 0.158 | 0.158 | 0.481 | 0.728 | 0.932 |  |  |  |  |
| Yugur (n=93) | - | 0.265 | - | 0.286 | 0.322 | - | - | 0.078 | 0.121 | 0.174 | 0.192 |  |  |  |
| Han-Yunnan (n=186) | - | 0.115 | - | 0.132 | 0.161 | - | - | 0.013 | 0.029 | 0.055 | 0.065 | - |  |  |
| Japanese (n=100) | 0 | 0.009 | 0.002 | 0.006 | 0.003 | 0 | 0 | 0.015 | 0.023 | 0.006 | 0.004 | 0.001 | 0 |  |
| Caucasians (n=174) | 0.038 | 0.47 | 0.13 | 0.417 | 0.336 | 0.075 | 0.075 | 0.833 | 0.891 | 0.652 | 0.579 | 0.1 | 0.02 | 0.003 |
| 19 | AluDPB2:AluDQA2:AluDQA1:AluDRB1:AluORF10 21121 | | | | | | | | | | | | | |
|  | Jinuo (n=75) | Lisu (n=79) | Nu (n=82) | Jingpo (n=95) | Bulang (n=109) | Wa (n=109) | Dai (n=121) | Maonan (n=78) | Zhuang (n=101) | Tu (n=110) | Yugur (n=93) | Hani (n=149) | Han-Yunnan (n=186) | Japanese (n=100) |
| Hani (n=149) | 0.478 | 0.466 | 0.458 | 0.424 | 0.392 | 0.392 | 0.367 | 0.469 | 0.41 | 0.39 | 0.429 |  |  |  |
| Han-Yunnan (n=186) | 0.37 | 0.35 | 0.348 | 0.313 | 0.279 | 0.279 | 0.255 | 0.36 | 0.298 | 0.277 | 0.318 | 0.7 |  |  |
| Japanese (n=100) | 0.005 | 0.004 | 0.004 | 0.002 | 0.001 | 0.001 | 0 | 0.005 | 0.001 | 0.001 | 0.002 | 0.001 | 0 |  |
| Caucasians (n=174) | 0.005 | 0.004 | 0.003 | 0.001 | 0.001 | 0.001 | 0 | 0.004 | 0.001 | 0.001 | 0.002 | 0 | 0 | 0.93 |
| 20 | AluDPB2:AluDQA2:AluDQA1:AluDRB1:AluORF10 21122 | | | | | | | | | | | | | |
|  | Hani (n=149) | Jinuo (n=75) | Lisu (n=79) | Nu (n=82) | Jingpo (n=95) | Bulang (n=109) | Wa (n=109) | Dai (n=121) | Maonan (n=78) | Zhuang (n=101) | Tu (n=110) | Yugur (n=93) | Han-Yunnan (n=186) | Japanese (n=100) |
| Caucasians (n=174) | 0.354 | 0.511 | 0.5 | 0.492 | 0.46 | 0.428 | 0.428 | 0.404 | 0.503 | 0.446 | 0.426 | 0.464 | 0.301 | 0.448 |
| 21 | AluDPB2:AluDQA2:AluDQA1:AluDRB1:AluORF10 21211 | | | | | | | | | | | | | |
|  | Hani (n=149) | Jinuo (n=75) | Lisu (n=79) | Nu (n=82) | Jingpo (n=95) | Bulang (n=109) | Wa (n=109) | Dai (n=121) | Maonan (n=78) | Zhuang (n=101) | Tu (n=110) | Yugur (n=93) | Han-Yunnan (n=186) | Japanese (n=100) |
| Jinuo (n=75) | 0.588 |  |  |  |  |  |  |  |  |  |  |  |  |  |
| Lisu (n=79) | 0.021 | 0.12 |  |  |  |  |  |  |  |  |  |  |  |  |
| Nu (n=82) | 0.002 | 0.023 | 0.475 |  |  |  |  |  |  |  |  |  |  |  |
| Jingpo (n=95) | 0 | 0 | 0.053 | 0.228 |  |  |  |  |  |  |  |  |  |  |
| Bulang (n=109) | 0.858 | 0.509 | 0.019 | 0.002 | 0 |  |  |  |  |  |  |  |  |  |
| Wa (n=109) | 0.001 | 0.014 | 0.432 | 0.983 | 0.207 | 0.001 |  |  |  |  |  |  |  |  |
| Dai (n=121) | 0 | 0.009 | 0.367 | 0.906 | 0.234 | 0 | 0.917 |  |  |  |  |  |  |  |
| Maonan (n=78) | 0.001 | 0.015 | 0.371 | 0.848 | 0.321 | 0.001 | 0.855 | 0.927 |  |  |  |  |  |  |
| Zhuang (n=101) | 0.001 | 0.017 | 0.461 | 0.989 | 0.201 | 0.001 | 0.97 | 0.888 | 0.83 |  |  |  |  |  |
| Tu (n=110) | 0.228 | 0.615 | 0.237 | 0.051 | 0.001 | 0.197 | 0.032 | 0.021 | 0.034 | 0.039 |  |  |  |  |
| Yugur (n=93) | 0.602 | 0.96 | 0.093 | 0.015 | 0 | 0.517 | 0.008 | 0.005 | 0.01 | 0.01 | 0.555 |  |  |  |
| Han-Yunnan (n=186) | 0 | 0 | 0.111 | 0.458 | 0.51 | 0 | 0.431 | 0.489 | 0.616 | 0.417 | 0.001 | 0 |  |  |
| Japanese (n=100) | 0 | 0.01 | 0.357 | 0.863 | 0.279 | 0 | 0.871 | 0.948 | 0.976 | 0.844 | 0.024 | 0.006 | 0.561 |  |
| Caucasians (n=174) | 0 | 0 | 0 | 0 | 0.03 | 0 | 0 | 0 | 0.001 | 0 | 0 | 0 | 0.001 | 0 |
| 22 | AluDPB2:AluDQA2:AluDQA1:AluDRB1:AluORF10 21212 | | | | | | | | | | | | | |
|  | Hani (n=149) | Jinuo (n=75) | Lisu (n=79) | Nu (n=82) | Jingpo (n=95) | Bulang (n=109) | Wa (n=109) | Dai (n=121) | Maonan (n=78) | Zhuang (n=101) | Tu (n=110) | Yugur (n=93) | Han-Yunnan (n=186) | Japanese (n=100) |
| Jinuo (n=75) | 0.158 |  |  |  |  |  |  |  |  |  |  |  |  |  |
| Lisu (n=79) | 0.001 | 0.065 |  |  |  |  |  |  |  |  |  |  |  |  |
| Nu (n=82) | 0.001 | 0.073 | 0.948 |  |  |  |  |  |  |  |  |  |  |  |
| Jingpo (n=95) | - | 0.26 | 0.007 | 0.008 |  |  |  |  |  |  |  |  |  |  |
| Bulang (n=109) | 0.098 | 0.793 | 0.056 | 0.064 | 0.186 |  |  |  |  |  |  |  |  |  |
| Wa (n=109) | 0.042 | 0.519 | 0.129 | 0.145 | 0.105 | 0.653 |  |  |  |  |  |  |  |  |
| Dai (n=121) | 0 | 0.007 | 0.293 | 0.257 | 0 | 0.003 | 0.008 |  |  |  |  |  |  |  |
| Maonan (n=78) | 0.001 | 0.063 | 0.982 | 0.93 | 0.006 | 0.054 | 0.124 | 0.305 |  |  |  |  |  |  |
| Zhuang (n=101) | 0.015 | 0.303 | 0.298 | 0.327 | 0.051 | 0.359 | 0.629 | 0.029 | 0.288 |  |  |  |  |  |
| Tu (n=110) | 0 | 0.021 | 0.578 | 0.527 | 0.002 | 0.012 | 0.031 | 0.577 | 0.596 | 0.094 |  |  |  |  |
| Yugur (n=93) | 0.002 | 0.103 | 0.773 | 0.824 | 0.013 | 0.097 | 0.209 | 0.158 | 0.756 | 0.439 | 0.374 |  |  |  |
| Han-Yunnan (n=186) | 0 | 0.034 | 0.79 | 0.73 | 0.004 | 0.021 | 0.052 | 0.294 | 0.811 | 0.148 | 0.694 | 0.538 |  |  |
| Japanese (n=100) | 0.003 | 0.123 | 0.677 | 0.726 | 0.016 | 0.121 | 0.253 | 0.116 | 0.661 | 0.512 | 0.299 | 0.898 | 0.44 |  |
| Caucasians (n=174) | 0.001 | 0.057 | 0.973 | 0.966 | 0.007 | 0.042 | 0.099 | 0.166 | 0.952 | 0.252 | 0.466 | 0.762 | 0.7 | 0.65 |
| 23 | AluDPB2:AluDQA2:AluDQA1:AluDRB1:AluORF10 21221 | | | | | | | | | | | | | |
|  | Hani (n=149) | Jinuo (n=75) | Lisu (n=79) | Nu (n=82) | Jingpo (n=95) | Bulang (n=109) | Wa (n=109) | Dai (n=121) | Maonan (n=78) | Zhuang (n=101) | Tu (n=110) | Yugur (n=93) | Han-Yunnan (n=186) | Japanese (n=100) |
| Jinuo (n=75) | 0.122 |  |  |  |  |  |  |  |  |  |  |  |  |  |
| Lisu (n=79) | 0.837 | 0.107 |  |  |  |  |  |  |  |  |  |  |  |  |
| Nu (n=82) | 0.317 | 0.028 | 0.504 |  |  |  |  |  |  |  |  |  |  |  |
| Jingpo (n=95) | 0.706 | 0.078 | 0.892 | 0.571 |  |  |  |  |  |  |  |  |  |  |
| Bulang (n=109) | 0.032 | 0.705 | 0.028 | 0.004 | 0.018 |  |  |  |  |  |  |  |  |  |
| Wa (n=109) | 0.004 | 0.001 | 0.031 | 0.125 | 0.029 | 0 |  |  |  |  |  |  |  |  |
| Dai (n=121) | 0.178 | 0.015 | 0.36 | 0.836 | 0.407 | 0.002 | 0.13 |  |  |  |  |  |  |  |
| Maonan (n=78) | 0.816 | 0.103 | 0.981 | 0.521 | 0.912 | 0.026 | 0.033 | 0.375 |  |  |  |  |  |  |
| Zhuang (n=101) | 0.225 | 0.019 | 0.41 | 0.895 | 0.465 | 0.002 | 0.132 | 0.94 | 0.426 |  |  |  |  |  |
| Tu (n=110) | 0.074 | 0.98 | 0.067 | 0.011 | 0.043 | 0.66 | 0 | 0.005 | 0.064 | 0.006 |  |  |  |  |
| Yugur (n=93) | 0.436 | 0.387 | 0.38 | 0.116 | 0.294 | 0.174 | 0.002 | 0.063 | 0.368 | 0.08 | 0.339 |  |  |  |
| Han-Yunnan (n=186) | 0.508 | 0.047 | 0.741 | 0.641 | 0.848 | 0.008 | 0.013 | 0.432 | 0.764 | 0.508 | 0.02 | 0.184 |  |  |
| Japanese (n=100) | 0.764 | 0.205 | 0.652 | 0.243 | 0.538 | 0.069 | 0.005 | 0.143 | 0.634 | 0.176 | 0.151 | 0.646 | 0.379 |  |
| Caucasians (n=174) | 0.019 | 0.863 | 0.019 | 0.001 | 0.01 | 0.793 | 0 | 0 | 0.018 | 0.001 | 0.822 | 0.188 | 0.003 | 0.059 |
| 24 | AluDPB2:AluDQA2:AluDQA1:AluDRB1:AluORF10 21222 | | | | | | | | | | | | | |
|  | Hani (n=149) | Jinuo (n=75) | Lisu (n=79) | Nu (n=82) | Jingpo (n=95) | Bulang (n=109) | Wa (n=109) | Dai (n=121) | Maonan (n=78) | Zhuang (n=101) | Tu (n=110) | Yugur (n=93) | Han-Yunnan (n=186) | Japanese (n=100) |
| Jinuo (n=75) | 0.756 |  |  |  |  |  |  |  |  |  |  |  |  |  |
| Lisu (n=79) | 0.095 | 0.281 |  |  |  |  |  |  |  |  |  |  |  |  |
| Nu (n=82) | 0.197 | 0.138 | 0.022 |  |  |  |  |  |  |  |  |  |  |  |
| Jingpo (n=95) | 0.32 | 0.591 | 0.535 | 0.062 |  |  |  |  |  |  |  |  |  |  |
| Bulang (n=109) | 0.005 | 0.058 | 0.372 | 0.004 | 0.115 |  |  |  |  |  |  |  |  |  |
| Wa (n=109) | 0.02 | 0.122 | 0.626 | 0.008 | 0.246 | 0.647 |  |  |  |  |  |  |  |  |
| Dai (n=121) | 0 | 0.002 | 0.021 | 0 | 0.002 | 0.093 | 0.034 |  |  |  |  |  |  |  |
| Maonan (n=78) | 0.001 | 0.022 | 0.178 | 0.001 | 0.043 | 0.572 | 0.322 | 0.337 |  |  |  |  |  |  |
| Zhuang (n=101) | 0 | 0.001 | 0.009 | 0 | 0.001 | 0.039 | 0.013 | 0.643 | 0.184 |  |  |  |  |  |
| Tu (n=110) | 0.136 | 0.086 | 0.008 | - | 0.031 | 0.001 | 0.002 | 0 | 0 | 0 |  |  |  |  |
| Yugur (n=93) | 0.558 | 0.833 | 0.341 | 0.102 | 0.724 | 0.06 | 0.138 | 0.001 | 0.021 | 0 | 0.059 |  |  |  |
| Han-Yunnan (n=186) | 0.171 | 0.434 | 0.625 | 0.045 | 0.815 | 0.089 | 0.244 | 0 | 0.025 | 0 | 0.02 | 0.536 |  |  |
| Japanese (n=100) | 0.155 | 0.101 | 0.011 | - | 0.039 | 0.001 | 0.004 | 0 | 0 | 0 | - | 0.071 | 0.027 |  |
| Caucasians (n=174) | 0 | 0.006 | 0.061 | 0 | 0.01 | 0.256 | 0.108 | 0.479 | 0.669 | 0.237 | 0 | 0.004 | 0.002 | 0 |
| 25 | AluDPB2:AluDQA2:AluDQA1:AluDRB1:AluORF10 22111 | | | | | | | | | | | | | |
|  | Hani (n=149) | Jinuo (n=75) | Lisu (n=79) | Nu (n=82) | Jingpo (n=95) | Bulang (n=109) | Wa (n=109) | Dai (n=121) | Maonan (n=78) | Zhuang (n=101) | Yugur (n=93) | Han-Yunnan (n=186) | Tu (n=110) | Japanese (n=100) |
| Tu (n=110) | 0.099 | 0.242 | 0.229 | 0.221 | 0.188 | 0.158 | 0.158 | 0.137 | 0.232 | 0.174 | 0.192 | 0.065 |  |  |
| Japanese (n=100) | 0.084 | 0.219 | 0.207 | 0.199 | 0.167 | 0.139 | 0.139 | 0.119 | 0.21 | 0.154 | 0.172 | 0.053 | 0.924 |  |
| Caucasians (n=174) | 0 | 0.002 | 0.002 | 0.001 | 0.001 | 0 | 0 | 0 | 0.002 | 0 | 0.001 | 0 | 0.003 | 0.005 |
| 26 | AluDPB2:AluDQA2:AluDQA1:AluDRB1:AluORF10 22121 | | | | | | | | | | | | | |
|  | Hani (n=149) | Jinuo (n=75) | Lisu (n=79) | Nu (n=82) | Jingpo (n=95) | Bulang (n=109) | Wa (n=109) | Dai (n=121) | Maonan (n=78) | Zhuang (n=101) | Tu (n=110) | Yugur (n=93) | Han-Yunnan (n=186) | Japanese (n=100) |
| Caucasians (n=174) | 0.19 | 0.352 | 0.34 | 0.331 | 0.295 | 0.262 | 0.262 | 0.237 | 0.343 | 0.28 | 0.26 | 0.3 | 0.143 | 0.283 |
| 27 | AluDPB2:AluDQA2:AluDQA1:AluDRB1:AluORF10 22122 | | | | | | | | | | | | | |
|  | Hani (n=149) | Jinuo (n=75) | Lisu (n=79) | Nu (n=82) | Jingpo (n=95) | Bulang (n=109) | Dai (n=121) | Maonan (n=78) | Zhuang (n=101) | Tu (n=110) | Yugur (n=93) | Han-Yunnan (n=186) | Japanese (n=100) | Wa (n=109) |
| Wa (n=109) | 0.242 | 0.406 | 0.394 | 0.385 | 0.35 | 0.317 | 0.292 | 0.397 | 0.335 | 0.315 | 0.355 | 0.191 | 0.338 |  |
| Caucasians (n=174) | 0.19 | 0.352 | 0.34 | 0.331 | 0.295 | 0.262 | 0.237 | 0.343 | 0.28 | 0.26 | 0.3 | 0.143 | 0.283 | 0.853 |
| 28 | AluDPB2:AluDQA2:AluDQA1:AluDRB1:AluORF10 22211 | | | | | | | | | | | | | |
|  | Hani (n=149) | Jinuo (n=75) | Lisu (n=79) | Nu (n=82) | Jingpo (n=95) | Bulang (n=109) | Wa (n=109) | Dai (n=121) | Maonan (n=78) | Zhuang (n=101) | Tu (n=110) | Yugur (n=93) | Han-Yunnan (n=186) | Japanese (n=100) |
| Jinuo (n=75) | - |  |  |  |  |  |  |  |  |  |  |  |  |  |
| Lisu (n=79) | 0.169 | 0.329 |  |  |  |  |  |  |  |  |  |  |  |  |
| Nu (n=82) | - | - | 0.308 |  |  |  |  |  |  |  |  |  |  |  |
| Jingpo (n=95) | 0 | 0.011 | 0.036 | 0.008 |  |  |  |  |  |  |  |  |  |  |
| Bulang (n=109) | - | - | 0.24 | - | 0.002 |  |  |  |  |  |  |  |  |  |
| Wa (n=109) | - | - | 0.24 | - | 0.002 | - |  |  |  |  |  |  |  |  |
| Dai (n=121) | 0.013 | 0.076 | 0.249 | 0.064 | 0.195 | 0.033 | 0.033 |  |  |  |  |  |  |  |
| Maonan (n=78) | - | - | 0.32 | - | 0.01 | - | - | 0.071 |  |  |  |  |  |  |
| Zhuang (n=101) | 0.224 | 0.388 | 0.861 | 0.367 | 0.014 | 0.298 | 0.298 | 0.153 | 0.379 |  |  |  |  |  |
| Tu (n=110) | 0.002 | 0.027 | 0.089 | 0.021 | 0.58 | 0.008 | 0.008 | 0.451 | 0.025 | 0.043 |  |  |  |  |
| Yugur (n=93) | 0.001 | 0.016 | 0.055 | 0.012 | 0.825 | 0.004 | 0.004 | 0.292 | 0.014 | 0.024 | 0.749 |  |  |  |
| Han-Yunnan (n=186) | - | - | 0.125 | - | 0 | - | - | 0.005 | - | 0.174 | 0.001 | 0 |  |  |
| Japanese (n=100) | - | - | 0.26 | - | 0.003 | - | - | 0.041 | - | 0.319 | 0.011 | 0.006 | - |  |
| Caucasians (n=174) | - | - | 0.137 | - | 0 | - | - | 0.007 | - | 0.189 | 0.001 | 0 | - | - |
| 29 | AluDPB2:AluDQA2:AluDQA1:AluDRB1:AluORF10 22221 | | | | | | | | | | | | | |
|  | Hani (n=149) | Jinuo (n=75) | Lisu (n=79) | Nu (n=82) | Jingpo (n=95) | Bulang (n=109) | Wa (n=109) | Dai (n=121) | Maonan (n=78) | Zhuang (n=101) | Tu (n=110) | Han-Yunnan (n=186) | Japanese (n=100) | Yugur (n=93) |
| Yugur (n=93) | 0.073 | 0.203 | 0.191 | 0.183 | 0.152 | 0.125 | 0.125 | 0.106 | 0.194 | 0.14 | 0.123 | 0.045 | 0.141 |  |
| Caucasians (n=174) | 0.354 | 0.511 | 0.5 | 0.492 | 0.46 | 0.428 | 0.428 | 0.404 | 0.503 | 0.446 | 0.426 | 0.301 | 0.448 | 0.246 |
| 30 | AluDPB2:AluDQA2:AluDQA1:AluDRB1:AluORF10 22222 | | | | | | | | | | | | | |
|  | Hani (n=149) | Jinuo (n=75) | Lisu (n=79) | Nu (n=82) | Jingpo (n=95) | Bulang (n=109) | Wa (n=109) | Dai (n=121) | Maonan (n=78) | Zhuang (n=101) | Yugur (n=93) | Han-Yunnan (n=186) | Japanese (n=100) | Caucasians (n=174) |
| Tu (n=110) | 0.019 | 0.097 | 0.088 | 0.083 | 0.062 | 0.045 | 0.045 | 0.035 | 0.09 | 0.054 | 0.065 | 0.009 | 0.055 | 0.012 |

**Supplementary Table 6. Percentage association of the POALIN with particular HLA-DRB1 alleles**

**in the POALIN/HLA class II haplotype pairs of 12 Chinese ethnic populations**

| Popu-lation | HLA Alleles | Frequency of  HLA allele | Frequency of MHC POALIN | Percentage association (%) | Frequency of MHC POALIN | Percentage association (%) | Frequency of MHC POALIN | Percentage association (%) | Frequency of MHC POALIN | Percentage association (%) | Frequency of MHC POALIN | Percentage association (%) |
| --- | --- | --- | --- | --- | --- | --- | --- | --- | --- | --- | --- | --- |
| Hani |  |  | AluDPB2*2 | | AluDQA2*2 | | AluDQA1*2 | | AluDRB1*2 | | AluORF10*2 | |
|  | DRB1*0101 | 0.013 | 0.009 | 68.18 |  |  | 0.007 | 50.00 | 0.009 | 67.96 |  |  |
|  | DRB1*0301 | 0.007 | 0.002 | 35.92 |  |  | 0.007 | 100.00 |  |  | 0.000 | 4.02 |
|  | DRB1*0403 | 0.017 | 0.017 | 100.00 |  |  | 0.013 | 79.98 |  |  |  |  |
|  | DRB1*0701 | 0.003 |  |  |  |  | 0.003 | 100.00 |  |  |  |  |
|  | DRB1*0801 | 0.003 |  |  |  |  | 0.003 | 100.00 | 0.003 | 100.00 |  |  |
|  | DRB1*0803 | 0.030 | 0.021 | 69.64 |  |  | 0.014 | 47.88 | 0.014 | 45.53 |  |  |
|  | DRB1*0901 | 0.027 | 0.022 | 80.34 |  |  | 0.027 | 100.00 |  |  |  |  |
|  | DRB1*1001 | 0.003 |  |  | 0.003 | 100.00 |  |  |  |  |  |  |
|  | DRB1*1101 | 0.013 |  |  |  |  | 0.009 | 67.29 |  |  |  |  |
|  | DRB1*1201 | 0.003 | 0.003 | 100.00 |  |  | 0.003 | 100.00 |  |  |  |  |
|  | DRB1*1202 | 0.322 | 0.144 | 44.70 |  |  | 0.286 | 88.73 | 0.035 | 10.94 |  |  |
|  | DRB1*1401 | 0.208 | 0.114 | 54.85 |  |  | 0.181 | 87.22 | 0.031 | 14.94 | 0.013 | 6.03 |
|  | DRB1*1404 | 0.117 | 0.088 | 74.51 |  |  | 0.100 | 85.39 | 0.005 | 4.21 | 0.015 | 12.52 |
|  | DRB1*1405 | 0.020 | 0.004 | 19.87 |  |  | 0.020 | 97.67 | 0.004 | 21.81 | 0.003 | 16.69 |
|  | DRB1*1410 | 0.017 | 0.007 | 39.99 |  |  | 0.013 | 79.98 | 0.005 | 28.37 | 0.004 | 26.28 |
|  | DRB1*1501 | 0.044 | 0.011 | 24.87 |  |  | 0.044 | 100.00 | 0.015 | 35.49 | 0.019 | 43.74 |
|  | DRB1*1502 | 0.070 | 0.013 | 17.97 |  |  | 0.070 | 100.00 | 0.044 | 62.78 | 0.023 | 32.38 |
|  | DRB1*1504 | 0.067 | 0.030 | 44.69 |  |  | 0.060 | 90.00 | 0.038 | 57.10 | 0.003 | 5.01 |
|  | DRB1*1602 | 0.013 | 0.009 | 68.18 |  |  | 0.013 | 100.00 | 0.007 | 50.00 | 0.003 | 25.04 |
| Jinuo |  |  | AluDPB2*2 | | AluDQA2*2 | | AluDQA1*2 | | AluDRB1*2 | | AluORF10*2 | |
|  | DRB1*0403 | 0.040 |  |  |  |  | 0.033 | 83.33 |  |  |  |  |
|  | DRB1*0701 | 0.013 | 0.007 | 50.04 | 0.007 | 50.04 | 0.013 | 100.00 | 0.007 | 50.04 |  |  |
|  | DRB1*0803 | 0.087 | 0.030 | 34.36 |  |  | 0.069 | 80.09 |  |  |  |  |
|  | DRB1*0901 | 0.007 | 0.007 | 100.00 |  |  | 0.007 | 100.00 |  |  | 0.007 | 100.00 |
|  | DRB1*1101 | 0.047 | 0.047 | 100.00 |  |  | 0.033 | 71.42 |  |  |  |  |
|  | DRB1*1202 | 0.407 | 0.193 | 47.48 |  |  | 0.407 | 100.00 |  |  |  |  |
|  | DRB1*1303 | 0.007 | 0.007 | 100.00 |  |  |  |  |  |  | 0.007 | 100.00 |
|  | DRB1*1401 | 0.153 | 0.062 | 40.39 |  |  | 0.131 | 85.17 |  |  |  |  |
|  | DRB1*1405 | 0.027 |  |  |  |  | 0.027 | 100.00 | 0.007 | 25.35 |  |  |
|  | DRB1*1501 | 0.040 | 0.022 | 54.68 |  |  | 0.040 | 100.00 | 0.040 | 100.00 | 0.025 | 62.30 |
|  | DRB1*1502 | 0.167 |  |  | 0.007 | 4.00 | 0.167 | 100.00 | 0.160 | 95.94 | 0.062 | 37.05 |
|  | DRB1*1504 | 0.007 | 0.007 | 100.00 |  |  | 0.007 | 100.00 | 0.007 | 100.00 |  |  |
| Lisu |  |  | AluDPB2*2 | | AluDQA2*2 | | AluDQA1*2 | | AluDRB1*2 | | AluORF10*2 | |
|  | DRB1*0101 | 0.013 | 0.003 | 22.27 |  |  | 0.006 | 50.00 |  |  | 0.013 | 100.00 |
|  | DRB1*0102 | 0.006 |  |  |  |  |  |  | 0.006 | 100.00 |  |  |
|  | DRB1*0401 | 0.006 |  |  |  |  | 0.006 | 100.00 |  |  |  |  |
|  | DRB1*0405 | 0.038 | 0.024 | 63.13 |  |  | 0.038 | 100.00 |  |  | 0.031 | 81.46 |
|  | DRB1*0406 | 0.006 |  |  |  |  | 0.006 | 100.00 |  |  |  |  |
|  | DRB1*0407 | 0.006 |  |  |  |  | 0.006 | 100.00 |  |  |  |  |
|  | DRB1*0410 | 0.019 | 0.013 | 66.67 |  |  | 0.019 | 100.00 |  |  | 0.006 | 33.33 |
|  | DRB1*0701 | 0.006 | 0.006 | 100.00 |  |  | 0.006 | 100.00 |  |  |  |  |
|  | DRB1*0802 | 0.006 |  |  |  |  | 0.006 | 100.00 |  |  |  |  |
|  | DRB1*0803 | 0.076 | 0.034 | 44.77 |  |  | 0.051 | 66.66 |  |  |  |  |
|  | DRB1*0901 | 0.051 | 0.016 | 31.66 |  |  | 0.051 | 100.00 |  |  |  |  |
|  | DRB1*1101 | 0.044 | 0.031 | 69.50 |  |  | 0.013 | 28.58 |  |  |  |  |
|  | DRB1*1106 | 0.006 | 0.006 | 100.00 |  |  |  |  |  |  | 0.006 | 100.00 |
|  | DRB1*1201 | 0.006 |  |  |  |  |  |  |  |  |  |  |
|  | DRB1*1202 | 0.209 | 0.055 | 26.30 |  |  | 0.209 | 100.00 |  |  |  |  |
|  | DRB1*1301 | 0.019 | 0.019 | 100.00 |  |  |  |  |  |  |  |  |
|  | DRB1*1302 | 0.013 |  |  | 0.013 | 100.00 | 0.006 | 50.00 |  |  |  |  |
|  | DRB1*1401 | 0.108 | 0.076 | 70.86 |  |  | 0.089 | 82.36 |  |  |  |  |
|  | DRB1*1404 | 0.120 | 0.113 | 93.73 | 0.006 | 5.26 | 0.076 | 63.16 |  |  |  |  |
|  | DRB1*1407 | 0.019 |  |  |  |  | 0.006 | 33.33 |  |  |  |  |
|  | DRB1*1410 | 0.013 |  |  |  |  | 0.013 | 100.00 |  |  |  |  |
|  | DRB1*1501 | 0.057 | 0.047 | 82.90 |  |  | 0.057 | 100.00 | 0.057 | 100.00 | 0.032 | 55.57 |
|  | DRB1*1502 | 0.108 | 0.013 | 11.80 |  |  | 0.108 | 100.00 | 0.101 | 94.13 | 0.039 | 35.96 |
|  | DRB1*1504 | 0.038 |  |  |  |  | 0.038 | 100.00 | 0.032 | 83.36 |  |  |
|  | DRB1*1601 | 0.006 |  |  |  |  | 0.006 | 100.00 |  |  |  |  |
| Nu |  |  | AluDPB2*2 | | AluDQA2*2 | | AluDQA1*2 | | AluDRB1*2 | | AluORF10*2 | |
|  | DRB1*0101 | 0.006 | 0.006 | 100.00 |  |  |  |  | 0.006 | 100.00 |  |  |
|  | DRB1*0403 | 0.018 |  |  |  |  | 0.018 | 100.00 |  |  |  |  |
|  | DRB1*0405 | 0.024 | 0.024 | 100.00 |  |  | 0.024 | 100.00 |  |  | 0.004 | 16.32 |
|  | DRB1*0406 | 0.030 |  |  |  |  | 0.030 | 100.00 |  |  | 0.006 | 20.01 |
|  | DRB1*0410 | 0.006 | 0.006 | 100.00 |  |  | 0.006 | 100.00 |  |  |  |  |
|  | DRB1*0701 | 0.012 | 0.012 | 100.00 |  |  | 0.012 | 100.00 |  |  | 0.005 | 44.18 |
|  | DRB1*0802 | 0.012 | 0.012 | 100.00 |  |  | 0.006 | 50.00 |  |  | 0.006 | 50.00 |
|  | DRB1*0803 | 0.110 | 0.044 | 40.13 |  |  | 0.079 | 72.22 |  |  | 0.012 | 10.74 |
|  | DRB1*0901 | 0.024 | 0.018 | 74.99 |  |  | 0.024 | 100.00 |  |  | 0.004 | 16.32 |
|  | DRB1*1101 | 0.030 | 0.022 | 71.60 |  |  | 0.006 | 20.01 |  |  | 0.003 | 9.05 |
|  | DRB1*1106 | 0.037 | 0.016 | 42.36 |  |  | 0.030 | 83.33 |  |  |  |  |
|  | DRB1*1201 | 0.018 |  |  |  |  | 0.006 | 33.35 |  |  | 0.006 | 33.35 |
|  | DRB1*1202 | 0.238 | 0.091 | 38.26 |  |  | 0.238 | 100.00 |  |  | 0.038 | 16.06 |
|  | DRB1*1301 | 0.012 |  |  |  |  |  |  |  |  |  |  |
|  | DRB1*1302 | 0.006 |  |  | 0.006 | 100.00 | 0.006 | 100.00 |  |  |  |  |
|  | DRB1*1303 | 0.012 |  |  |  |  | 0.006 | 50.00 |  |  |  |  |
|  | DRB1*1401 | 0.140 | 0.088 | 62.81 |  |  | 0.073 | 52.17 |  |  | 0.013 | 9.41 |
|  | DRB1*1403 | 0.018 | 0.018 | 100.00 |  |  |  |  |  |  |  |  |
|  | DRB1*1404 | 0.012 | 0.012 | 100.00 |  |  | 0.006 | 50.00 |  |  |  |  |
|  | DRB1*1501 | 0.037 | 0.030 | 82.97 |  |  | 0.037 | 100.00 | 0.030 | 83.33 | 0.006 | 16.67 |
|  | DRB1*1502 | 0.085 | 0.008 | 9.32 |  |  | 0.085 | 100.00 | 0.085 | 100.00 |  |  |
|  | DRB1*1504 | 0.098 |  |  |  |  | 0.098 | 100.00 | 0.098 | 100.00 |  |  |
|  | DRB1*1515 | 0.006 |  |  |  |  | 0.006 | 100.00 | 0.006 | 100.00 |  |  |
|  | DRB1*1602 | 0.006 | 0.006 | 100.00 |  |  | 0.006 | 100.00 | 0.006 | 100.00 |  |  |
| Jingpo |  |  | AluDPB2*2 | | AluDQA2*2 | | AluDQA1*2 | | AluDRB1*2 | | AluORF10*2 | |
|  | DRB1*0101 | 0.026 |  |  |  |  | 0.016 | 59.99 |  |  | 0.026 | 100.00 |
|  | DRB1*0403 | 0.005 |  |  | 0.005 | 100.00 | 0.005 | 100.00 |  |  |  |  |
|  | DRB1*0405 | 0.026 |  |  |  |  | 0.026 | 100.00 |  |  | 0.026 | 100.00 |
|  | DRB1*0406 | 0.011 |  |  | 0.005 | 49.95 | 0.011 | 100.00 |  |  |  |  |
|  | DRB1*0701 | 0.021 |  |  |  |  | 0.021 | 100.00 |  |  |  |  |
|  | DRB1*0803 | 0.047 | 0.028 | 59.70 | 0.009 | 18.39 | 0.047 | 100.00 |  |  |  |  |
|  | DRB1*0901 | 0.042 | 0.025 | 59.61 |  |  | 0.042 | 100.00 |  |  |  |  |
|  | DRB1*1001 | 0.016 |  |  | 0.010 | 65.04 | 0.016 | 100.00 |  |  |  |  |
|  | DRB1*1101 | 0.021 | 0.011 | 50.02 | 0.005 | 24.99 | 0.011 | 50.02 |  |  |  |  |
|  | DRB1*1106 | 0.016 | 0.009 | 59.72 |  |  | 0.011 | 66.69 |  |  | 0.016 | 100.00 |
|  | DRB1*1202 | 0.453 | 0.127 | 28.12 | 0.039 | 8.63 | 0.453 | 100.00 |  |  |  |  |
|  | DRB1*1302 | 0.011 | 0.011 | 100.00 | 0.011 | 100.00 | 0.011 | 100.00 |  |  |  |  |
|  | DRB1*1401 | 0.026 | 0.015 | 55.17 |  |  | 0.021 | 79.98 |  |  |  |  |
|  | DRB1*1404 | 0.021 |  |  |  |  | 0.021 | 100.00 |  |  | 0.009 | 40.95 |
|  | DRB1*1501 | 0.058 | 0.017 | 29.26 |  |  | 0.058 | 100.00 | 0.058 | 100.00 | 0.037 | 63.64 |
|  | DRB1*1502 | 0.100 | 0.014 | 14.08 | 0.011 | 10.61 | 0.100 | 100.00 | 0.100 | 100.00 | 0.028 | 28.22 |
|  | DRB1*1504 | 0.063 |  |  | 0.005 | 7.98 | 0.063 | 100.00 | 0.053 | 83.33 | 0.006 | 9.28 |
|  | DRB1*1511 | 0.011 | 0.011 | 100.00 |  |  | 0.011 | 100.00 | 0.011 | 100.00 | 0.005 | 44.25 |
|  | DRB1*1515 | 0.005 | 0.005 | 100.00 |  |  | 0.005 | 100.00 | 0.005 | 100.00 |  |  |
|  | DRB1*1602 | 0.021 | 0.006 | 30.78 | 0.005 | 24.99 | 0.021 | 100.00 | 0.021 | 100.00 | 0.021 | 100.00 |
| Bulang |  |  | AluDPB2*2 | | AluDQA2*2 | | AluDQA1*2 | | AluDRB1*2 | | AluORF10*2 | |
|  | DRB1*0401 | 0.005 | 0.005 | 100.00 |  |  | 0.005 | 100.00 |  |  |  |  |
|  | DRB1*0403 | 0.018 |  |  |  |  | 0.018 | 100.00 |  |  |  |  |
|  | DRB1*0405 | 0.023 | 0.012 | 51.35 |  |  | 0.023 | 100.00 |  |  | 0.023 | 100.00 |
|  | DRB1*0406 | 0.005 |  |  |  |  | 0.005 | 100.00 |  |  |  |  |
|  | DRB1*0701 | 0.005 |  |  |  |  | 0.005 | 100.00 |  |  | 0.005 | 100.00 |
|  | DRB1*0803 | 0.014 | 0.014 | 100.00 |  |  | 0.014 | 100.00 |  |  |  |  |
|  | DRB1*0901 | 0.014 | 0.009 | 66.64 |  |  | 0.014 | 100.00 | 0.003 | 24.56 |  |  |
|  | DRB1*1106 | 0.050 | 0.011 | 22.35 | 0.005 | 9.10 | 0.046 | 90.90 | 0.005 | 10.40 | 0.006 | 12.54 |
|  | DRB1*1202 | 0.550 | 0.270 | 49.10 |  |  | 0.550 | 100.00 | 0.010 | 1.80 | 0.012 | 2.10 |
|  | DRB1*1302 | 0.005 |  |  |  |  | 0.005 | 100.00 |  |  |  |  |
|  | DRB1*1303 | 0.005 | 0.005 | 100.00 |  |  | 0.005 | 100.00 |  |  | 0.005 | 100.00 |
|  | DRB1*1401 | 0.032 | 0.013 | 40.30 |  |  | 0.032 | 100.00 |  |  | 0.014 | 44.04 |
|  | DRB1*1404 | 0.005 | 0.005 | 100.00 |  |  | 0.005 | 100.00 |  |  |  |  |
|  | DRB1*1425 | 0.014 |  |  |  |  | 0.014 | 100.00 |  |  | 0.014 | 100.00 |
|  | DRB1*1501 | 0.073 | 0.036 | 49.31 | 0.005 | 6.25 | 0.073 | 100.00 | 0.069 | 93.70 | 0.063 | 86.43 |
|  | DRB1*1502 | 0.124 | 0.025 | 20.20 |  |  | 0.124 | 100.00 | 0.119 | 96.19 | 0.015 | 12.08 |
|  | DRB1*1504 | 0.037 | 0.007 | 20.35 | 0.014 | 37.49 | 0.037 | 100.00 | 0.032 | 87.38 | 0.013 | 36.59 |
|  | DRB1*1602 | 0.023 | 0.006 | 25.15 |  |  | 0.023 | 100.00 | 0.023 | 100.00 |  |  |
| Wa |  |  | AluDPB2*2 | | AluDQA2*2 | | AluDQA1*2 | | AluDRB1*2 | | AluORF10*2 | |
|  | DRB1*0301 | 0.018 |  |  |  |  | 0.009 | 49.97 |  |  | 0.018 | 100.00 |
|  | DRB1*0403 | 0.014 | 0.007 | 52.76 |  |  | 0.010 | 71.22 |  |  |  |  |
|  | DRB1*0405 | 0.028 |  |  |  |  | 0.028 | 100.00 | 0.004 | 15.73 | 0.016 | 59.38 |
|  | DRB1*0406 | 0.014 | 0.014 | 100.00 |  |  | 0.014 | 100.00 |  |  | 0.005 | 33.36 |
|  | DRB1*0410 | 0.009 | 0.009 | 100.00 |  |  | 0.009 | 100.00 |  |  |  |  |
|  | DRB1*0701 | 0.055 | 0.013 | 23.54 |  |  | 0.055 | 100.00 | 0.005 | 8.43 |  |  |
|  | DRB1*0803 | 0.037 | 0.023 | 63.05 |  |  | 0.032 | 87.49 | 0.004 | 11.17 |  |  |
|  | DRB1*0901 | 0.005 |  |  |  |  | 0.005 | 100.00 |  |  |  |  |
|  | DRB1*1101 | 0.005 | 0.005 | 100.00 |  |  | 0.005 | 100.00 |  |  | 0.005 | 100.00 |
|  | DRB1*1106 | 0.018 |  |  |  |  | 0.018 | 100.00 | 0.004 | 24.20 | 0.005 | 29.21 |
|  | DRB1*1202 | 0.326 | 0.145 | 44.39 |  |  | 0.326 | 100.00 | 0.006 | 1.84 | 0.007 | 2.17 |
|  | DRB1*1301 | 0.073 | 0.053 | 72.43 |  |  | 0.009 | 12.49 |  |  |  |  |
|  | DRB1*1401 | 0.014 | 0.013 | 94.48 |  |  | 0.009 | 66.64 |  |  |  |  |
|  | DRB1*1404 | 0.069 |  |  |  |  | 0.055 | 80.00 |  |  |  |  |
|  | DRB1*1501 | 0.092 | 0.030 | 32.95 |  |  | 0.092 | 100.00 | 0.087 | 94.87 | 0.049 | 53.61 |
|  | DRB1*1502 | 0.087 | 0.035 | 40.32 | 0.005 | 5.27 | 0.082 | 94.02 | 0.078 | 89.28 | 0.018 | 20.84 |
|  | DRB1*1504 | 0.133 | 0.093 | 70.19 |  |  | 0.133 | 100.00 | 0.124 | 92.89 | 0.009 | 7.06 |
|  | DRB1*1602 | 0.005 |  |  |  |  | 0.005 | 100.00 | 0.005 | 100.00 | 0.005 | 100.00 |
| Dai |  |  | AluDPB2*2 | | AluDQA2*2 | | AluDQA1*2 | | AluDRB1*2 | | AluORF10*2 | |
|  | DRB1*0301 | 0.058 | 0.023 | 39.00 |  |  | 0.054 | 92.86 | 0.034 | 58.72 | 0.034 | 59.29 |
|  | DRB1*0403 | 0.012 | 0.008 | 66.61 |  |  | 0.012 | 100.00 |  |  |  |  |
|  | DRB1*0405 | 0.054 | 0.026 | 48.18 |  |  | 0.054 | 100.00 | 0.017 | 32.24 | 0.014 | 25.61 |
|  | DRB1*0406 | 0.021 | 0.009 | 44.00 | 0.004 | 19.99 | 0.021 | 100.00 |  |  | 0.008 | 39.98 |
|  | DRB1*0701 | 0.008 | 0.006 | 73.37 |  |  | 0.008 | 100.00 |  |  | 0.002 | 25.18 |
|  | DRB1*0803 | 0.004 | 0.004 | 100.00 |  |  |  |  |  |  |  |  |
|  | DRB1*0809 | 0.004 |  |  |  |  | 0.004 | 100.00 | 0.004 | 100.00 | 0.004 | 100.00 |
|  | DRB1*0901 | 0.112 | 0.057 | 51.02 | 0.004 | 3.70 | 0.099 | 88.89 | 0.014 | 12.57 | 0.044 | 39.23 |
|  | DRB1*1001 | 0.008 | 0.004 | 50.00 |  |  | 0.008 | 100.00 | 0.008 | 100.00 | 0.008 | 100.00 |
|  | DRB1*1101 | 0.021 | 0.015 | 73.62 | 0.008 | 39.98 | 0.017 | 80.01 |  |  |  |  |
|  | DRB1*1106 | 0.004 |  |  |  |  | 0.004 | 100.00 | 0.004 | 100.00 | 0.004 | 100.00 |
|  | DRB1*1202 | 0.099 | 0.040 | 40.66 | 0.007 | 6.96 | 0.099 | 100.00 | 0.028 | 28.69 | 0.027 | 26.97 |
|  | DRB1*1302 | 0.025 | 0.016 | 64.30 | 0.004 | 16.66 | 0.021 | 83.34 | 0.009 | 37.03 | 0.025 | 100.00 |
|  | DRB1*1303 | 0.037 |  |  | 0.008 | 22.21 | 0.033 | 88.89 | 0.016 | 43.00 | 0.012 | 32.00 |
|  | DRB1*1401 | 0.112 | 0.085 | 75.86 | 0.000 | 0.04 | 0.107 | 96.30 | 0.066 | 58.93 | 0.028 | 24.94 |
|  | DRB1*1404 | 0.033 |  |  |  |  | 0.033 | 100.00 | 0.005 | 14.58 | 0.010 | 30.67 |
|  | DRB1*1405 | 0.012 |  |  |  |  | 0.012 | 100.00 | 0.012 | 100.00 | 0.007 | 57.66 |
|  | DRB1*1501 | 0.087 | 0.011 | 12.23 |  |  | 0.083 | 95.23 | 0.048 | 55.49 | 0.044 | 50.98 |
|  | DRB1*1502 | 0.136 | 0.069 | 50.84 |  |  | 0.128 | 93.94 | 0.061 | 44.93 | 0.045 | 33.05 |
|  | DRB1*1602 | 0.153 | 0.081 | 53.27 | 0.005 | 3.57 | 0.153 | 100.00 | 0.040 | 26.11 | 0.051 | 33.49 |
| Maonan |  |  | AluDPB2*2 | | AluDQA2*2 | | AluDQA1*2 | | AluDRB1*2 | | AluORF10*2 | |
|  | DRB1*0301 | 0.045 |  |  |  |  | 0.045 | 100.00 | 0.007 | 15.24 | 0.045 | 100.00 |
|  | DRB1*0403 | 0.006 |  |  |  |  | 0.006 | 100.00 |  |  |  |  |
|  | DRB1*0404 | 0.013 |  |  |  |  | 0.013 | 100.00 |  |  |  |  |
|  | DRB1*0405 | 0.090 | 0.016 | 18.35 |  |  | 0.090 | 100.00 |  |  | 0.075 | 83.51 |
|  | DRB1*0406 | 0.006 |  |  |  |  | 0.006 | 100.00 |  |  |  |  |
|  | DRB1*0803 | 0.071 | 0.012 | 16.62 |  |  | 0.071 | 100.00 |  |  |  |  |
|  | DRB1*0901 | 0.103 | 0.075 | 73.53 |  |  | 0.103 | 100.00 |  |  | 0.025 | 23.90 |
|  | DRB1*1101 | 0.026 | 0.014 | 53.71 |  |  | 0.019 | 75.00 | 0.006 | 25.00 | 0.013 | 50.00 |
|  | DRB1*1202 | 0.160 | 0.034 | 21.42 |  |  | 0.160 | 100.00 | 0.013 | 8.27 |  |  |
|  | DRB1*1303 | 0.064 | 0.007 | 10.41 |  |  | 0.064 | 100.00 | 0.013 | 20.34 | 0.043 | 67.36 |
|  | DRB1*1401 | 0.122 | 0.071 | 57.94 |  |  | 0.109 | 89.47 |  |  |  |  |
|  | DRB1*1404 | 0.006 |  |  |  |  | 0.006 | 100.00 |  |  | 0.006 | 100.00 |
|  | DRB1*1405 | 0.013 | 0.013 | 100.00 |  |  | 0.006 | 50.00 |  |  |  |  |
|  | DRB1*1501 | 0.096 | 0.030 | 31.68 |  |  | 0.096 | 100.00 | 0.096 | 100.00 | 0.044 | 45.81 |
|  | DRB1*1502 | 0.058 | 0.021 | 36.31 |  |  | 0.058 | 100.00 | 0.051 | 87.99 | 0.029 | 50.20 |
|  | DRB1*1602 | 0.122 | 0.066 | 54.04 |  |  | 0.122 | 100.00 | 0.108 | 89.01 | 0.079 | 65.05 |
| Zhuang |  |  | AluDPB2*2 | | AluDQA2*2 | | AluDQA1*2 | | AluDRB1*2 | | AluORF10*2 | |
|  | DRB1*0301 | 0.129 | 0.013 | 10.36 |  |  | 0.070 | 54.01 |  |  | 0.116 | 90.14 |
|  | DRB1*0405 | 0.015 | 0.000 | 1.89 |  |  | 0.015 | 100.00 |  |  |  |  |
|  | DRB1*0406 | 0.015 | 0.015 | 100.00 |  |  | 0.015 | 100.00 |  |  |  |  |
|  | DRB1*0803 | 0.015 |  |  |  |  | 0.015 | 100.00 |  |  |  |  |
|  | DRB1*0901 | 0.050 | 0.042 | 84.75 |  |  | 0.050 | 100.00 |  |  | 0.009 | 18.61 |
|  | DRB1*1001 | 0.020 |  |  |  |  | 0.005 | 25.00 |  |  |  |  |
|  | DRB1*1101 | 0.050 | 0.024 | 48.67 |  |  | 0.040 | 80.00 |  |  |  |  |
|  | DRB1*1202 | 0.045 | 0.037 | 83.79 |  |  | 0.045 | 100.00 |  |  |  |  |
|  | DRB1*1301 | 0.005 | 0.005 | 100.00 |  |  |  |  |  |  |  |  |
|  | DRB1*1302 | 0.015 |  |  | 0.010 | 66.67 | 0.015 | 100.00 | 0.005 | 33.33 | 0.005 | 33.33 |
|  | DRB1*1303 | 0.025 |  |  |  |  | 0.015 | 60.00 | 0.005 | 20.00 | 0.011 | 44.73 |
|  | DRB1*1401 | 0.139 | 0.093 | 66.84 |  |  | 0.109 | 78.57 |  |  |  |  |
|  | DRB1*1404 | 0.005 |  |  |  |  | 0.005 | 100.00 |  |  | 0.005 | 100.00 |
|  | DRB1*1405 | 0.005 |  |  |  |  | 0.005 | 100.00 |  |  |  |  |
|  | DRB1*1418 | 0.015 | 0.015 | 100.00 |  |  | 0.015 | 100.00 |  |  |  |  |
|  | DRB1*1501 | 0.168 | 0.073 | 43.12 |  |  | 0.168 | 100.00 | 0.168 | 100.00 | 0.056 | 33.33 |
|  | DRB1*1502 | 0.139 | 0.065 | 46.79 | 0.015 | 10.71 | 0.133 | 96.28 | 0.134 | 96.43 | 0.070 | 50.63 |
|  | DRB1*1602 | 0.149 | 0.044 | 29.65 |  |  | 0.149 | 100.00 | 0.144 | 96.67 | 0.094 | 63.20 |
| Tu |  |  | AluDPB2*2 | | AluDQA2*2 | | AluDQA1*2 | | AluDRB1*2 | | AluORF10*2 | |
|  | DRB1*0101 | 0.064 | 0.040 | 62.37 | 0.010 | 15.12 | 0.049 | 77.58 | 0.023 | 35.72 | 0.005 | 7.15 |
|  | DRB1*0301 | 0.023 | 0.023 | 100.00 | 0.010 | 42.67 | 0.014 | 60.01 |  |  | 0.009 | 39.99 |
|  | DRB1*0401 | 0.027 | 0.010 | 37.92 |  |  | 0.027 | 100.00 |  |  |  |  |
|  | DRB1*0403 | 0.005 |  |  |  |  | 0.005 | 100.00 |  |  |  |  |
|  | DRB1*0404 | 0.005 |  |  |  |  | 0.005 | 100.00 |  |  |  |  |
|  | DRB1*0405 | 0.068 | 0.050 | 73.54 | 0.016 | 23.42 | 0.068 | 100.00 |  |  |  |  |
|  | DRB1*0406 | 0.014 |  |  | 0.005 | 33.36 | 0.014 | 100.00 |  |  | 0.008 | 56.09 |
|  | DRB1*0410 | 0.009 | 0.009 | 100.00 |  |  | 0.009 | 100.00 |  |  |  |  |
|  | DRB1*0701 | 0.073 | 0.020 | 27.60 | 0.008 | 11.43 | 0.073 | 100.00 |  |  |  |  |
|  | DRB1*0802 | 0.005 | 0.005 | 100.00 |  |  | 0.005 | 100.00 |  |  |  |  |
|  | DRB1*0803 | 0.059 | 0.044 | 73.70 |  |  | 0.039 | 65.43 |  |  |  |  |
|  | DRB1*0901 | 0.127 | 0.078 | 61.65 |  |  | 0.127 | 100.00 |  |  | 0.029 | 22.61 |
|  | DRB1*1001 | 0.045 | 0.014 | 30.36 | 0.026 | 57.49 | 0.023 | 50.01 |  |  |  |  |
|  | DRB1*1101 | 0.105 | 0.038 | 36.24 |  |  | 0.064 | 60.87 |  |  | 0.025 | 24.14 |
|  | DRB1*1104 | 0.005 |  |  |  |  |  |  | 0.005 | 100.00 |  |  |
|  | DRB1*1201 | 0.077 | 0.029 | 37.05 |  |  | 0.045 | 58.82 |  |  | 0.011 | 14.64 |
|  | DRB1*1202 | 0.086 | 0.041 | 46.93 | 0.006 | 6.50 | 0.086 | 100.00 |  |  | 0.015 | 17.94 |
|  | DRB1*1301 | 0.014 |  |  |  |  |  |  |  |  | 0.005 | 33.36 |
|  | DRB1*1302 | 0.018 | 0.018 | 99.94 | 0.005 | 25.03 | 0.005 | 25.03 |  |  |  |  |
|  | DRB1*1401 | 0.045 | 0.029 | 64.31 | 0.009 | 20.00 | 0.030 | 64.95 |  |  |  |  |
|  | DRB1*1402 | 0.009 | 0.009 | 100.00 |  |  | 0.005 | 50.06 |  |  |  |  |
|  | DRB1*1403 | 0.014 | 0.014 | 100.00 |  |  |  |  |  |  |  |  |
|  | DRB1*1405 | 0.027 | 0.019 | 68.54 | 0.009 | 33.33 | 0.010 | 35.64 |  |  |  |  |
|  | DRB1*1501 | 0.018 | 0.005 | 25.03 | 0.006 | 35.64 | 0.018 | 100.00 | 0.018 | 100.00 |  |  |
|  | DRB1*1502 | 0.041 | 0.012 | 28.62 | 0.014 | 33.34 | 0.041 | 100.00 | 0.041 | 100.00 | 0.021 | 50.43 |
|  | DRB1*1601 | 0.009 |  |  | 0.005 | 50.06 | 0.009 | 100.00 | 0.009 | 100.00 | 0.009 | 100.00 |
|  | DRB1*1602 | 0.009 | 0.009 | 100.00 |  |  | 0.009 | 100.00 | 0.005 | 50.06 | 0.005 | 50.06 |
| Yugur |  |  | AluDPB2*2 | | AluDQA2*2 | | AluDQA1*2 | | AluDRB1*2 | | AluORF10*2 | |
|  | DRB1*0101 | 0.048 | 0.016 | 33.42 | 0.012 | 24.30 | 0.040 | 82.77 | 0.048 | 100.00 |  |  |
|  | DRB1*0301 | 0.065 | 0.013 | 19.79 | 0.005 | 8.34 | 0.059 | 91.66 |  |  | 0.028 | 43.32 |
|  | DRB1*0401 | 0.043 |  |  |  |  | 0.043 | 100.00 |  |  |  |  |
|  | DRB1*0402 | 0.005 | 0.005 | 100.00 |  |  | 0.005 | 100.00 |  |  |  |  |
|  | DRB1*0403 | 0.038 |  |  |  |  | 0.038 | 100.00 |  |  |  |  |
|  | DRB1*0404 | 0.016 |  |  |  |  | 0.016 | 100.00 |  |  |  |  |
|  | DRB1*0405 | 0.054 | 0.054 | 100.00 |  |  | 0.054 | 100.00 |  |  | 0.047 | 87.43 |
|  | DRB1*0406 | 0.022 | 0.013 | 61.04 |  |  | 0.022 | 100.00 | 0.005 | 25.01 |  |  |
|  | DRB1*0408 | 0.011 | 0.011 | 100.00 |  |  | 0.007 | 61.02 |  |  |  |  |
|  | DRB1*0701 | 0.065 | 0.028 | 42.70 | 0.016 | 25.00 | 0.065 | 100.00 |  |  | 0.013 | 19.61 |
|  | DRB1*0801 | 0.016 |  |  |  |  | 0.016 | 100.00 |  |  |  |  |
|  | DRB1*0802 | 0.022 |  |  |  |  | 0.022 | 100.00 |  |  |  |  |
|  | DRB1*0803 | 0.027 | 0.009 | 32.59 |  |  | 0.022 | 80.02 |  |  |  |  |
|  | DRB1*0804 | 0.011 | 0.011 | 100.00 | 0.011 | 100.00 | 0.011 | 100.00 |  |  |  |  |
|  | DRB1*0809 | 0.005 |  |  |  |  | 0.005 | 100.00 |  |  |  |  |
|  | DRB1*0901 | 0.134 | 0.090 | 66.67 |  |  | 0.134 | 100.00 |  |  |  |  |
|  | DRB1*1001 | 0.054 | 0.036 | 66.42 | 0.031 | 58.13 | 0.046 | 85.51 |  |  |  |  |
|  | DRB1*1101 | 0.048 | 0.035 | 73.05 | 0.005 | 11.12 | 0.032 | 66.67 |  |  |  |  |
|  | DRB1*1104 | 0.005 | 0.005 | 100.00 |  |  | 0.005 | 100.00 |  |  |  |  |
|  | DRB1*1201 | 0.054 | 0.046 | 84.78 |  |  | 0.054 | 100.00 |  |  |  |  |
|  | DRB1*1202 | 0.054 | 0.028 | 52.33 |  |  | 0.054 | 100.00 | 0.005 | 10.10 |  |  |
|  | DRB1*1301 | 0.043 |  |  | 0.005 | 12.51 |  |  |  |  |  |  |
|  | DRB1*1401 | 0.043 | 0.017 | 40.53 |  |  | 0.036 | 84.77 |  |  |  |  |
|  | DRB1*1403 | 0.005 |  |  |  |  | 0.005 | 100.00 |  |  |  |  |
|  | DRB1*1405 | 0.016 | 0.006 | 36.45 |  |  | 0.016 | 100.00 |  |  |  |  |
|  | DRB1*1501 | 0.065 | 0.039 | 60.97 | 0.005 | 8.34 | 0.065 | 100.00 | 0.059 | 91.57 | 0.020 | 30.89 |
|  | DRB1*1502 | 0.022 | 0.006 | 29.29 |  |  | 0.022 | 100.00 | 0.022 | 100.00 | 0.005 | 25.01 |
|  | DRB1*1602 | 0.011 | 0.005 | 50.05 |  |  | 0.011 | 100.00 | 0.005 | 50.05 | 0.005 | 50.05 |

**Supplementary Table 7. Percentage association between HLA-DRB1 alleles and the Alu class II POALIN**

| DRB1 Allele | Percentage Association in 15 Populations (%) | | | | | | | | | | | | | | |
| --- | --- | --- | --- | --- | --- | --- | --- | --- | --- | --- | --- | --- | --- | --- | --- |
| AluDPB2 | | | | | | | | | | | | | | | |
|  | Hani | Jinuo | Lisu | Nu | Jingpo | Bulang | Wa | Dai | Maonan | Zhuang | Tu | Yugur | Han-Yunnan^a^ | Japanese^b^ | Caucasian^b^ |
| DRB1*01 | 68.2 |  | 14.8 | 100.0 |  |  |  |  |  |  | 62.4 | 33.4 | 22.9 | 81.3 | 59 |
| DRB1*03 | 35.9 |  |  |  |  |  |  | 39.0 |  | 10.4 | 100.0 | 19.8 | 19.5 |  | 35.1 |
| DRB1*04 | 100.0 |  | 48.2 | 38.5 |  | 32.4 | 47.0 | 49.8 | 14.3 | 50.9 | 54.7 | 44.1 | 56.8 | 59.9 | 29.2 |
| DRB1*07 |  | 50.0 | 100.0 | 100.0 |  |  | 23.5 | 73.4 |  |  | 27.6 | 42.7 | 29.8 |  | 51.6 |
| DRB1*08 | 62.7 | 34.4 | 41.3 | 46.1 | 59.7 | 100.0 | 63.1 | 50.0 | 16.6 |  | 75.6 | 24.2 | 39.7 | 50.1 | 3 |
| DRB1*09 | 80.3 | 100.0 | 31.7 | 75.0 | 59.6 | 66.6 |  | 51.0 | 73.5 | 84.7 | 61.6 | 66.7 | 58 | 57 | 74.8 |
| DRB1*10 |  |  |  |  |  |  |  | 50.0 |  |  | 30.4 | 66.4 |  | 100 |  |
| DRB1*11 |  | 100.0 | 73.3 | 55.6 | 54.2 | 22.4 | 20.0 | 61.4 | 53.7 | 48.7 | 34.7 | 75.7 | 76.4 |  | 33 |
| DRB1*12 | 45.3 | 47.5 | 25.5 | 35.5 | 28.1 | 49.1 | 44.4 | 40.7 | 21.4 | 83.8 | 42.3 | 68.6 | 32.7 | 65.3 |  |
| DRB1*13 |  | 100.0 | 60.0 |  | 100.0 | 50.0 | 72.4 | 25.7 | 10.4 | 11.1 | 57.1 |  | 36.5 |  | 38 |
| DRB1*14 | 58.6 | 34.4 | 72.8 | 69.5 | 30.7 | 34.7 | 15.7 | 53.9 | 59.1 | 65.8 | 74.0 | 36.1 | 77.6 | 43.6 | 66.9 |
| DRB1*15 | 29.5 | 13.4 | 29.6 | 17.0 | 19.8 | 29.4 | 50.9 | 35.8 | 33.4 | 44.8 | 27.5 | 53.1 | 34.4 | 27.4 | 63.7 |
| DRB1*16 | 68.2 |  |  | 100.0 | 30.8 | 25.2 |  | 53.3 | 54.0 | 29.7 | 50.0 | 50.0 | 57.7 |  | 59.7 |
| AluDQA2 | | | | | | | | | | | | | | | |
|  | Hani | Jinuo | Lisu | Nu | Jingpo | Bulang | Wa | Dai | Maonan | Zhuang | Tu | Yugur | Han-Yunnan^a^ | Japanese^b^ | Caucasian^b^ |
| DRB1*01 |  |  |  |  |  |  |  |  |  |  | 15.1 | 24.3 |  | 6.3 | 15.7 |
| DRB1*03 |  |  |  |  |  |  |  |  |  |  | 42.7 | 8.3 |  |  | 76 |
| DRB1*04 |  |  |  |  | 25.0 |  |  | 4.8 |  |  | 16.1 |  |  |  | 1.6 |
| DRB1*07 |  | 50.0 |  |  |  |  |  |  |  |  | 11.4 | 25.0 |  |  | 17.4 |
| DRB1*08 |  |  |  |  | 18.4 |  |  |  |  |  |  | 13.3 |  |  | 54.2 |
| DRB1*09 |  |  |  |  |  |  |  | 3.7 |  |  |  |  |  |  | 29.1 |
| DRB1*10 | 100.0 |  |  |  | 65.0 |  |  |  |  |  | 57.5 | 58.1 |  | 100 | 98.3 |
| DRB1*11 |  |  |  |  | 14.3 | 9.1 |  | 33.3 |  |  |  | 10.0 |  |  |  |
| DRB1*12 |  |  |  |  | 8.6 |  |  | 7.0 |  |  | 3.4 |  |  |  |  |
| DRB1*13 |  |  | 40.0 | 20.0 | 100.0 |  |  | 20.0 |  | 22.2 | 14.3 | 12.5 |  |  | 35.6 |
| DRB1*14 |  |  | 2.4 |  |  |  |  | 0.0 |  |  | 19.0 |  |  |  | 8.1 |
| DRB1*15 |  | 3.1 |  |  | 6.6 | 7.8 | 1.5 |  |  | 4.8 | 34.0 | 6.3 | 2.3 |  |  |
| DRB1*16 |  |  |  |  | 25.0 |  |  | 3.6 |  |  | 25.0 |  |  |  | 20.1 |
| AluDQA1 | | | | | | | | | | | | | | | |
|  | Hani | Jinuo | Lisu | Nu | Jingpo | Bulang | Wa | Dai | Maonan | Zhuang | Tu | Yugur | Han-Yunnan^a^ | Japanese^b^ | Caucasian^b^ |
| DRB1*01 | 50.0 |  | 33.3 |  | 60.0 |  |  |  |  |  | 77.6 | 82.8 | 66.7 |  | 2.4 |
| DRB1*03 | 100.0 |  |  |  |  |  | 50.0 | 92.9 | 100.0 | 54.0 | 60.0 | 91.7 | 7.4 |  |  |
| DRB1*04 | 80.0 | 83.3 | 100.0 | 100.0 | 100.0 | 100.0 | 93.8 | 100.0 | 100.0 | 100.0 | 100.0 | 97.8 | 100 | 73.5 | 92.3 |
| DRB1*07 | 100.0 | 100.0 | 100.0 | 100.0 | 100.0 | 100.0 | 100.0 | 100.0 |  |  | 100.0 | 100.0 | 86.6 |  | 100 |
| DRB1*08 | 53.1 | 80.1 | 69.2 | 70.0 | 100.0 | 100.0 | 87.5 | 50.0 | 100.0 | 100.0 | 67.9 | 93.3 | 20.4 | 24.1 | 100 |
| DRB1*09 | 100.0 | 100.0 | 100.0 | 100.0 | 100.0 | 100.0 | 100.0 | 88.9 | 100.0 | 100.0 | 100.0 | 100.0 | 94.8 | 79.9 | 100 |
| DRB1*10 |  |  |  |  | 100.0 |  |  | 100.0 |  | 25.0 | 50.0 | 85.5 |  |  |  |
| DRB1*11 | 67.3 | 71.4 | 25.0 | 54.5 | 57.2 | 90.9 | 100.0 | 83.3 | 75.0 | 80.0 | 58.3 | 70.0 | 8.4 |  | 3.5 |
| DRB1*12 | 88.8 | 100.0 | 97.1 | 95.2 | 100.0 | 100.0 | 100.0 | 100.0 | 100.0 | 100.0 | 80.6 | 100.0 | 86.3 | 77.8 |  |
| DRB1*13 |  |  | 20.0 | 40.0 | 100.0 | 100.0 | 12.5 | 86.7 | 100.0 | 66.7 | 14.3 |  | 19.5 |  | 2.5 |
| DRB1*14 | 86.9 | 87.4 | 70.7 | 46.4 | 88.9 | 100.0 | 77.8 | 97.4 | 86.4 | 81.8 | 45.9 | 89.8 | 39.1 |  |  |
| DRB1*15 | 96.3 | 100.0 | 100.0 | 100.0 | 100.0 | 100.0 | 98.3 | 94.4 | 100.0 | 98.3 | 100.0 | 100.0 | 97.6 | 77.1 | 100 |
| DRB1*16 | 100.0 |  | 100.0 | 100.0 | 100.0 | 100.0 | 100.0 | 100.0 | 100.0 | 100.0 | 100.0 | 100.0 | 86.3 | 100 | 100 |
| AluDRB1 | | | | | | | | | | | | | | | |
|  | Hani | Jinuo | Lisu | Nu | Jingpo | Bulang | Wa | Dai | Maonan | Zhuang | Tu | Yugur | Han-Yunnan^a^ | Japanese^b^ | Caucasian^b^ |
| DRB1*01 | 68.0 |  | 33.3 | 100.0 |  |  |  |  |  |  | 35.7 | 100.0 | 100 | 75 | 100 |
| DRB1*03 |  |  |  |  |  |  |  | 58.7 | 15.2 |  |  |  | 2.7 |  |  |
| DRB1*04 |  |  |  |  |  |  | 6.7 | 20.0 |  |  |  | 2.9 | 8.7 | 2.2 | 4.6 |
| DRB1*07 |  | 50.0 |  |  |  |  | 8.4 |  |  |  |  |  |  |  |  |
| DRB1*08 | 51.0 |  |  |  |  |  | 11.2 | 50.0 |  |  |  |  | 5.7 | 4.8 |  |
| DRB1*09 |  |  |  |  |  | 24.6 |  | 12.6 |  |  |  |  | 3.4 |  |  |
| DRB1*10 |  |  |  |  |  |  |  | 100.0 |  |  |  |  |  |  | 49.6 |
| DRB1*11 |  |  |  |  |  | 10.4 | 19.4 | 16.7 | 25.0 |  | 4.2 |  |  |  |  |
| DRB1*12 | 10.8 |  |  |  |  | 1.8 | 1.8 | 28.7 | 8.3 |  |  | 5.1 | 4.4 |  |  |
| DRB1*13 |  |  |  |  |  |  |  | 40.6 | 20.3 | 22.2 |  |  | 5.3 |  |  |
| DRB1*14 | 12.5 | 3.8 |  |  |  |  |  | 52.8 |  |  |  |  | 8.9 |  |  |
| DRB1*15 | 54.1 | 96.8 | 93.8 | 97.3 | 95.6 | 94.0 | 92.5 | 49.0 | 95.5 | 98.4 | 100.0 | 93.7 | 85.5 | 82.6 | 100 |
| DRB1*16 | 50.0 |  |  | 100.0 | 100.0 | 100.0 | 100.0 | 26.1 | 89.0 | 96.7 | 75.0 | 50.0 | 86.6 | 100 | 100 |
| AluORF10 | | | | | | | | | | | | | | | |
|  | Hani | Jinuo | Lisu | Nu | Jingpo | Bulang | Wa | Dai | Maonan | Zhuang | Tu | Yugur | Han-Yunnan^a^ | Japanese^b^ | Caucasian^b^ |
| DRB1*01 |  |  | 66.7 |  | 100.0 |  |  |  |  |  | 7.1 |  | 6 | 6.3 | 12.3 |
| DRB1*03 | 4.0 |  |  |  |  |  | 100.0 | 59.3 | 100.0 | 90.1 | 40.0 | 43.3 | 55.6 |  | 1.6 |
| DRB1*04 |  |  | 49.1 | 12.7 | 62.5 | 45.5 | 32.6 | 25.4 | 65.0 |  | 6.0 | 25.0 | 49 | 53.1 | 3.5 |
| DRB1*07 |  |  |  | 44.2 |  | 100.0 |  | 25.2 |  |  |  | 19.6 |  |  | 62 |
| DRB1*08 |  |  |  | 14.7 |  |  |  | 50.0 |  |  |  |  | 5.6 |  |  |
| DRB1*09 |  | 100.0 |  | 16.3 |  |  |  | 39.2 | 23.9 | 18.6 | 22.6 |  | 3.1 |  | 24 |
| DRB1*10 |  |  |  |  |  |  |  | 100.0 |  |  |  |  |  |  |  |
| DRB1*11 |  |  | 12.5 | 4.1 | 42.9 | 12.5 | 43.4 | 16.7 | 50.0 |  | 23.1 |  | 6 |  |  |
| DRB1*12 |  |  |  | 17.3 |  | 2.1 | 2.2 | 27.0 |  |  | 16.4 |  | 7.4 |  |  |
| DRB1*13 |  | 100.0 |  |  |  | 50.0 |  | 59.2 | 67.4 | 36.0 | 14.3 |  |  |  | 10.2 |
| DRB1*14 | 9.7 |  |  | 7.7 | 18.2 | 55.3 |  | 28.7 | 4.5 | 3.0 |  |  | 4.6 |  |  |
| DRB1*15 | 25.0 | 40.6 | 34.7 | 2.7 | 31.9 | 39.2 | 24.6 | 40.0 | 47.5 | 41.1 | 34.9 | 29.4 | 43.3 |  | 89.1 |
| DRB1*16 | 25.0 |  |  |  | 100.0 |  | 100.0 | 33.5 | 65.1 | 63.2 | 75.0 | 50.0 | 64.4 | 100 |  |

^a^: The data of five POALINs in Han-Yunnan were published previously (Shi et al. 2014)

^b^: The data of five POALINs in Japanese and Caucasian were published previously (Kulski et al. 2010, 2011)
